# Data on cytotoxic and antibacterial activity of synthesized Fe_3_O_4_ nanoparticles using *Malva sylvestris*

**DOI:** 10.1016/j.dib.2019.104929

**Published:** 2019-12-09

**Authors:** Seyyed Mojtaba Mousavi, Seyyed Alireza Hashemi, Maryam Zarei, Sonia Bahrani, Amir Savardashtaki, Hossein Esmaeili, Chin Wei Lai, Sargol Mazraedoost, Mohsen Abassi, Bahman Ramavandi

**Affiliations:** a Department of Medical Nanotechnology, School of Advanced Medical Sciences and Technologies, Shiraz University of Medical Sciences, Shiraz, Iran; b Nanotechnology & Catalysis Research Center, University of Malaya, Malaysia; c Department of Mechanical Engineering, Center for Nanofibers and Nanotechnology, National University of Singapore, Singapore; d Department of Medical Biotechnology, School of Advanced Medical Sciences and Technologies, Shiraz University of Medical Sciences, Shiraz, Iran; e Department of Chemical Engineering, Bushehr Branch, Islamic Azad University, Bushehr, Iran; f Department of Environmental Health Engineering, Faculty of Health and Nutrition, Bushehr University of Medical Sciences, Bushehr, Iran; g Systems Environmental Health and Energy Research Center, The Persian Gulf Biomedical Sciences Research Institute, Bushehr University of Medical Sciences, Bushehr, Iran

**Keywords:** *Malva sylvestris*, Antibacterial, Fe_3_O_4_ nanoparticles, Anticancer, Cytotoxicity performance

## Abstract

The biosynthesis of materials using medicinal plants can be a low-cost and eco-friendly approach due to their extraordinary properties. Herein, we reported a facile synthesis of Fe_3_O_4_ nanoparticles using *Malva sylvestris*. The surface morphology, functional groups, and elemental analysis were done to characterize the synthesized nanoparticles. The cytotoxicity performance of the synthesized nanoparticles was analyzed by exposing nanoparticles to MCF-7 and Hep-G2 cancer cell lines through MTT colorimetric assay and the IC50 value was defined as 100 μg/mL and 200 μg/mL, respectively. The antibacterial performance of synthesized nanoparticles against four different bacterial strains including *Staphylococcus aureus, Corynebacterium, Pseudomonas aeruginosa,* and *Klebsiella pneumoniae* were assessed through microdilution broth method. The synthesized Fe_3_O_4_ nanoparticles using *Malva sylvestris* demonstrated higher antibacterial effects against Gram-positive strains with MIC values of 62.5 μg/mL and 125 μg/mL which increase the inhibitory percentage to more than 90%.

**Table:** Specifications Table

Subject	Biology
Specific subject area	Developmental biology
Type of data	Figure and table
How data were acquired	- Cytotoxic and antibacterial tests. - X-Ray Diffraction (XRD) - Scanning Electron Microscopy (SEM) - Energy Dispersive X-Ray Spectroscopy (EDX) - Fourier-transform infrared spectroscopy (FT-IR).
Data format	Raw
Parameters for data collection	MIC and MBC methods for antibacterial tests and MTT assay for cytotoxic tests.
Description of data collection	The effects of cytotoxic and antibacterial activity of synthesized Fe_3_O_4_ nanoparticles using *Malva sylvestris* were evaluated on different microorganisms by MIC and MBC methods. The cytotoxic effects were determined using two different cancer cell lines.
Data source location	Department of Medical Nanotechnology, School of Advanced Medical Sciences and Technologies, Shiraz University of Medical Sciences, Shiraz, Iran
Data accessibility	Data are provided in this article. The raw data source for this article is available in the Supplementary section.

**Table:** 

**Value of the Data** • This research analyzes the cytotoxic and antibacterial effects of synthesized Fe_3_O_4_ nanoparticles using *Malva sylvestris* against four different microorganisms and two different cancer cell lines. Biologists, toxicologists, and pharmacists can pay close attention to the results of this study. • Data from this work describe the anticancer activity of synthesized Fe_3_O_4_ Nanoparticles using *Malva sylvestris* in both human liver and breast cancer cell lines. • The data are useful to compare the physicochemical characters, cytotoxic and antibacterial performances of the synthesized nanoparticles and can investigate the reliability of these synthesized nanoparticles in clinical applications. • These data are relevant in developmental biology, especially for the understanding of the antibacterial and anticancer role of synthesized Fe_3_O_4_ nanoparticles using *Malva sylvestris* and their usage in therapeutic procedures for human cancers. So, it may be helpful for biology researchers to find a cancer therapy method. • The synthesized nanoparticles can be applied as an antibacterial, antiseptic, and chemo-preventive agent in cancer treatment.

## 1 Data

**Fig. 1:**
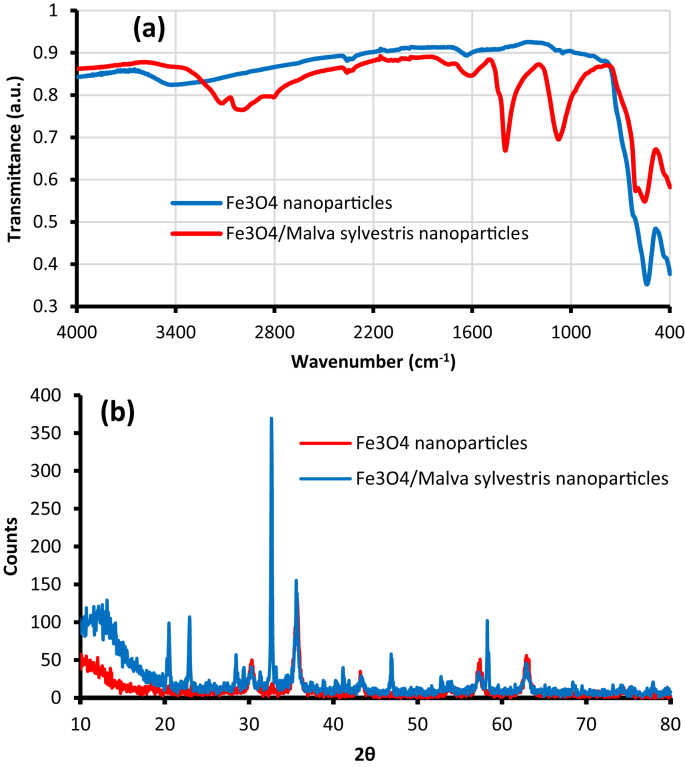
**(a)** FTIR and **(b)** XRD results for the Fe_3_O_4_ and Fe_3_O_4_/*Malva sylvestris* nanoparticles.

**Fig. 2:**
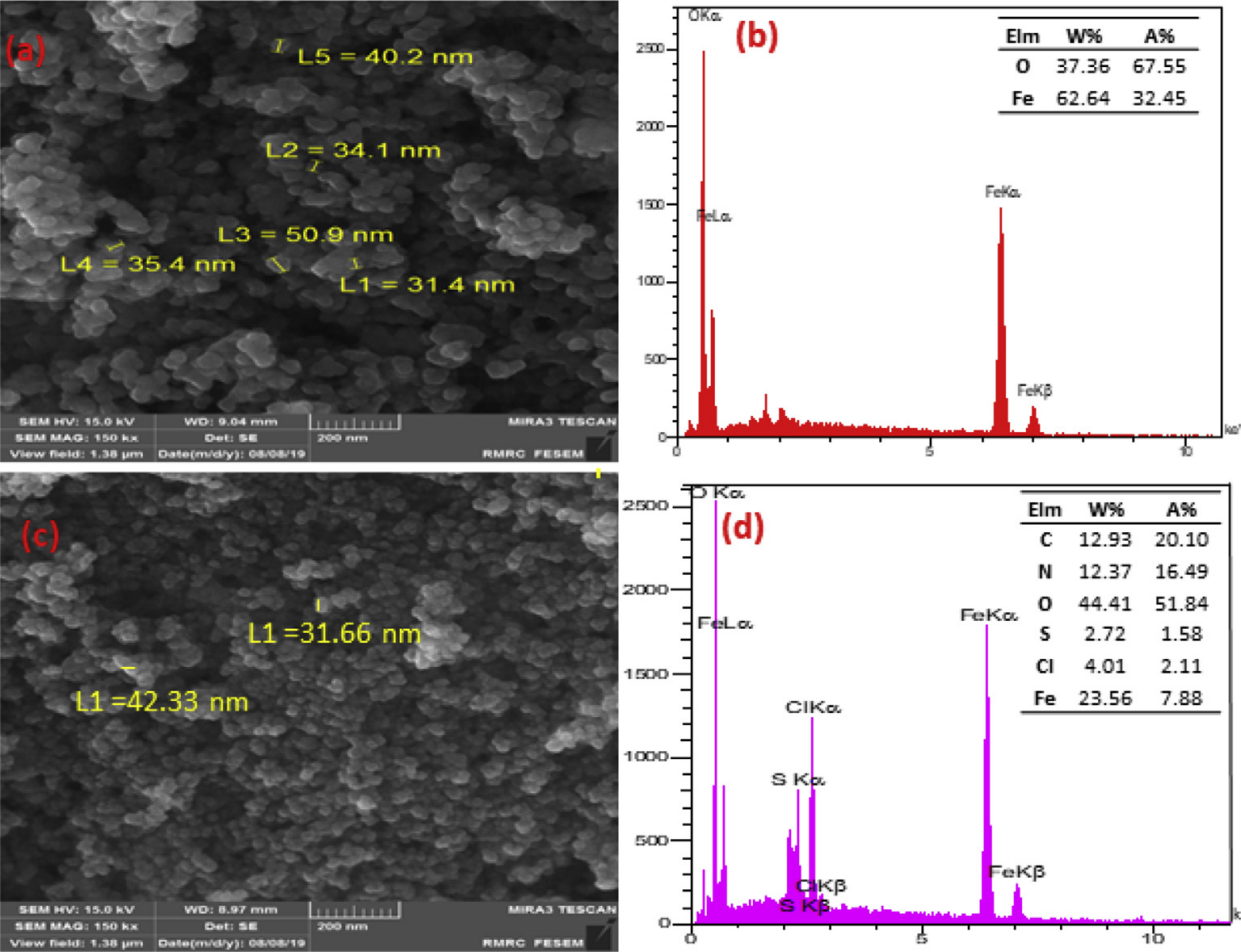
FESEM images and EDAX analysis of **(a,b)** Fe_3_O_4_ nanoparticles and **(c,d)** Fe_3_O_4_/*Malva sylvestris* nanoparticles.

The experimental data on cytotoxic and antibacterial activity of synthesized Fe_3_O_4_ nanoparticles using *Malva sylvestris* are reported in this dataset. To validate the successful synthesis of Fe_3_O_4_ nanoparticles using *Malva sylvestris*, Fourier transform infrared spectroscopy (FTIR) was applied to determine the functional groups in the nanoparticles (Fig. 1a). The X-ray powder diffraction (XRD) analysis is depicted in Fig. 1b. SEM images and EDX analysis of Fe_3_O_4_ nanoparticles and Fe_3_O_4_/*Malva sylvestris* nanoparticles are presented in Fig. 2.

**Fig. 3:**
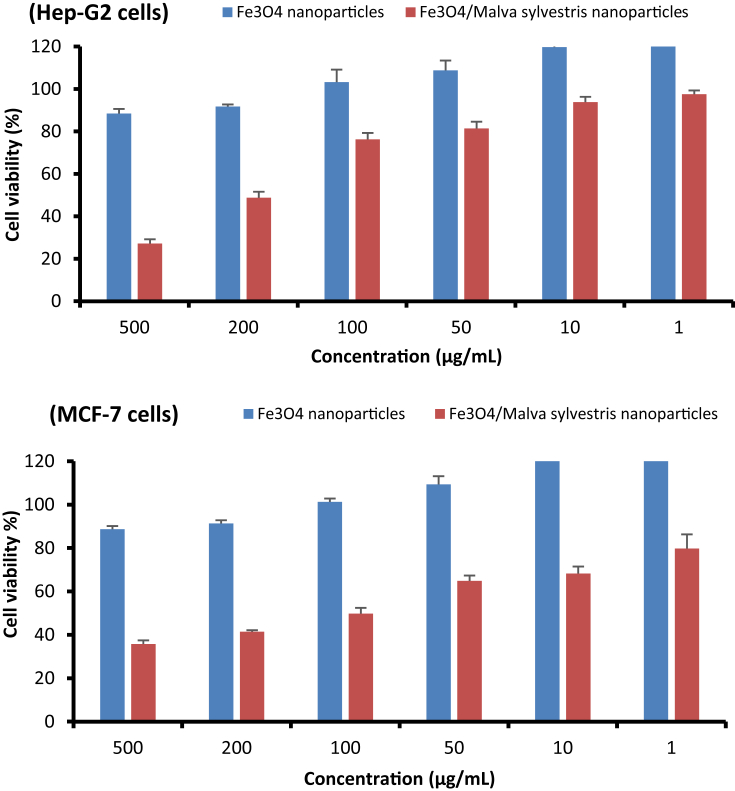
Effect of nanoparticles on cell viability of MTT assay for all tested concentrations on Hep-G2 and MCF-7 cells after 24 h in comparison with control (untreated cell). Each bar represents the mean ± SD (standard deviation) of three independent tests.

**Fig. 4:**
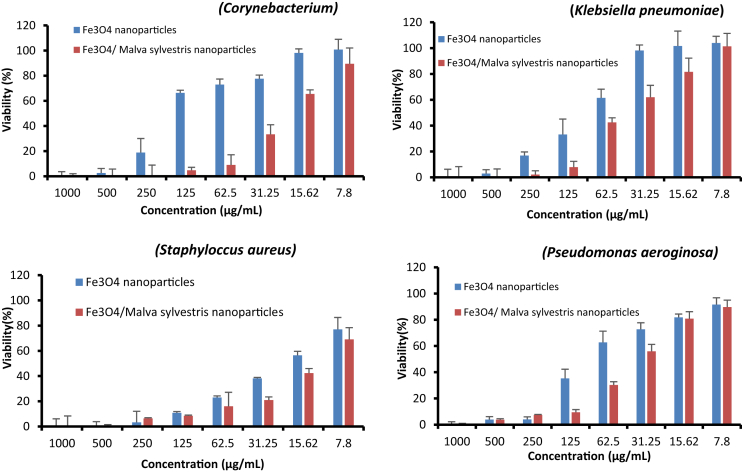
Effect of nanoparticles on the viability percentages of *Corynebacterium*, *Klebsiella pneumoniae*, *Staphylococcus aureus*, *Pseudomonas aeruginosa* in tested concentrations (each bar represents the mean ± SD (standard deviation) of three independent tests).

The cytotoxic effects of synthesized Fe_3_O_4_ nanoparticles using *Malva sylvestris* against Hep-G2 and MCF-7 cell lines have demonstrated in Fig. 3. The inhibitory effect of synthesized Fe_3_O_4_ nanoparticles using *Malva sylvestris* against four bacterial strains was analyzed through microdilution broth assay (see Fig. 4). The performance of nanoparticles against selected microorganisms is presented in Table 1. The raw data source for this dataset is available in the Supplementary section.

**Table 1:** Performance of nanoparticles against selected microorganisms.

Microorganisms	Fe_3_O_4_ nanoparticles (μg/mL)		Fe_3_O_4_/*Malva sylvestris* nanoparticles (μg/mL)	
	MIC	MBC	MIC	MBC
*Staphylococcus aureus*	250	250	125	>125
*Corynebacterium*	500	1000	62.5	125
*Pseudomonas aeruginosa*	250	500	125	>125
*Klebsiella pneumoniae*	500	500	125	>125

## 2 Experimental design, materials, and methods

### 2.1 Preparation of Fe_3_O_4_/Malva sylvestris nanoparticles

All of the materials utilized in this investigation like Ferrous(ІІ) Sulfate Heptahydrate (FeSO_4_.7H_2_O), Iron(III) Chloride Six hydrate (FeCl_3_.6H_2_O) and Ammonia solution were purchased from Merck Co. (Germany). First, 4.75 g FeCl_3_.6H_2_O, 3.89 g FeSO_4_.7H_2_O, and 320 mL deionized water were poured into a round-bottom flask and stirred for 1 h while the temperature was set to 80 °C [1,2]. Then, 0.5 g *Malva sylvestris* was ultrasonically mixed in 120 mL of deionized water for 30 min and then poured into the previous suspension. The obtained suspension was stirred under 80 °C for about 2 h and after that 40 mL NH_3_ was gradually added to the suspension [3]. After filtration, the suspension was washed and the pH scale was set on 7 and finally dried in an oven at 100 °C for 2 h.

### 2.2 Characterization of nanoparticles

The synthesized nanoparticles were characterized using Fourier-transform infrared spectroscopy (FTIR, Tensor ІІ FT-IR spectroscopy Bruker, Germany) in the region of 400–4000 cm^−1^, X-ray diffraction (XRD, Panalytical model X'Pert Pro), scanning electron microscope (SEM, Tescan model Mira III), and EDX (Tescan model S Max detector Mira III).

### 2.3 MTT assay

The cytotoxicity of the synthesized nanoparticles was evaluated on Hep-G2 and MCF-7 cell lines using MTT colorimetric assay. In this method, hydrogen peroxide was considered as the positive control and culture medium was the negative control. Briefly, a certain number of Hep-G2 and MCF-7 cells (10*10^3^) were placed in each well of a sterile 96-well microplate and incubated in a humidified atmosphere of 5% CO_2_, 95% air at 37 °C to reach about 75–90% confluence. Then, 100 μL of the synthesized nanoparticles in a wide range of concentrations was replaced with previous media. Afterward, 25 μL of the MTT 3-(4,5 Dimethylthiazol-2-yl)-2,5-diphenyltetrazolium stock solution (with 4 mg/mL concentration) was transferred into each well and incubated for 4 h in standard condition. The mitochondrial performance of viable cells led to formation of purple formazan crystals, where for dissolving these crystals we applied 100 μL dimethyl sulfoxide (DMSO). In the final step, the absorption of solution was recorded at 570 nm wavelength using a microplate reader (Model 50, Bio-Rad Corp, Hercules, California, USA).

### 2.4 Minimum inhibitory concentrations (MICs) assay

In MICs test, all of the procedures were carried out according to the standards of the Clinical and Laboratory Standards Institute (CLSI) for assessing the antibacterial susceptibility of the synthesized nanoparticles [4]. Briefly, 2-fold serial dilutions of the testing compounds (at a descending concentration from 1000μg/ml to 7.8 μg/mL) and control groups were provided with Brain heart infusion (BHI) in 96-well microplates. Afterward, the microbial concentration was adapted to match the turbidity standard of *0.5 McFarland* (OD600: 0.1–0.2) in a way that the concentration of the compounds was 1000 μg/mL in the first wells. The plates were incubated for 24 h at 37 °C. Later the optical density was measured at 600 nm by a microplate reader (BioTek, Power Wave XS2). This procedure was done in triplicate.

### 2.5 Minimum Bactericidal Concentrations (MBCs) assay

All the selected microorganisms were cultured overnight in BHI, and then stocks with the concentration of 10^5^–10^6^ CFU/mL were prepared for each one. The total of 90 μL of serially diluted concentrations of compounds (from 1000 μg/mL to 7.8 μg/mL) was added to a 96-well micro-plate consisting of 90 μg/mL BHI, then 10 μg/ml of bacteria were added to each cell. Micro-plates were incubated for 24 h at 37 °C. Then, 10 μL of each bacterial suspension was added to a newly prepared BHI and incubated for another 24 hours at 37 °C to exam bactericidal performance of each compound The lowest concentration of compounds that leads no growth of bacteria was regarded as minimum bactericidal concentration (MBC). This procedure was also repeated three times.
