# Supplementary material for: Data on cytotoxic and antibacterial activity of synthesized Fe3O4 nanoparticles using Malva sylvestris
Source: Data Brief. 2019 Dec 9;28:104929. doi: 10.1016/j.dib.2019.104929 (PMC6923292; doi:10.1016/j.dib.2019.104929)
Supplement: Multimedia component 1 [file mmc1.docx]

Data source for

**Data on cytotoxic and antibacterial activity of synthesized Fe_3_O_4_ nanoparticles using *Malva sylvestris***

Seyyed Mojtaba Mousavi ^1,3^, Seyyed Alireza Hashemi ^1,2, *^, Maryam Zarei ^1^, Sonia Bahrani ^1^, Amir Savardashtaki ^4^, [Hossein Esmaeili](https://www.researchgate.net/profile/Hossein_Esmaeili2) ^5^,Chin Wei Lai ^3^, Sargol Mazraedoost ^1^, Mohsen Abassi ^1^, Bahman Ramavandi ^5,6,*^

^1^ Department of Medical Nanotechnology, School of Advanced Medical Sciences and Technologies, Shiraz University of Medical Sciences, Shiraz, Iran

^2^ Department of Mechanical Engineering, Center for Nanofibers and Nanotechnology, National University of Singapore, Singapore

^3^ Nanotechnology & Catalysis Research Center, University of Malaya, Malaysia

^4^ Department of Medical Nanotechnology, School of Advanced Medical Sciences and Technologies, Shiraz University of Medical Sciences, Shiraz, Iran

^5^ Department of Chemical Engineering, Bushehr Branch, Islamic Azad University, Bushehr, Iran

^6^ Department of Environmental Health Engineering, Faculty of Health and Nutrition, Bushehr University of Medical Sciences, Bushehr, Iran

^7^ Systems Environmental Health and Energy Research Center, The Persian Gulf Biomedical Sciences Research Institute, Bushehr University of Medical Sciences, Bushehr, Iran

***Corresponding author:** S. A. Hashemi (Phone: +989398432509; email: sa_hashemi@sums.ac.ir) and B. Ramavandi (Phone: +989363311903; email: [ramavandi_b@yahoo.com](mailto:ramavandi_b@yahoo.com); b.ramavandi@bpums.ac.ir)

**Table S1**. Data source for Fig. 1a.

| Fe_3_O_4_ nanoparticle | |  | Fe_3_O_4_/*Malva syvestris* nanoparticle | |
| --- | --- | --- | --- | --- |
| 399.9059 | 0.37741 |  | 399.924 | 0.58264 |
| 401.3342 | 0.38099 |  | 401.3523 | 0.58496 |
| 402.7624 | 0.38421 |  | 402.7806 | 0.58671 |
| 404.1906 | 0.38739 |  | 404.2089 | 0.58813 |
| 405.6189 | 0.39061 |  | 405.6372 | 0.58942 |
| 407.0471 | 0.39374 |  | 407.0655 | 0.59071 |
| 408.4753 | 0.39663 |  | 408.4938 | 0.59208 |
| 409.9036 | 0.39919 |  | 409.9221 | 0.59359 |
| 411.3318 | 0.40138 |  | 411.3504 | 0.59524 |
| 412.76 | 0.40321 |  | 412.7787 | 0.59694 |
| 414.1883 | 0.40479 |  | 414.207 | 0.59853 |
| 415.6165 | 0.40639 |  | 415.6353 | 0.60001 |
| 417.0447 | 0.40835 |  | 417.0636 | 0.60167 |
| 418.473 | 0.41073 |  | 418.4919 | 0.60387 |
| 419.9012 | 0.41294 |  | 419.9202 | 0.60602 |
| 421.3295 | 0.41436 |  | 421.3485 | 0.60721 |
| 422.7577 | 0.41509 |  | 422.7768 | 0.60769 |
| 424.1859 | 0.41552 |  | 424.2051 | 0.60796 |
| 425.6142 | 0.41586 |  | 425.6334 | 0.60835 |
| 427.0424 | 0.41614 |  | 427.0617 | 0.60893 |
| 428.4706 | 0.41638 |  | 428.49 | 0.60969 |
| 429.8989 | 0.41663 |  | 429.9183 | 0.61053 |
| 431.3271 | 0.41696 |  | 431.3466 | 0.61131 |
| 432.7553 | 0.41742 |  | 432.7749 | 0.61203 |
| 434.1836 | 0.41803 |  | 434.2032 | 0.61287 |
| 435.6118 | 0.41875 |  | 435.6315 | 0.61397 |
| 437.04 | 0.41964 |  | 437.0598 | 0.61521 |
| 438.4683 | 0.42084 |  | 438.4881 | 0.61644 |
| 439.8965 | 0.42255 |  | 439.9164 | 0.6178 |
| 441.3247 | 0.42488 |  | 441.3447 | 0.61956 |
| 442.753 | 0.42772 |  | 442.773 | 0.62175 |
| 444.1812 | 0.43078 |  | 444.2013 | 0.62405 |
| 445.6095 | 0.43378 |  | 445.6296 | 0.62625 |
| 447.0377 | 0.4366 |  | 447.0579 | 0.6284 |
| 448.4659 | 0.43935 |  | 448.4862 | 0.63062 |
| 449.8942 | 0.44216 |  | 449.9145 | 0.63291 |
| 451.3224 | 0.44507 |  | 451.3428 | 0.63534 |
| 452.7506 | 0.44803 |  | 452.7711 | 0.63781 |
| 454.1789 | 0.45106 |  | 454.1994 | 0.64012 |
| 455.6071 | 0.4542 |  | 455.6277 | 0.64237 |
| 457.0353 | 0.45744 |  | 457.056 | 0.64495 |
| 458.4636 | 0.46055 |  | 458.4843 | 0.64784 |
| 459.8918 | 0.46324 |  | 459.9126 | 0.65054 |
| 461.32 | 0.46546 |  | 461.3409 | 0.65299 |
| 462.7483 | 0.46745 |  | 462.7692 | 0.65531 |
| 464.1765 | 0.46941 |  | 464.1975 | 0.65739 |
| 465.6047 | 0.47138 |  | 465.6258 | 0.65904 |
| 467.033 | 0.47316 |  | 467.0541 | 0.66029 |
| 468.4612 | 0.47461 |  | 468.4824 | 0.66132 |
| 469.8895 | 0.47573 |  | 469.9107 | 0.66237 |
| 471.3177 | 0.47667 |  | 471.339 | 0.66366 |
| 472.7459 | 0.47752 |  | 472.7673 | 0.66521 |
| 474.1742 | 0.47829 |  | 474.1956 | 0.66672 |
| 475.6024 | 0.47893 |  | 475.6239 | 0.66795 |
| 477.0306 | 0.47946 |  | 477.0522 | 0.66884 |
| 478.4589 | 0.48001 |  | 478.4805 | 0.66947 |
| 479.8871 | 0.4807 |  | 479.9088 | 0.67003 |
| 481.3153 | 0.48159 |  | 481.3371 | 0.67067 |
| 482.7436 | 0.48257 |  | 482.7654 | 0.6713 |
| 484.1718 | 0.48347 |  | 484.1937 | 0.67169 |
| 485.6 | 0.48405 |  | 485.622 | 0.67171 |
| 487.0283 | 0.48421 |  | 487.0503 | 0.67149 |
| 488.4565 | 0.48388 |  | 488.4786 | 0.67118 |
| 489.8848 | 0.48303 |  | 489.9069 | 0.67074 |
| 491.313 | 0.48162 |  | 491.3352 | 0.67 |
| 492.7412 | 0.47962 |  | 492.7635 | 0.66883 |
| 494.1695 | 0.47709 |  | 494.1918 | 0.66733 |
| 495.5977 | 0.47411 |  | 495.6201 | 0.66561 |
| 497.0259 | 0.47074 |  | 497.0484 | 0.66367 |
| 498.4542 | 0.46699 |  | 498.4767 | 0.66151 |
| 499.8824 | 0.46281 |  | 499.905 | 0.65907 |
| 501.3106 | 0.45821 |  | 501.3333 | 0.65628 |
| 502.7389 | 0.45329 |  | 502.7616 | 0.65322 |
| 504.1671 | 0.44825 |  | 504.1899 | 0.65002 |
| 505.5953 | 0.44317 |  | 505.6182 | 0.64678 |
| 507.0236 | 0.43811 |  | 507.0465 | 0.64357 |
| 508.4518 | 0.43309 |  | 508.4748 | 0.64049 |
| 509.88 | 0.42817 |  | 509.9031 | 0.63747 |
| 511.3083 | 0.42337 |  | 511.3314 | 0.63428 |
| 512.7365 | 0.41862 |  | 512.7597 | 0.63085 |
| 514.1648 | 0.41383 |  | 514.188 | 0.62735 |
| 515.593 | 0.40891 |  | 515.6163 | 0.62376 |
| 517.0212 | 0.4038 |  | 517.0446 | 0.61988 |
| 518.4495 | 0.39849 |  | 518.4729 | 0.61568 |
| 519.8777 | 0.39314 |  | 519.9012 | 0.61144 |
| 521.3059 | 0.38795 |  | 521.3295 | 0.60749 |
| 522.7342 | 0.38301 |  | 522.7578 | 0.60377 |
| 524.1624 | 0.37829 |  | 524.1861 | 0.59995 |
| 525.5906 | 0.37382 |  | 525.6144 | 0.5958 |
| 527.0189 | 0.36972 |  | 527.0427 | 0.59159 |
| 528.4471 | 0.36608 |  | 528.471 | 0.58776 |
| 529.8753 | 0.36293 |  | 529.8993 | 0.58427 |
| 531.3036 | 0.36021 |  | 531.3276 | 0.58088 |
| 532.7318 | 0.35792 |  | 532.7559 | 0.57751 |
| 534.16 | 0.35604 |  | 534.1842 | 0.57422 |
| 535.5883 | 0.35454 |  | 535.6125 | 0.57102 |
| 537.0165 | 0.35343 |  | 537.0408 | 0.56792 |
| 538.4448 | 0.35276 |  | 538.4691 | 0.56491 |
| 539.873 | 0.35256 |  | 539.8974 | 0.56204 |
| 541.3012 | 0.35286 |  | 541.3257 | 0.55944 |
| 542.7295 | 0.35362 |  | 542.754 | 0.55722 |
| 544.1577 | 0.35486 |  | 544.1823 | 0.55534 |
| 545.5859 | 0.35655 |  | 545.6106 | 0.55361 |
| 547.0142 | 0.35869 |  | 547.0389 | 0.55198 |
| 548.4424 | 0.3613 |  | 548.4672 | 0.55058 |
| 549.8706 | 0.3644 |  | 549.8955 | 0.54957 |
| 551.2989 | 0.36792 |  | 551.3238 | 0.54901 |
| 552.7271 | 0.37173 |  | 552.7521 | 0.54874 |
| 554.1553 | 0.37578 |  | 554.1804 | 0.54864 |
| 555.5836 | 0.38005 |  | 555.6087 | 0.54873 |
| 557.0118 | 0.38452 |  | 557.037 | 0.5491 |
| 558.4401 | 0.38913 |  | 558.4653 | 0.54977 |
| 559.8683 | 0.39381 |  | 559.8936 | 0.55071 |
| 561.2965 | 0.39844 |  | 561.3219 | 0.55183 |
| 562.7248 | 0.40287 |  | 562.7502 | 0.55306 |
| 564.153 | 0.40699 |  | 564.1785 | 0.55443 |
| 565.5812 | 0.41082 |  | 565.6068 | 0.55595 |
| 567.0095 | 0.41444 |  | 567.0351 | 0.55752 |
| 568.4377 | 0.41786 |  | 568.4634 | 0.55905 |
| 569.8659 | 0.42105 |  | 569.8917 | 0.56046 |
| 571.2942 | 0.42401 |  | 571.32 | 0.56173 |
| 572.7224 | 0.42683 |  | 572.7483 | 0.56287 |
| 574.1506 | 0.42962 |  | 574.1766 | 0.56399 |
| 575.5789 | 0.43245 |  | 575.6049 | 0.56516 |
| 577.0071 | 0.43534 |  | 577.0332 | 0.5664 |
| 578.4353 | 0.4383 |  | 578.4615 | 0.56769 |
| 579.8636 | 0.44135 |  | 579.8898 | 0.56901 |
| 581.2918 | 0.44454 |  | 581.3181 | 0.57031 |
| 582.7201 | 0.44789 |  | 582.7464 | 0.57156 |
| 584.1483 | 0.45136 |  | 584.1747 | 0.57283 |
| 585.5765 | 0.45487 |  | 585.603 | 0.57414 |
| 587.0048 | 0.45835 |  | 587.0313 | 0.57545 |
| 588.433 | 0.46184 |  | 588.4596 | 0.57667 |
| 589.8612 | 0.4654 |  | 589.8879 | 0.57776 |
| 591.2895 | 0.46903 |  | 591.3162 | 0.57872 |
| 592.7177 | 0.47267 |  | 592.7445 | 0.57947 |
| 594.1459 | 0.47619 |  | 594.1728 | 0.57998 |
| 595.5742 | 0.47953 |  | 595.6011 | 0.58028 |
| 597.0024 | 0.4827 |  | 597.0294 | 0.58035 |
| 598.4306 | 0.4858 |  | 598.4577 | 0.58014 |
| 599.8589 | 0.48886 |  | 599.886 | 0.57967 |
| 601.2871 | 0.49183 |  | 601.3143 | 0.579 |
| 602.7153 | 0.49462 |  | 602.7426 | 0.57812 |
| 604.1436 | 0.49721 |  | 604.1709 | 0.57698 |
| 605.5718 | 0.49966 |  | 605.5992 | 0.57563 |
| 607.0001 | 0.50197 |  | 607.0275 | 0.5743 |
| 608.4283 | 0.50413 |  | 608.4558 | 0.57351 |
| 609.8565 | 0.50608 |  | 609.8841 | 0.57394 |
| 611.2848 | 0.50779 |  | 611.3124 | 0.57623 |
| 612.713 | 0.50926 |  | 612.7407 | 0.58074 |
| 614.1412 | 0.51047 |  | 614.169 | 0.58741 |
| 615.5695 | 0.51144 |  | 615.5973 | 0.59578 |
| 616.9977 | 0.5122 |  | 617.0256 | 0.6052 |
| 618.4259 | 0.51283 |  | 618.4539 | 0.61497 |
| 619.8542 | 0.51344 |  | 619.8822 | 0.62462 |
| 621.2824 | 0.51416 |  | 621.3105 | 0.63411 |
| 622.7106 | 0.51516 |  | 622.7388 | 0.64368 |
| 624.1389 | 0.51659 |  | 624.1671 | 0.65342 |
| 625.5671 | 0.51851 |  | 625.5954 | 0.66305 |
| 626.9954 | 0.52099 |  | 627.0237 | 0.67196 |
| 628.4236 | 0.52403 |  | 628.452 | 0.67969 |
| 629.8518 | 0.52762 |  | 629.8803 | 0.68619 |
| 631.2801 | 0.53173 |  | 631.3086 | 0.69168 |
| 632.7083 | 0.53633 |  | 632.7369 | 0.69637 |
| 634.1365 | 0.54135 |  | 634.1652 | 0.70045 |
| 635.5648 | 0.54666 |  | 635.5935 | 0.70409 |
| 636.993 | 0.55214 |  | 637.0218 | 0.70741 |
| 638.4212 | 0.55775 |  | 638.4501 | 0.71046 |
| 639.8495 | 0.56348 |  | 639.8784 | 0.71335 |
| 641.2777 | 0.56932 |  | 641.3067 | 0.71614 |
| 642.7059 | 0.57518 |  | 642.735 | 0.71892 |
| 644.1342 | 0.58101 |  | 644.1633 | 0.72175 |
| 645.5624 | 0.58677 |  | 645.5916 | 0.72466 |
| 646.9906 | 0.59243 |  | 647.0199 | 0.72758 |
| 648.4189 | 0.598 |  | 648.4482 | 0.73042 |
| 649.8471 | 0.60341 |  | 649.8765 | 0.73311 |
| 651.2754 | 0.60859 |  | 651.3048 | 0.73561 |
| 652.7036 | 0.61347 |  | 652.7331 | 0.73798 |
| 654.1318 | 0.61805 |  | 654.1614 | 0.74037 |
| 655.5601 | 0.62238 |  | 655.5897 | 0.74288 |
| 656.9883 | 0.62647 |  | 657.018 | 0.74556 |
| 658.4165 | 0.63038 |  | 658.4463 | 0.74834 |
| 659.8448 | 0.63409 |  | 659.8746 | 0.75105 |
| 661.273 | 0.6375 |  | 661.3029 | 0.75356 |
| 662.7012 | 0.64047 |  | 662.7312 | 0.75577 |
| 664.1295 | 0.64302 |  | 664.1595 | 0.75749 |
| 665.5577 | 0.6455 |  | 665.5878 | 0.7587 |
| 666.9859 | 0.64845 |  | 667.0161 | 0.75976 |
| 668.4142 | 0.65209 |  | 668.4444 | 0.76139 |
| 669.8424 | 0.65568 |  | 669.8727 | 0.76355 |
| 671.2706 | 0.65851 |  | 671.301 | 0.76548 |
| 672.6989 | 0.66066 |  | 672.7293 | 0.76707 |
| 674.1271 | 0.6626 |  | 674.1576 | 0.76852 |
| 675.5554 | 0.66463 |  | 675.5859 | 0.76992 |
| 676.9836 | 0.66679 |  | 677.0142 | 0.7713 |
| 678.4118 | 0.66903 |  | 678.4425 | 0.77268 |
| 679.8401 | 0.67129 |  | 679.8708 | 0.77406 |
| 681.2683 | 0.67356 |  | 681.2991 | 0.7754 |
| 682.6965 | 0.67581 |  | 682.7274 | 0.77675 |
| 684.1248 | 0.67805 |  | 684.1557 | 0.77811 |
| 685.553 | 0.68035 |  | 685.584 | 0.77951 |
| 686.9812 | 0.68281 |  | 687.0123 | 0.78095 |
| 688.4095 | 0.68545 |  | 688.4406 | 0.78246 |
| 689.8377 | 0.68829 |  | 689.8689 | 0.78407 |
| 691.2659 | 0.69128 |  | 691.2972 | 0.78572 |
| 692.6942 | 0.69441 |  | 692.7255 | 0.78736 |
| 694.1224 | 0.69769 |  | 694.1538 | 0.789 |
| 695.5507 | 0.70117 |  | 695.5821 | 0.79069 |
| 696.9789 | 0.70484 |  | 697.0104 | 0.79243 |
| 698.4071 | 0.70867 |  | 698.4387 | 0.79423 |
| 699.8354 | 0.71258 |  | 699.867 | 0.79607 |
| 701.2636 | 0.71653 |  | 701.2953 | 0.7979 |
| 702.6918 | 0.72048 |  | 702.7236 | 0.79965 |
| 704.1201 | 0.72439 |  | 704.1519 | 0.80133 |
| 705.5483 | 0.72818 |  | 705.5802 | 0.80298 |
| 706.9765 | 0.73182 |  | 707.0085 | 0.80458 |
| 708.4048 | 0.73523 |  | 708.4368 | 0.80611 |
| 709.833 | 0.7384 |  | 709.8651 | 0.80758 |
| 711.2612 | 0.7413 |  | 711.2934 | 0.80902 |
| 712.6895 | 0.74396 |  | 712.7217 | 0.81047 |
| 714.1177 | 0.74642 |  | 714.15 | 0.81193 |
| 715.5459 | 0.74872 |  | 715.5783 | 0.81336 |
| 716.9742 | 0.75094 |  | 717.0066 | 0.81477 |
| 718.4024 | 0.7532 |  | 718.4349 | 0.8162 |
| 719.8307 | 0.75559 |  | 719.8632 | 0.81776 |
| 721.2589 | 0.75816 |  | 721.2915 | 0.81956 |
| 722.6871 | 0.7609 |  | 722.7198 | 0.82153 |
| 724.1154 | 0.76383 |  | 724.1481 | 0.82363 |
| 725.5436 | 0.76699 |  | 725.5764 | 0.82582 |
| 726.9718 | 0.77041 |  | 727.0047 | 0.82812 |
| 728.4001 | 0.77416 |  | 728.433 | 0.83048 |
| 729.8283 | 0.77827 |  | 729.8613 | 0.83288 |
| 731.2565 | 0.78273 |  | 731.2896 | 0.83529 |
| 732.6848 | 0.78747 |  | 732.7179 | 0.83772 |
| 734.113 | 0.79246 |  | 734.1462 | 0.84023 |
| 735.5412 | 0.79764 |  | 735.5745 | 0.84281 |
| 736.9695 | 0.80299 |  | 737.0028 | 0.84538 |
| 738.3977 | 0.80847 |  | 738.4311 | 0.84788 |
| 739.8259 | 0.81399 |  | 739.8594 | 0.85026 |
| 741.2542 | 0.81946 |  | 741.2877 | 0.85251 |
| 742.6824 | 0.82473 |  | 742.716 | 0.85463 |
| 744.1107 | 0.82969 |  | 744.1443 | 0.85662 |
| 745.5389 | 0.83428 |  | 745.5726 | 0.85846 |
| 746.9671 | 0.83852 |  | 747.0009 | 0.86015 |
| 748.3954 | 0.84244 |  | 748.4292 | 0.86164 |
| 749.8236 | 0.84606 |  | 749.8575 | 0.86291 |
| 751.2518 | 0.8494 |  | 751.2858 | 0.86398 |
| 752.6801 | 0.85246 |  | 752.7141 | 0.86489 |
| 754.1083 | 0.85524 |  | 754.1424 | 0.86568 |
| 755.5365 | 0.85775 |  | 755.5707 | 0.86635 |
| 756.9648 | 0.85999 |  | 756.999 | 0.86691 |
| 758.393 | 0.86198 |  | 758.4273 | 0.8674 |
| 759.8212 | 0.86375 |  | 759.8556 | 0.86783 |
| 761.2495 | 0.86535 |  | 761.2839 | 0.86824 |
| 762.6777 | 0.86681 |  | 762.7122 | 0.86864 |
| 764.106 | 0.86817 |  | 764.1405 | 0.86895 |
| 765.5342 | 0.86945 |  | 765.5688 | 0.86917 |
| 766.9624 | 0.87066 |  | 766.9971 | 0.86934 |
| 768.3907 | 0.87182 |  | 768.4254 | 0.86951 |
| 769.8189 | 0.87291 |  | 769.8537 | 0.86968 |
| 771.2471 | 0.8739 |  | 771.282 | 0.86985 |
| 772.6754 | 0.87479 |  | 772.7103 | 0.87 |
| 774.1036 | 0.8756 |  | 774.1386 | 0.8701 |
| 775.5318 | 0.87634 |  | 775.5669 | 0.87015 |
| 776.9601 | 0.87704 |  | 776.9952 | 0.87015 |
| 778.3883 | 0.87768 |  | 778.4235 | 0.87014 |
| 779.8165 | 0.87827 |  | 779.8518 | 0.87012 |
| 781.2448 | 0.8788 |  | 781.2801 | 0.87012 |
| 782.673 | 0.87927 |  | 782.7084 | 0.87016 |
| 784.1012 | 0.87969 |  | 784.1367 | 0.87024 |
| 785.5295 | 0.88004 |  | 785.565 | 0.8703 |
| 786.9577 | 0.88033 |  | 786.9933 | 0.87035 |
| 788.386 | 0.88057 |  | 788.4216 | 0.87043 |
| 789.8142 | 0.88076 |  | 789.8499 | 0.87052 |
| 791.2424 | 0.88091 |  | 791.2782 | 0.87057 |
| 792.6707 | 0.88101 |  | 792.7065 | 0.87057 |
| 794.0989 | 0.88107 |  | 794.1348 | 0.87053 |
| 795.5271 | 0.88112 |  | 795.5631 | 0.87045 |
| 796.9554 | 0.88115 |  | 796.9914 | 0.87033 |
| 798.3836 | 0.88114 |  | 798.4197 | 0.87014 |
| 799.8118 | 0.88109 |  | 799.848 | 0.86992 |
| 801.2401 | 0.88103 |  | 801.2763 | 0.8697 |
| 802.6683 | 0.88097 |  | 802.7046 | 0.86946 |
| 804.0965 | 0.88093 |  | 804.1329 | 0.86916 |
| 805.5248 | 0.88089 |  | 805.5612 | 0.86884 |
| 806.953 | 0.88083 |  | 806.9895 | 0.8685 |
| 808.3812 | 0.88076 |  | 808.4178 | 0.86817 |
| 809.8095 | 0.8807 |  | 809.8461 | 0.86787 |
| 811.2377 | 0.88066 |  | 811.2744 | 0.86762 |
| 812.666 | 0.88065 |  | 812.7027 | 0.86745 |
| 814.0942 | 0.88069 |  | 814.131 | 0.86732 |
| 815.5224 | 0.88078 |  | 815.5593 | 0.86722 |
| 816.9507 | 0.88094 |  | 816.9876 | 0.86715 |
| 818.3789 | 0.88118 |  | 818.4158 | 0.86711 |
| 819.8071 | 0.8815 |  | 819.8441 | 0.86708 |
| 821.2354 | 0.8819 |  | 821.2724 | 0.86708 |
| 822.6636 | 0.88236 |  | 822.7007 | 0.86708 |
| 824.0918 | 0.88283 |  | 824.129 | 0.8671 |
| 825.5201 | 0.8833 |  | 825.5573 | 0.86713 |
| 826.9483 | 0.88372 |  | 826.9856 | 0.86721 |
| 828.3765 | 0.8841 |  | 828.4139 | 0.86733 |
| 829.8048 | 0.88449 |  | 829.8422 | 0.86741 |
| 831.233 | 0.88487 |  | 831.2705 | 0.86741 |
| 832.6612 | 0.88523 |  | 832.6988 | 0.86733 |
| 834.0895 | 0.88556 |  | 834.1271 | 0.8672 |
| 835.5177 | 0.88587 |  | 835.5554 | 0.86703 |
| 836.946 | 0.88618 |  | 836.9837 | 0.86686 |
| 838.3742 | 0.88653 |  | 838.412 | 0.86671 |
| 839.8024 | 0.88692 |  | 839.8403 | 0.86658 |
| 841.2307 | 0.88733 |  | 841.2686 | 0.86642 |
| 842.6589 | 0.88774 |  | 842.6969 | 0.86623 |
| 844.0871 | 0.88814 |  | 844.1252 | 0.86601 |
| 845.5154 | 0.88854 |  | 845.5535 | 0.86576 |
| 846.9436 | 0.88894 |  | 846.9818 | 0.8655 |
| 848.3718 | 0.88935 |  | 848.4101 | 0.86529 |
| 849.8001 | 0.88975 |  | 849.8384 | 0.86512 |
| 851.2283 | 0.89014 |  | 851.2667 | 0.86491 |
| 852.6565 | 0.89052 |  | 852.695 | 0.86455 |
| 854.0848 | 0.89088 |  | 854.1233 | 0.86403 |
| 855.513 | 0.89123 |  | 855.5516 | 0.86341 |
| 856.9413 | 0.89154 |  | 856.9799 | 0.86279 |
| 858.3695 | 0.89182 |  | 858.4082 | 0.86221 |
| 859.7977 | 0.89206 |  | 859.8365 | 0.86168 |
| 861.226 | 0.89226 |  | 861.2648 | 0.86119 |
| 862.6542 | 0.89244 |  | 862.6931 | 0.86072 |
| 864.0824 | 0.89259 |  | 864.1214 | 0.86031 |
| 865.5107 | 0.89275 |  | 865.5497 | 0.85999 |
| 866.9389 | 0.89289 |  | 866.978 | 0.85976 |
| 868.3671 | 0.89302 |  | 868.4063 | 0.85962 |
| 869.7954 | 0.89313 |  | 869.8346 | 0.85951 |
| 871.2236 | 0.89318 |  | 871.2629 | 0.85943 |
| 872.6518 | 0.89318 |  | 872.6912 | 0.85938 |
| 874.0801 | 0.89312 |  | 874.1195 | 0.8593 |
| 875.5083 | 0.89303 |  | 875.5478 | 0.8591 |
| 876.9365 | 0.8929 |  | 876.9761 | 0.85876 |
| 878.3648 | 0.89278 |  | 878.4044 | 0.85835 |
| 879.793 | 0.89269 |  | 879.8327 | 0.85796 |
| 881.2213 | 0.89266 |  | 881.261 | 0.85763 |
| 882.6495 | 0.89269 |  | 882.6893 | 0.85734 |
| 884.0777 | 0.89278 |  | 884.1176 | 0.85707 |
| 885.506 | 0.89289 |  | 885.5459 | 0.85675 |
| 886.9342 | 0.89302 |  | 886.9742 | 0.85634 |
| 888.3624 | 0.89313 |  | 888.4025 | 0.85581 |
| 889.7907 | 0.89322 |  | 889.8308 | 0.85516 |
| 891.2189 | 0.8933 |  | 891.2591 | 0.85447 |
| 892.6471 | 0.8934 |  | 892.6874 | 0.85386 |
| 894.0754 | 0.89352 |  | 894.1157 | 0.85339 |
| 895.5036 | 0.89366 |  | 895.544 | 0.85306 |
| 896.9318 | 0.89382 |  | 896.9723 | 0.85277 |
| 898.3601 | 0.894 |  | 898.4006 | 0.85249 |
| 899.7883 | 0.89418 |  | 899.8289 | 0.85221 |
| 901.2165 | 0.89437 |  | 901.2572 | 0.85195 |
| 902.6448 | 0.89457 |  | 902.6855 | 0.85171 |
| 904.073 | 0.89478 |  | 904.1138 | 0.8515 |
| 905.5013 | 0.895 |  | 905.5421 | 0.85131 |
| 906.9295 | 0.89522 |  | 906.9704 | 0.85111 |
| 908.3577 | 0.89543 |  | 908.3987 | 0.85084 |
| 909.786 | 0.89564 |  | 909.827 | 0.85047 |
| 911.2142 | 0.89582 |  | 911.2553 | 0.85002 |
| 912.6424 | 0.89598 |  | 912.6836 | 0.84951 |
| 914.0707 | 0.89611 |  | 914.1119 | 0.84894 |
| 915.4989 | 0.89623 |  | 915.5402 | 0.84834 |
| 916.9271 | 0.89634 |  | 916.9685 | 0.84771 |
| 918.3554 | 0.89647 |  | 918.3968 | 0.84705 |
| 919.7836 | 0.8966 |  | 919.8251 | 0.84633 |
| 921.2118 | 0.89673 |  | 921.2534 | 0.8456 |
| 922.6401 | 0.89686 |  | 922.6817 | 0.84486 |
| 924.0683 | 0.89698 |  | 924.11 | 0.84412 |
| 925.4966 | 0.8971 |  | 925.5383 | 0.84335 |
| 926.9248 | 0.8972 |  | 926.9666 | 0.8425 |
| 928.353 | 0.89729 |  | 928.3949 | 0.84159 |
| 929.7813 | 0.89735 |  | 929.8232 | 0.84069 |
| 931.2095 | 0.89739 |  | 931.2515 | 0.83987 |
| 932.6377 | 0.89744 |  | 932.6798 | 0.83915 |
| 934.066 | 0.89749 |  | 934.1081 | 0.83851 |
| 935.4942 | 0.89756 |  | 935.5364 | 0.83786 |
| 936.9224 | 0.89764 |  | 936.9647 | 0.83717 |
| 938.3507 | 0.8977 |  | 938.393 | 0.83643 |
| 939.7789 | 0.89776 |  | 939.8213 | 0.83563 |
| 941.2071 | 0.89783 |  | 941.2496 | 0.83475 |
| 942.6354 | 0.89793 |  | 942.6779 | 0.83378 |
| 944.0636 | 0.89805 |  | 944.1062 | 0.83275 |
| 945.4918 | 0.8982 |  | 945.5345 | 0.83168 |
| 946.9201 | 0.89835 |  | 946.9628 | 0.83063 |
| 948.3483 | 0.89848 |  | 948.3911 | 0.82961 |
| 949.7766 | 0.89858 |  | 949.8194 | 0.82862 |
| 951.2048 | 0.89868 |  | 951.2477 | 0.82766 |
| 952.633 | 0.89877 |  | 952.676 | 0.82671 |
| 954.0613 | 0.89888 |  | 954.1043 | 0.82576 |
| 955.4895 | 0.89898 |  | 955.5326 | 0.82483 |
| 956.9177 | 0.89909 |  | 956.9609 | 0.82392 |
| 958.346 | 0.89919 |  | 958.3892 | 0.82303 |
| 959.7742 | 0.89927 |  | 959.8175 | 0.82214 |
| 961.2024 | 0.89934 |  | 961.2458 | 0.82123 |
| 962.6307 | 0.89942 |  | 962.6741 | 0.82028 |
| 964.0589 | 0.89949 |  | 964.1024 | 0.81925 |
| 965.4871 | 0.89956 |  | 965.5307 | 0.81808 |
| 966.9154 | 0.89964 |  | 966.959 | 0.81677 |
| 968.3436 | 0.89972 |  | 968.3873 | 0.81533 |
| 969.7718 | 0.89981 |  | 969.8156 | 0.81376 |
| 971.2001 | 0.89992 |  | 971.2439 | 0.81215 |
| 972.6283 | 0.90002 |  | 972.6722 | 0.81074 |
| 974.0566 | 0.90011 |  | 974.1005 | 0.80985 |
| 975.4848 | 0.90019 |  | 975.5288 | 0.80948 |
| 976.913 | 0.90026 |  | 976.9571 | 0.80927 |
| 978.3413 | 0.90033 |  | 978.3854 | 0.80876 |
| 979.7695 | 0.90042 |  | 979.8137 | 0.80793 |
| 981.1977 | 0.90054 |  | 981.242 | 0.80708 |
| 982.626 | 0.90072 |  | 982.6703 | 0.80646 |
| 984.0542 | 0.90095 |  | 984.0986 | 0.80599 |
| 985.4824 | 0.90122 |  | 985.5269 | 0.80541 |
| 986.9107 | 0.90152 |  | 986.9552 | 0.8046 |
| 988.3389 | 0.90182 |  | 988.3835 | 0.80357 |
| 989.7671 | 0.90211 |  | 989.8118 | 0.80243 |
| 991.1954 | 0.90239 |  | 991.2401 | 0.80122 |
| 992.6236 | 0.90267 |  | 992.6684 | 0.79997 |
| 994.0519 | 0.90295 |  | 994.0967 | 0.79868 |
| 995.4801 | 0.90321 |  | 995.525 | 0.79734 |
| 996.9083 | 0.90346 |  | 996.9533 | 0.79589 |
| 998.3366 | 0.90369 |  | 998.3816 | 0.79435 |
| 999.7648 | 0.90389 |  | 999.8099 | 0.7927 |
| 1001.193 | 0.90406 |  | 1001.238 | 0.79097 |
| 1002.621 | 0.9042 |  | 1002.667 | 0.78916 |
| 1004.049 | 0.90433 |  | 1004.095 | 0.78727 |
| 1005.478 | 0.90444 |  | 1005.523 | 0.78534 |
| 1006.906 | 0.90454 |  | 1006.951 | 0.78339 |
| 1008.334 | 0.90461 |  | 1008.38 | 0.78145 |
| 1009.762 | 0.90465 |  | 1009.808 | 0.77953 |
| 1011.191 | 0.90464 |  | 1011.236 | 0.77764 |
| 1012.619 | 0.9046 |  | 1012.665 | 0.77576 |
| 1014.047 | 0.90454 |  | 1014.093 | 0.77389 |
| 1015.475 | 0.90445 |  | 1015.521 | 0.77202 |
| 1016.904 | 0.90435 |  | 1016.95 | 0.77016 |
| 1018.332 | 0.90423 |  | 1018.378 | 0.7683 |
| 1019.76 | 0.90411 |  | 1019.806 | 0.76645 |
| 1021.188 | 0.904 |  | 1021.234 | 0.76461 |
| 1022.617 | 0.90389 |  | 1022.663 | 0.76273 |
| 1024.045 | 0.90378 |  | 1024.091 | 0.7607 |
| 1025.473 | 0.90367 |  | 1025.519 | 0.7585 |
| 1026.901 | 0.90358 |  | 1026.948 | 0.75629 |
| 1028.33 | 0.90351 |  | 1028.376 | 0.75428 |
| 1029.758 | 0.90345 |  | 1029.804 | 0.75242 |
| 1031.186 | 0.90338 |  | 1031.233 | 0.75045 |
| 1032.614 | 0.90328 |  | 1032.661 | 0.74819 |
| 1034.042 | 0.90313 |  | 1034.089 | 0.74565 |
| 1035.471 | 0.90297 |  | 1035.517 | 0.74298 |
| 1036.899 | 0.90279 |  | 1036.946 | 0.74035 |
| 1038.327 | 0.9026 |  | 1038.374 | 0.73781 |
| 1039.755 | 0.90237 |  | 1039.802 | 0.73533 |
| 1041.184 | 0.90209 |  | 1041.231 | 0.73285 |
| 1042.612 | 0.90173 |  | 1042.659 | 0.73035 |
| 1044.04 | 0.90127 |  | 1044.087 | 0.72791 |
| 1045.468 | 0.90074 |  | 1045.516 | 0.72561 |
| 1046.897 | 0.9002 |  | 1046.944 | 0.72345 |
| 1048.325 | 0.89971 |  | 1048.372 | 0.7214 |
| 1049.753 | 0.89939 |  | 1049.8 | 0.71944 |
| 1051.181 | 0.89931 |  | 1051.229 | 0.71756 |
| 1052.61 | 0.89955 |  | 1052.657 | 0.71581 |
| 1054.038 | 0.9001 |  | 1054.085 | 0.71415 |
| 1055.466 | 0.9009 |  | 1055.514 | 0.71257 |
| 1056.894 | 0.90183 |  | 1056.942 | 0.71108 |
| 1058.322 | 0.90277 |  | 1058.37 | 0.70974 |
| 1059.751 | 0.90361 |  | 1059.799 | 0.70847 |
| 1061.179 | 0.90433 |  | 1061.227 | 0.70702 |
| 1062.607 | 0.90491 |  | 1062.655 | 0.70521 |
| 1064.035 | 0.90538 |  | 1064.083 | 0.70319 |
| 1065.464 | 0.90576 |  | 1065.512 | 0.70134 |
| 1066.892 | 0.90606 |  | 1066.94 | 0.69998 |
| 1068.32 | 0.90629 |  | 1068.368 | 0.69908 |
| 1069.748 | 0.90643 |  | 1069.797 | 0.69829 |
| 1071.177 | 0.90647 |  | 1071.225 | 0.6974 |
| 1072.605 | 0.90645 |  | 1072.653 | 0.69649 |
| 1074.033 | 0.9064 |  | 1074.082 | 0.69577 |
| 1075.461 | 0.90636 |  | 1075.51 | 0.69541 |
| 1076.89 | 0.90636 |  | 1076.938 | 0.69542 |
| 1078.318 | 0.90637 |  | 1078.366 | 0.69574 |
| 1079.746 | 0.90639 |  | 1079.795 | 0.69631 |
| 1081.174 | 0.90638 |  | 1081.223 | 0.69712 |
| 1082.602 | 0.90634 |  | 1082.651 | 0.69816 |
| 1084.031 | 0.90626 |  | 1084.08 | 0.69944 |
| 1085.459 | 0.90617 |  | 1085.508 | 0.70093 |
| 1086.887 | 0.90606 |  | 1086.936 | 0.70256 |
| 1088.315 | 0.90598 |  | 1088.365 | 0.70432 |
| 1089.744 | 0.90594 |  | 1089.793 | 0.70623 |
| 1091.172 | 0.90595 |  | 1091.221 | 0.7083 |
| 1092.6 | 0.90605 |  | 1092.649 | 0.71051 |
| 1094.028 | 0.90623 |  | 1094.078 | 0.71283 |
| 1095.457 | 0.90649 |  | 1095.506 | 0.71523 |
| 1096.885 | 0.90684 |  | 1096.934 | 0.71771 |
| 1098.313 | 0.90726 |  | 1098.363 | 0.72029 |
| 1099.741 | 0.90774 |  | 1099.791 | 0.72299 |
| 1101.17 | 0.90828 |  | 1101.219 | 0.72585 |
| 1102.598 | 0.90882 |  | 1102.648 | 0.72886 |
| 1104.026 | 0.90934 |  | 1104.076 | 0.73201 |
| 1105.454 | 0.90983 |  | 1105.504 | 0.73527 |
| 1106.882 | 0.9103 |  | 1106.932 | 0.7386 |
| 1108.311 | 0.91077 |  | 1108.361 | 0.74196 |
| 1109.739 | 0.91125 |  | 1109.789 | 0.74536 |
| 1111.167 | 0.91173 |  | 1111.217 | 0.7488 |
| 1112.595 | 0.91221 |  | 1112.646 | 0.75223 |
| 1114.024 | 0.91268 |  | 1114.074 | 0.75562 |
| 1115.452 | 0.91315 |  | 1115.502 | 0.759 |
| 1116.88 | 0.9136 |  | 1116.931 | 0.7624 |
| 1118.308 | 0.91401 |  | 1118.359 | 0.76582 |
| 1119.737 | 0.91441 |  | 1119.787 | 0.76922 |
| 1121.165 | 0.91481 |  | 1121.215 | 0.7726 |
| 1122.593 | 0.91522 |  | 1122.644 | 0.77597 |
| 1124.021 | 0.91565 |  | 1124.072 | 0.77937 |
| 1125.45 | 0.91609 |  | 1125.5 | 0.78278 |
| 1126.878 | 0.91654 |  | 1126.929 | 0.7862 |
| 1128.306 | 0.91697 |  | 1128.357 | 0.78964 |
| 1129.734 | 0.91738 |  | 1129.785 | 0.7931 |
| 1131.162 | 0.91775 |  | 1131.214 | 0.79658 |
| 1132.591 | 0.9181 |  | 1132.642 | 0.80003 |
| 1134.019 | 0.91844 |  | 1134.07 | 0.80339 |
| 1135.447 | 0.91877 |  | 1135.498 | 0.80667 |
| 1136.875 | 0.91907 |  | 1136.927 | 0.80988 |
| 1138.304 | 0.91933 |  | 1138.355 | 0.813 |
| 1139.732 | 0.91958 |  | 1139.783 | 0.81605 |
| 1141.16 | 0.91981 |  | 1141.212 | 0.81904 |
| 1142.588 | 0.92003 |  | 1142.64 | 0.82201 |
| 1144.017 | 0.92021 |  | 1144.068 | 0.825 |
| 1145.445 | 0.92034 |  | 1145.497 | 0.82799 |
| 1146.873 | 0.92044 |  | 1146.925 | 0.83097 |
| 1148.301 | 0.92053 |  | 1148.353 | 0.8339 |
| 1149.73 | 0.92062 |  | 1149.781 | 0.83679 |
| 1151.158 | 0.92073 |  | 1151.21 | 0.83965 |
| 1152.586 | 0.92086 |  | 1152.638 | 0.84251 |
| 1154.014 | 0.92102 |  | 1154.066 | 0.84534 |
| 1155.442 | 0.92119 |  | 1155.495 | 0.84807 |
| 1156.871 | 0.92134 |  | 1156.923 | 0.8506 |
| 1158.299 | 0.92149 |  | 1158.351 | 0.85291 |
| 1159.727 | 0.92165 |  | 1159.78 | 0.855 |
| 1161.155 | 0.92184 |  | 1161.208 | 0.8569 |
| 1162.584 | 0.92204 |  | 1162.636 | 0.85862 |
| 1164.012 | 0.92225 |  | 1164.064 | 0.86016 |
| 1165.44 | 0.92242 |  | 1165.493 | 0.86149 |
| 1166.868 | 0.92256 |  | 1166.921 | 0.86262 |
| 1168.297 | 0.92268 |  | 1168.349 | 0.86357 |
| 1169.725 | 0.92281 |  | 1169.778 | 0.86439 |
| 1171.153 | 0.92296 |  | 1171.206 | 0.86509 |
| 1172.581 | 0.92312 |  | 1172.634 | 0.86573 |
| 1174.01 | 0.92328 |  | 1174.063 | 0.86634 |
| 1175.438 | 0.92342 |  | 1175.491 | 0.86693 |
| 1176.866 | 0.92355 |  | 1176.919 | 0.86752 |
| 1178.294 | 0.92365 |  | 1178.347 | 0.8681 |
| 1179.722 | 0.92371 |  | 1179.776 | 0.86868 |
| 1181.151 | 0.92372 |  | 1181.204 | 0.86924 |
| 1182.579 | 0.92372 |  | 1182.632 | 0.86979 |
| 1184.007 | 0.92373 |  | 1184.061 | 0.87032 |
| 1185.435 | 0.92376 |  | 1185.489 | 0.87086 |
| 1186.864 | 0.92381 |  | 1186.917 | 0.87136 |
| 1188.292 | 0.92385 |  | 1188.346 | 0.87179 |
| 1189.72 | 0.92388 |  | 1189.774 | 0.87212 |
| 1191.148 | 0.92392 |  | 1191.202 | 0.87236 |
| 1192.577 | 0.92396 |  | 1192.63 | 0.87253 |
| 1194.005 | 0.924 |  | 1194.059 | 0.87264 |
| 1195.433 | 0.92403 |  | 1195.487 | 0.87271 |
| 1196.861 | 0.92404 |  | 1196.915 | 0.87275 |
| 1198.29 | 0.92403 |  | 1198.344 | 0.87273 |
| 1199.718 | 0.92402 |  | 1199.772 | 0.87267 |
| 1201.146 | 0.92402 |  | 1201.2 | 0.87254 |
| 1202.574 | 0.92403 |  | 1202.629 | 0.87234 |
| 1204.002 | 0.92405 |  | 1204.057 | 0.87208 |
| 1205.431 | 0.92407 |  | 1205.485 | 0.87179 |
| 1206.859 | 0.92409 |  | 1206.913 | 0.87146 |
| 1208.287 | 0.92412 |  | 1208.342 | 0.87113 |
| 1209.715 | 0.92418 |  | 1209.77 | 0.87077 |
| 1211.144 | 0.92424 |  | 1211.198 | 0.87038 |
| 1212.572 | 0.9243 |  | 1212.627 | 0.86993 |
| 1214 | 0.92435 |  | 1214.055 | 0.86943 |
| 1215.428 | 0.92438 |  | 1215.483 | 0.86889 |
| 1216.857 | 0.92439 |  | 1216.912 | 0.86832 |
| 1218.285 | 0.9244 |  | 1218.34 | 0.86772 |
| 1219.713 | 0.92443 |  | 1219.768 | 0.8671 |
| 1221.141 | 0.92446 |  | 1221.196 | 0.86649 |
| 1222.57 | 0.92451 |  | 1222.625 | 0.86588 |
| 1223.998 | 0.92456 |  | 1224.053 | 0.86527 |
| 1225.426 | 0.9246 |  | 1225.481 | 0.86465 |
| 1226.854 | 0.92464 |  | 1226.91 | 0.86404 |
| 1228.282 | 0.92467 |  | 1228.338 | 0.86345 |
| 1229.711 | 0.92471 |  | 1229.766 | 0.86289 |
| 1231.139 | 0.92476 |  | 1231.195 | 0.86238 |
| 1232.567 | 0.92482 |  | 1232.623 | 0.86193 |
| 1233.995 | 0.92489 |  | 1234.051 | 0.86153 |
| 1235.424 | 0.92496 |  | 1235.479 | 0.86117 |
| 1236.852 | 0.92505 |  | 1236.908 | 0.86079 |
| 1238.28 | 0.92513 |  | 1238.336 | 0.86029 |
| 1239.708 | 0.9252 |  | 1239.764 | 0.85966 |
| 1241.137 | 0.92525 |  | 1241.193 | 0.85905 |
| 1242.565 | 0.92528 |  | 1242.621 | 0.85861 |
| 1243.993 | 0.9253 |  | 1244.049 | 0.85827 |
| 1245.421 | 0.92533 |  | 1245.478 | 0.85784 |
| 1246.85 | 0.92536 |  | 1246.906 | 0.85722 |
| 1248.278 | 0.92541 |  | 1248.334 | 0.85651 |
| 1249.706 | 0.92546 |  | 1249.762 | 0.85583 |
| 1251.134 | 0.9255 |  | 1251.191 | 0.85524 |
| 1252.562 | 0.92553 |  | 1252.619 | 0.85472 |
| 1253.991 | 0.92555 |  | 1254.047 | 0.85419 |
| 1255.419 | 0.92558 |  | 1255.476 | 0.85363 |
| 1256.847 | 0.92561 |  | 1256.904 | 0.85305 |
| 1258.275 | 0.92564 |  | 1258.332 | 0.85246 |
| 1259.704 | 0.92568 |  | 1259.761 | 0.85191 |
| 1261.132 | 0.9257 |  | 1261.189 | 0.8514 |
| 1262.56 | 0.92569 |  | 1262.617 | 0.85095 |
| 1263.988 | 0.92565 |  | 1264.045 | 0.85053 |
| 1265.417 | 0.9256 |  | 1265.474 | 0.8501 |
| 1266.845 | 0.92556 |  | 1266.902 | 0.84964 |
| 1268.273 | 0.92551 |  | 1268.33 | 0.84913 |
| 1269.701 | 0.92544 |  | 1269.759 | 0.84857 |
| 1271.13 | 0.92535 |  | 1271.187 | 0.84798 |
| 1272.558 | 0.92522 |  | 1272.615 | 0.84737 |
| 1273.986 | 0.92507 |  | 1274.044 | 0.84675 |
| 1275.414 | 0.92494 |  | 1275.472 | 0.8461 |
| 1276.842 | 0.92484 |  | 1276.9 | 0.84543 |
| 1278.271 | 0.92475 |  | 1278.328 | 0.84473 |
| 1279.699 | 0.92466 |  | 1279.757 | 0.84404 |
| 1281.127 | 0.92453 |  | 1281.185 | 0.84337 |
| 1282.555 | 0.92439 |  | 1282.613 | 0.84272 |
| 1283.984 | 0.92425 |  | 1284.042 | 0.84206 |
| 1285.412 | 0.92412 |  | 1285.47 | 0.84139 |
| 1286.84 | 0.92399 |  | 1286.898 | 0.84068 |
| 1288.268 | 0.92385 |  | 1288.327 | 0.83995 |
| 1289.697 | 0.92367 |  | 1289.755 | 0.83919 |
| 1291.125 | 0.92347 |  | 1291.183 | 0.83839 |
| 1292.553 | 0.92327 |  | 1292.611 | 0.83754 |
| 1293.981 | 0.92306 |  | 1294.04 | 0.83663 |
| 1295.41 | 0.92285 |  | 1295.468 | 0.83567 |
| 1296.838 | 0.92262 |  | 1296.896 | 0.83468 |
| 1298.266 | 0.92239 |  | 1298.325 | 0.83368 |
| 1299.694 | 0.92215 |  | 1299.753 | 0.83267 |
| 1301.122 | 0.92192 |  | 1301.181 | 0.83166 |
| 1302.551 | 0.9217 |  | 1302.61 | 0.83066 |
| 1303.979 | 0.92149 |  | 1304.038 | 0.8297 |
| 1305.407 | 0.92129 |  | 1305.466 | 0.82875 |
| 1306.835 | 0.92107 |  | 1306.894 | 0.82778 |
| 1308.264 | 0.92083 |  | 1308.323 | 0.82678 |
| 1309.692 | 0.9206 |  | 1309.751 | 0.82578 |
| 1311.12 | 0.92038 |  | 1311.179 | 0.8248 |
| 1312.548 | 0.92018 |  | 1312.608 | 0.82381 |
| 1313.977 | 0.92 |  | 1314.036 | 0.82282 |
| 1315.405 | 0.91983 |  | 1315.464 | 0.82185 |
| 1316.833 | 0.91968 |  | 1316.893 | 0.8209 |
| 1318.261 | 0.91951 |  | 1318.321 | 0.81989 |
| 1319.69 | 0.91932 |  | 1319.749 | 0.81882 |
| 1321.118 | 0.91911 |  | 1321.177 | 0.81774 |
| 1322.546 | 0.9189 |  | 1322.606 | 0.81667 |
| 1323.974 | 0.91871 |  | 1324.034 | 0.81559 |
| 1325.402 | 0.91853 |  | 1325.462 | 0.81446 |
| 1326.831 | 0.91837 |  | 1326.891 | 0.81326 |
| 1328.259 | 0.9182 |  | 1328.319 | 0.81199 |
| 1329.687 | 0.91801 |  | 1329.747 | 0.81066 |
| 1331.115 | 0.91778 |  | 1331.176 | 0.80928 |
| 1332.544 | 0.91752 |  | 1332.604 | 0.80783 |
| 1333.972 | 0.91728 |  | 1334.032 | 0.80635 |
| 1335.4 | 0.91713 |  | 1335.46 | 0.80488 |
| 1336.828 | 0.91712 |  | 1336.889 | 0.80337 |
| 1338.257 | 0.91722 |  | 1338.317 | 0.80172 |
| 1339.685 | 0.91732 |  | 1339.745 | 0.79988 |
| 1341.113 | 0.91727 |  | 1341.174 | 0.79804 |
| 1342.541 | 0.91706 |  | 1342.602 | 0.79634 |
| 1343.97 | 0.91677 |  | 1344.03 | 0.79467 |
| 1345.398 | 0.9165 |  | 1345.459 | 0.79287 |
| 1346.826 | 0.91628 |  | 1346.887 | 0.79092 |
| 1348.254 | 0.91611 |  | 1348.315 | 0.78885 |
| 1349.682 | 0.91595 |  | 1349.743 | 0.78668 |
| 1351.111 | 0.9158 |  | 1351.172 | 0.78443 |
| 1352.539 | 0.91565 |  | 1352.6 | 0.78212 |
| 1353.967 | 0.91551 |  | 1354.028 | 0.77969 |
| 1355.395 | 0.91539 |  | 1355.457 | 0.7771 |
| 1356.824 | 0.9153 |  | 1356.885 | 0.77445 |
| 1358.252 | 0.91528 |  | 1358.313 | 0.77182 |
| 1359.68 | 0.91537 |  | 1359.742 | 0.76908 |
| 1361.108 | 0.91554 |  | 1361.17 | 0.76591 |
| 1362.537 | 0.9157 |  | 1362.598 | 0.76212 |
| 1363.965 | 0.91575 |  | 1364.026 | 0.75824 |
| 1365.393 | 0.91567 |  | 1365.455 | 0.75477 |
| 1366.821 | 0.91553 |  | 1366.883 | 0.75141 |
| 1368.25 | 0.9154 |  | 1368.311 | 0.74775 |
| 1369.678 | 0.91528 |  | 1369.74 | 0.74393 |
| 1371.106 | 0.9152 |  | 1371.168 | 0.74021 |
| 1372.534 | 0.91511 |  | 1372.596 | 0.73625 |
| 1373.962 | 0.91499 |  | 1374.025 | 0.73174 |
| 1375.391 | 0.91478 |  | 1375.453 | 0.7271 |
| 1376.819 | 0.91449 |  | 1376.881 | 0.72289 |
| 1378.247 | 0.91419 |  | 1378.309 | 0.71895 |
| 1379.675 | 0.91393 |  | 1379.738 | 0.71496 |
| 1381.104 | 0.91375 |  | 1381.166 | 0.71091 |
| 1382.532 | 0.91367 |  | 1382.594 | 0.70706 |
| 1383.96 | 0.91369 |  | 1384.023 | 0.70338 |
| 1385.388 | 0.91379 |  | 1385.451 | 0.69942 |
| 1386.817 | 0.91392 |  | 1386.879 | 0.69487 |
| 1388.245 | 0.91397 |  | 1388.308 | 0.69015 |
| 1389.673 | 0.91392 |  | 1389.736 | 0.68601 |
| 1391.101 | 0.9138 |  | 1391.164 | 0.68245 |
| 1392.53 | 0.91373 |  | 1392.592 | 0.67902 |
| 1393.958 | 0.91373 |  | 1394.021 | 0.67546 |
| 1395.386 | 0.91377 |  | 1395.449 | 0.67225 |
| 1396.814 | 0.91372 |  | 1396.877 | 0.67007 |
| 1398.242 | 0.91351 |  | 1398.306 | 0.66901 |
| 1399.671 | 0.91317 |  | 1399.734 | 0.66902 |
| 1401.099 | 0.91279 |  | 1401.162 | 0.67019 |
| 1402.527 | 0.91243 |  | 1402.591 | 0.67258 |
| 1403.955 | 0.91212 |  | 1404.019 | 0.67648 |
| 1405.384 | 0.91182 |  | 1405.447 | 0.682 |
| 1406.812 | 0.91151 |  | 1406.875 | 0.68841 |
| 1408.24 | 0.9112 |  | 1408.304 | 0.69518 |
| 1409.668 | 0.91089 |  | 1409.732 | 0.70268 |
| 1411.097 | 0.91061 |  | 1411.16 | 0.711 |
| 1412.525 | 0.91036 |  | 1412.589 | 0.71957 |
| 1413.953 | 0.91016 |  | 1414.017 | 0.72777 |
| 1415.381 | 0.91006 |  | 1415.445 | 0.73558 |
| 1416.81 | 0.91007 |  | 1416.874 | 0.74393 |
| 1418.238 | 0.91012 |  | 1418.302 | 0.754 |
| 1419.666 | 0.91008 |  | 1419.73 | 0.76428 |
| 1421.094 | 0.90989 |  | 1421.158 | 0.77199 |
| 1422.522 | 0.9096 |  | 1422.587 | 0.77762 |
| 1423.951 | 0.90931 |  | 1424.015 | 0.78258 |
| 1425.379 | 0.90906 |  | 1425.443 | 0.78694 |
| 1426.807 | 0.90888 |  | 1426.872 | 0.79041 |
| 1428.235 | 0.90878 |  | 1428.3 | 0.79327 |
| 1429.664 | 0.90872 |  | 1429.728 | 0.79591 |
| 1431.092 | 0.90865 |  | 1431.157 | 0.79823 |
| 1432.52 | 0.90858 |  | 1432.585 | 0.79998 |
| 1433.948 | 0.90856 |  | 1434.013 | 0.80126 |
| 1435.377 | 0.90863 |  | 1435.441 | 0.80234 |
| 1436.805 | 0.90871 |  | 1436.87 | 0.80332 |
| 1438.233 | 0.90866 |  | 1438.298 | 0.80419 |
| 1439.661 | 0.90846 |  | 1439.726 | 0.80509 |
| 1441.09 | 0.90821 |  | 1441.155 | 0.80631 |
| 1442.518 | 0.90803 |  | 1442.583 | 0.80797 |
| 1443.946 | 0.90795 |  | 1444.011 | 0.8101 |
| 1445.374 | 0.90798 |  | 1445.44 | 0.81282 |
| 1446.802 | 0.90807 |  | 1446.868 | 0.81638 |
| 1448.231 | 0.90812 |  | 1448.296 | 0.82078 |
| 1449.659 | 0.90809 |  | 1449.724 | 0.82549 |
| 1451.087 | 0.908 |  | 1451.153 | 0.83008 |
| 1452.515 | 0.90794 |  | 1452.581 | 0.83442 |
| 1453.944 | 0.90804 |  | 1454.009 | 0.83854 |
| 1455.372 | 0.90838 |  | 1455.438 | 0.84289 |
| 1456.8 | 0.90885 |  | 1456.866 | 0.84833 |
| 1458.228 | 0.90903 |  | 1458.294 | 0.85361 |
| 1459.657 | 0.90875 |  | 1459.723 | 0.85669 |
| 1461.085 | 0.90833 |  | 1461.151 | 0.85843 |
| 1462.513 | 0.90803 |  | 1462.579 | 0.85998 |
| 1463.941 | 0.90793 |  | 1464.007 | 0.86182 |
| 1465.37 | 0.90794 |  | 1465.436 | 0.86393 |
| 1466.798 | 0.90792 |  | 1466.864 | 0.86586 |
| 1468.226 | 0.90786 |  | 1468.292 | 0.86737 |
| 1469.654 | 0.90788 |  | 1469.721 | 0.86867 |
| 1471.082 | 0.90812 |  | 1471.149 | 0.87011 |
| 1472.511 | 0.90854 |  | 1472.577 | 0.87199 |
| 1473.939 | 0.90887 |  | 1474.006 | 0.87399 |
| 1475.367 | 0.90885 |  | 1475.434 | 0.87568 |
| 1476.795 | 0.90858 |  | 1476.862 | 0.87707 |
| 1478.224 | 0.90826 |  | 1478.29 | 0.87828 |
| 1479.652 | 0.908 |  | 1479.719 | 0.87934 |
| 1481.08 | 0.90781 |  | 1481.147 | 0.88033 |
| 1482.508 | 0.90768 |  | 1482.575 | 0.88121 |
| 1483.937 | 0.9076 |  | 1484.004 | 0.88191 |
| 1485.365 | 0.90763 |  | 1485.432 | 0.88245 |
| 1486.793 | 0.90781 |  | 1486.86 | 0.88287 |
| 1488.221 | 0.9081 |  | 1488.289 | 0.8832 |
| 1489.65 | 0.90829 |  | 1489.717 | 0.88355 |
| 1491.078 | 0.90821 |  | 1491.145 | 0.88394 |
| 1492.506 | 0.90796 |  | 1492.573 | 0.88426 |
| 1493.934 | 0.90774 |  | 1494.002 | 0.88444 |
| 1495.362 | 0.90767 |  | 1495.43 | 0.88445 |
| 1496.791 | 0.90776 |  | 1496.858 | 0.88428 |
| 1498.219 | 0.90783 |  | 1498.287 | 0.88409 |
| 1499.647 | 0.90773 |  | 1499.715 | 0.88399 |
| 1501.075 | 0.90747 |  | 1501.143 | 0.88392 |
| 1502.504 | 0.90722 |  | 1502.572 | 0.88382 |
| 1503.932 | 0.90716 |  | 1504 | 0.88366 |
| 1505.36 | 0.90749 |  | 1505.428 | 0.88322 |
| 1506.788 | 0.90817 |  | 1506.856 | 0.88216 |
| 1508.217 | 0.9086 |  | 1508.285 | 0.881 |
| 1509.645 | 0.90836 |  | 1509.713 | 0.88038 |
| 1511.073 | 0.90779 |  | 1511.141 | 0.87992 |
| 1512.501 | 0.90728 |  | 1512.57 | 0.87943 |
| 1513.93 | 0.90697 |  | 1513.998 | 0.87886 |
| 1515.358 | 0.90686 |  | 1515.426 | 0.87816 |
| 1516.786 | 0.90689 |  | 1516.855 | 0.87736 |
| 1518.214 | 0.90701 |  | 1518.283 | 0.87664 |
| 1519.642 | 0.90719 |  | 1519.711 | 0.87599 |
| 1521.071 | 0.90738 |  | 1521.139 | 0.87526 |
| 1522.499 | 0.90749 |  | 1522.568 | 0.87453 |
| 1523.927 | 0.90743 |  | 1523.996 | 0.87402 |
| 1525.355 | 0.90721 |  | 1525.424 | 0.8736 |
| 1526.784 | 0.90689 |  | 1526.853 | 0.87311 |
| 1528.212 | 0.90657 |  | 1528.281 | 0.87264 |
| 1529.64 | 0.90633 |  | 1529.709 | 0.87229 |
| 1531.068 | 0.90623 |  | 1531.138 | 0.87193 |
| 1532.497 | 0.90628 |  | 1532.566 | 0.87138 |
| 1533.925 | 0.90635 |  | 1533.994 | 0.87074 |
| 1535.353 | 0.90636 |  | 1535.422 | 0.87028 |
| 1536.781 | 0.90637 |  | 1536.851 | 0.86997 |
| 1538.21 | 0.90659 |  | 1538.279 | 0.86951 |
| 1539.638 | 0.90723 |  | 1539.707 | 0.86863 |
| 1541.066 | 0.908 |  | 1541.136 | 0.86767 |
| 1542.494 | 0.9082 |  | 1542.564 | 0.86727 |
| 1543.922 | 0.90774 |  | 1543.992 | 0.86709 |
| 1545.351 | 0.9071 |  | 1545.421 | 0.8669 |
| 1546.779 | 0.90655 |  | 1546.849 | 0.86668 |
| 1548.207 | 0.90619 |  | 1548.277 | 0.86638 |
| 1549.635 | 0.90599 |  | 1549.705 | 0.86594 |
| 1551.064 | 0.90591 |  | 1551.134 | 0.86544 |
| 1552.492 | 0.90586 |  | 1552.562 | 0.86495 |
| 1553.92 | 0.90582 |  | 1553.99 | 0.86445 |
| 1555.348 | 0.90587 |  | 1555.419 | 0.86397 |
| 1556.777 | 0.90615 |  | 1556.847 | 0.86346 |
| 1558.205 | 0.90665 |  | 1558.275 | 0.86259 |
| 1559.633 | 0.90688 |  | 1559.704 | 0.86151 |
| 1561.061 | 0.90657 |  | 1561.132 | 0.86101 |
| 1562.49 | 0.90603 |  | 1562.56 | 0.86074 |
| 1563.918 | 0.90555 |  | 1563.988 | 0.86037 |
| 1565.346 | 0.90523 |  | 1565.417 | 0.85987 |
| 1566.774 | 0.90513 |  | 1566.845 | 0.8593 |
| 1568.202 | 0.90517 |  | 1568.273 | 0.85859 |
| 1569.631 | 0.90521 |  | 1569.702 | 0.85772 |
| 1571.059 | 0.90513 |  | 1571.13 | 0.85696 |
| 1572.487 | 0.90495 |  | 1572.558 | 0.85643 |
| 1573.915 | 0.90481 |  | 1573.986 | 0.85587 |
| 1575.344 | 0.9048 |  | 1575.415 | 0.85495 |
| 1576.772 | 0.90481 |  | 1576.843 | 0.85387 |
| 1578.2 | 0.90465 |  | 1578.271 | 0.85316 |
| 1579.628 | 0.90431 |  | 1579.7 | 0.85275 |
| 1581.057 | 0.90394 |  | 1581.128 | 0.85233 |
| 1582.485 | 0.90364 |  | 1582.556 | 0.8518 |
| 1583.913 | 0.90342 |  | 1583.985 | 0.85123 |
| 1585.341 | 0.90326 |  | 1585.413 | 0.85067 |
| 1586.77 | 0.90309 |  | 1586.841 | 0.85012 |
| 1588.198 | 0.90292 |  | 1588.269 | 0.84958 |
| 1589.626 | 0.90274 |  | 1589.698 | 0.84906 |
| 1591.054 | 0.90255 |  | 1591.126 | 0.84858 |
| 1592.482 | 0.90237 |  | 1592.554 | 0.84817 |
| 1593.911 | 0.90217 |  | 1593.983 | 0.8478 |
| 1595.339 | 0.90193 |  | 1595.411 | 0.84745 |
| 1596.767 | 0.90167 |  | 1596.839 | 0.84715 |
| 1598.195 | 0.90141 |  | 1598.268 | 0.84692 |
| 1599.624 | 0.90118 |  | 1599.696 | 0.84672 |
| 1601.052 | 0.90097 |  | 1601.124 | 0.84652 |
| 1602.48 | 0.90074 |  | 1602.552 | 0.84634 |
| 1603.908 | 0.90048 |  | 1603.981 | 0.84621 |
| 1605.337 | 0.90018 |  | 1605.409 | 0.84611 |
| 1606.765 | 0.89986 |  | 1606.837 | 0.84602 |
| 1608.193 | 0.8995 |  | 1608.266 | 0.84595 |
| 1609.621 | 0.89909 |  | 1609.694 | 0.84595 |
| 1611.05 | 0.89863 |  | 1611.122 | 0.84602 |
| 1612.478 | 0.89818 |  | 1612.551 | 0.84609 |
| 1613.906 | 0.89784 |  | 1613.979 | 0.84611 |
| 1615.334 | 0.89762 |  | 1615.407 | 0.84604 |
| 1616.762 | 0.89742 |  | 1616.835 | 0.84598 |
| 1618.191 | 0.89712 |  | 1618.264 | 0.84608 |
| 1619.619 | 0.89671 |  | 1619.692 | 0.84627 |
| 1621.047 | 0.8963 |  | 1621.12 | 0.84642 |
| 1622.475 | 0.89594 |  | 1622.549 | 0.84652 |
| 1623.904 | 0.89565 |  | 1623.977 | 0.84666 |
| 1625.332 | 0.89535 |  | 1625.405 | 0.84688 |
| 1626.76 | 0.89502 |  | 1626.834 | 0.84717 |
| 1628.188 | 0.89467 |  | 1628.262 | 0.84753 |
| 1629.617 | 0.8943 |  | 1629.69 | 0.84793 |
| 1631.045 | 0.89396 |  | 1631.118 | 0.84829 |
| 1632.473 | 0.89377 |  | 1632.547 | 0.84856 |
| 1633.901 | 0.8939 |  | 1633.975 | 0.84871 |
| 1635.33 | 0.89439 |  | 1635.403 | 0.84883 |
| 1636.758 | 0.89493 |  | 1636.832 | 0.84914 |
| 1638.186 | 0.89509 |  | 1638.26 | 0.84962 |
| 1639.614 | 0.89493 |  | 1639.688 | 0.8501 |
| 1641.042 | 0.89468 |  | 1641.117 | 0.85053 |
| 1642.471 | 0.89456 |  | 1642.545 | 0.85089 |
| 1643.899 | 0.89475 |  | 1643.973 | 0.85117 |
| 1645.327 | 0.89536 |  | 1645.401 | 0.85135 |
| 1646.755 | 0.89622 |  | 1646.83 | 0.85153 |
| 1648.184 | 0.89691 |  | 1648.258 | 0.85187 |
| 1649.612 | 0.89725 |  | 1649.686 | 0.85227 |
| 1651.04 | 0.89759 |  | 1651.115 | 0.85263 |
| 1652.468 | 0.89831 |  | 1652.543 | 0.85308 |
| 1653.897 | 0.89919 |  | 1653.971 | 0.85373 |
| 1655.325 | 0.89956 |  | 1655.4 | 0.8544 |
| 1656.753 | 0.89942 |  | 1656.828 | 0.85497 |
| 1658.181 | 0.89919 |  | 1658.256 | 0.85548 |
| 1659.61 | 0.89919 |  | 1659.684 | 0.85595 |
| 1661.038 | 0.89952 |  | 1661.113 | 0.85645 |
| 1662.466 | 0.90008 |  | 1662.541 | 0.85707 |
| 1663.894 | 0.90058 |  | 1663.969 | 0.85779 |
| 1665.322 | 0.90091 |  | 1665.398 | 0.85842 |
| 1666.751 | 0.90125 |  | 1666.826 | 0.85893 |
| 1668.179 | 0.90179 |  | 1668.254 | 0.85943 |
| 1669.607 | 0.90257 |  | 1669.683 | 0.86011 |
| 1671.035 | 0.90332 |  | 1671.111 | 0.86092 |
| 1672.464 | 0.90383 |  | 1672.539 | 0.86169 |
| 1673.892 | 0.90416 |  | 1673.967 | 0.86248 |
| 1675.32 | 0.90446 |  | 1675.396 | 0.86338 |
| 1676.748 | 0.90475 |  | 1676.824 | 0.86426 |
| 1678.177 | 0.90497 |  | 1678.252 | 0.86499 |
| 1679.605 | 0.90515 |  | 1679.681 | 0.86561 |
| 1681.033 | 0.90546 |  | 1681.109 | 0.86618 |
| 1682.461 | 0.90609 |  | 1682.537 | 0.86681 |
| 1683.89 | 0.90701 |  | 1683.966 | 0.86774 |
| 1685.318 | 0.90778 |  | 1685.394 | 0.86887 |
| 1686.746 | 0.90808 |  | 1686.822 | 0.86976 |
| 1688.174 | 0.90807 |  | 1688.25 | 0.87049 |
| 1689.602 | 0.90798 |  | 1689.679 | 0.87123 |
| 1691.031 | 0.90793 |  | 1691.107 | 0.87191 |
| 1692.459 | 0.90799 |  | 1692.535 | 0.87243 |
| 1693.887 | 0.90822 |  | 1693.964 | 0.87284 |
| 1695.315 | 0.90869 |  | 1695.392 | 0.87324 |
| 1696.744 | 0.90939 |  | 1696.82 | 0.87362 |
| 1698.172 | 0.91017 |  | 1698.249 | 0.8739 |
| 1699.6 | 0.9108 |  | 1699.677 | 0.87411 |
| 1701.028 | 0.91114 |  | 1701.105 | 0.8743 |
| 1702.457 | 0.91126 |  | 1702.533 | 0.87445 |
| 1703.885 | 0.91127 |  | 1703.962 | 0.87451 |
| 1705.313 | 0.91127 |  | 1705.39 | 0.87452 |
| 1706.741 | 0.91128 |  | 1706.818 | 0.87457 |
| 1708.17 | 0.91127 |  | 1708.247 | 0.87466 |
| 1709.598 | 0.91125 |  | 1709.675 | 0.87473 |
| 1711.026 | 0.91126 |  | 1711.103 | 0.87477 |
| 1712.454 | 0.91138 |  | 1712.532 | 0.87476 |
| 1713.882 | 0.9117 |  | 1713.96 | 0.8747 |
| 1715.311 | 0.91229 |  | 1715.388 | 0.87459 |
| 1716.739 | 0.91297 |  | 1716.816 | 0.87448 |
| 1718.167 | 0.91336 |  | 1718.245 | 0.87446 |
| 1719.595 | 0.91331 |  | 1719.673 | 0.87455 |
| 1721.024 | 0.91305 |  | 1721.101 | 0.87462 |
| 1722.452 | 0.91279 |  | 1722.53 | 0.87465 |
| 1723.88 | 0.91264 |  | 1723.958 | 0.87462 |
| 1725.308 | 0.91259 |  | 1725.386 | 0.87454 |
| 1726.737 | 0.9126 |  | 1726.815 | 0.87439 |
| 1728.165 | 0.91263 |  | 1728.243 | 0.87416 |
| 1729.593 | 0.91272 |  | 1729.671 | 0.87389 |
| 1731.021 | 0.91293 |  | 1731.099 | 0.87361 |
| 1732.45 | 0.91327 |  | 1732.528 | 0.87334 |
| 1733.878 | 0.91362 |  | 1733.956 | 0.87307 |
| 1735.306 | 0.91374 |  | 1735.384 | 0.87292 |
| 1736.734 | 0.91363 |  | 1736.813 | 0.87283 |
| 1738.163 | 0.91343 |  | 1738.241 | 0.87273 |
| 1739.591 | 0.91328 |  | 1739.669 | 0.87259 |
| 1741.019 | 0.91323 |  | 1741.098 | 0.8725 |
| 1742.447 | 0.91322 |  | 1742.526 | 0.8725 |
| 1743.875 | 0.91319 |  | 1743.954 | 0.87254 |
| 1745.304 | 0.91318 |  | 1745.382 | 0.87257 |
| 1746.732 | 0.91325 |  | 1746.811 | 0.87263 |
| 1748.16 | 0.91341 |  | 1748.239 | 0.87277 |
| 1749.588 | 0.91356 |  | 1749.667 | 0.87301 |
| 1751.017 | 0.91355 |  | 1751.096 | 0.8733 |
| 1752.445 | 0.91339 |  | 1752.524 | 0.87356 |
| 1753.873 | 0.91317 |  | 1753.952 | 0.87379 |
| 1755.301 | 0.91299 |  | 1755.381 | 0.874 |
| 1756.73 | 0.91291 |  | 1756.809 | 0.87421 |
| 1758.158 | 0.91292 |  | 1758.237 | 0.8744 |
| 1759.586 | 0.91297 |  | 1759.665 | 0.87456 |
| 1761.014 | 0.91303 |  | 1761.094 | 0.87473 |
| 1762.443 | 0.91306 |  | 1762.522 | 0.87499 |
| 1763.871 | 0.91303 |  | 1763.95 | 0.87529 |
| 1765.299 | 0.91295 |  | 1765.379 | 0.87559 |
| 1766.727 | 0.91289 |  | 1766.807 | 0.87587 |
| 1768.155 | 0.91293 |  | 1768.235 | 0.87612 |
| 1769.584 | 0.91315 |  | 1769.664 | 0.87632 |
| 1771.012 | 0.91348 |  | 1771.092 | 0.87652 |
| 1772.44 | 0.91378 |  | 1772.52 | 0.87685 |
| 1773.868 | 0.91384 |  | 1773.948 | 0.87729 |
| 1775.297 | 0.91367 |  | 1775.377 | 0.87772 |
| 1776.725 | 0.91338 |  | 1776.805 | 0.87808 |
| 1778.153 | 0.91314 |  | 1778.233 | 0.87837 |
| 1779.581 | 0.913 |  | 1779.662 | 0.87866 |
| 1781.01 | 0.91295 |  | 1781.09 | 0.879 |
| 1782.438 | 0.91294 |  | 1782.518 | 0.8794 |
| 1783.866 | 0.91291 |  | 1783.947 | 0.87982 |
| 1785.294 | 0.91284 |  | 1785.375 | 0.88023 |
| 1786.723 | 0.91274 |  | 1786.803 | 0.88059 |
| 1788.151 | 0.91266 |  | 1788.231 | 0.88089 |
| 1789.579 | 0.91268 |  | 1789.66 | 0.88117 |
| 1791.007 | 0.91286 |  | 1791.088 | 0.88149 |
| 1792.435 | 0.91311 |  | 1792.516 | 0.88192 |
| 1793.864 | 0.91326 |  | 1793.945 | 0.88239 |
| 1795.292 | 0.91321 |  | 1795.373 | 0.8828 |
| 1796.72 | 0.91306 |  | 1796.801 | 0.88316 |
| 1798.148 | 0.91294 |  | 1798.23 | 0.88351 |
| 1799.577 | 0.9129 |  | 1799.658 | 0.88389 |
| 1801.005 | 0.9129 |  | 1801.086 | 0.88432 |
| 1802.433 | 0.91286 |  | 1802.514 | 0.88475 |
| 1803.861 | 0.91275 |  | 1803.943 | 0.88511 |
| 1805.29 | 0.91262 |  | 1805.371 | 0.88541 |
| 1806.718 | 0.91256 |  | 1806.799 | 0.8857 |
| 1808.146 | 0.91262 |  | 1808.228 | 0.88597 |
| 1809.574 | 0.9128 |  | 1809.656 | 0.88628 |
| 1811.003 | 0.91301 |  | 1811.084 | 0.88664 |
| 1812.431 | 0.91315 |  | 1812.513 | 0.88705 |
| 1813.859 | 0.9132 |  | 1813.941 | 0.88746 |
| 1815.287 | 0.91318 |  | 1815.369 | 0.88782 |
| 1816.715 | 0.91313 |  | 1816.797 | 0.88807 |
| 1818.144 | 0.91305 |  | 1818.226 | 0.8882 |
| 1819.572 | 0.91296 |  | 1819.654 | 0.88825 |
| 1821 | 0.91289 |  | 1821.082 | 0.8883 |
| 1822.428 | 0.9129 |  | 1822.511 | 0.88839 |
| 1823.857 | 0.91299 |  | 1823.939 | 0.88851 |
| 1825.285 | 0.91315 |  | 1825.367 | 0.88865 |
| 1826.713 | 0.91332 |  | 1826.796 | 0.88876 |
| 1828.141 | 0.91345 |  | 1828.224 | 0.88889 |
| 1829.57 | 0.91352 |  | 1829.652 | 0.88913 |
| 1830.998 | 0.9135 |  | 1831.08 | 0.88945 |
| 1832.426 | 0.91338 |  | 1832.509 | 0.88973 |
| 1833.854 | 0.91322 |  | 1833.937 | 0.88992 |
| 1835.283 | 0.91309 |  | 1835.365 | 0.89003 |
| 1836.711 | 0.91301 |  | 1836.794 | 0.89008 |
| 1838.139 | 0.91294 |  | 1838.222 | 0.89014 |
| 1839.567 | 0.91288 |  | 1839.65 | 0.89024 |
| 1840.995 | 0.91287 |  | 1841.079 | 0.89037 |
| 1842.424 | 0.91298 |  | 1842.507 | 0.89054 |
| 1843.852 | 0.91321 |  | 1843.935 | 0.89071 |
| 1845.28 | 0.91342 |  | 1845.363 | 0.89085 |
| 1846.708 | 0.91346 |  | 1846.792 | 0.89094 |
| 1848.137 | 0.91332 |  | 1848.22 | 0.89095 |
| 1849.565 | 0.9131 |  | 1849.648 | 0.89089 |
| 1850.993 | 0.91288 |  | 1851.077 | 0.8908 |
| 1852.421 | 0.91271 |  | 1852.505 | 0.89073 |
| 1853.85 | 0.91261 |  | 1853.933 | 0.8907 |
| 1855.278 | 0.91256 |  | 1855.362 | 0.89067 |
| 1856.706 | 0.91257 |  | 1856.79 | 0.8906 |
| 1858.134 | 0.91262 |  | 1858.218 | 0.89054 |
| 1859.563 | 0.91269 |  | 1859.646 | 0.89054 |
| 1860.991 | 0.91274 |  | 1861.075 | 0.89058 |
| 1862.419 | 0.91273 |  | 1862.503 | 0.8906 |
| 1863.847 | 0.91268 |  | 1863.931 | 0.89059 |
| 1865.275 | 0.91269 |  | 1865.36 | 0.89055 |
| 1866.704 | 0.91284 |  | 1866.788 | 0.89051 |
| 1868.132 | 0.91312 |  | 1868.216 | 0.89051 |
| 1869.56 | 0.91335 |  | 1869.645 | 0.89056 |
| 1870.988 | 0.91335 |  | 1871.073 | 0.89063 |
| 1872.417 | 0.91314 |  | 1872.501 | 0.89067 |
| 1873.845 | 0.91287 |  | 1873.929 | 0.89068 |
| 1875.273 | 0.91265 |  | 1875.358 | 0.89064 |
| 1876.701 | 0.91253 |  | 1876.786 | 0.89056 |
| 1878.13 | 0.91247 |  | 1878.214 | 0.89044 |
| 1879.558 | 0.91245 |  | 1879.643 | 0.89032 |
| 1880.986 | 0.91245 |  | 1881.071 | 0.89024 |
| 1882.414 | 0.91246 |  | 1882.499 | 0.89021 |
| 1883.843 | 0.91244 |  | 1883.928 | 0.89021 |
| 1885.271 | 0.91239 |  | 1885.356 | 0.89019 |
| 1886.699 | 0.91236 |  | 1886.784 | 0.89016 |
| 1888.127 | 0.91239 |  | 1888.212 | 0.89014 |
| 1889.555 | 0.91246 |  | 1889.641 | 0.89015 |
| 1890.984 | 0.91252 |  | 1891.069 | 0.8902 |
| 1892.412 | 0.91254 |  | 1892.497 | 0.89022 |
| 1893.84 | 0.91254 |  | 1893.926 | 0.89019 |
| 1895.268 | 0.91253 |  | 1895.354 | 0.89009 |
| 1896.697 | 0.9125 |  | 1896.782 | 0.88998 |
| 1898.125 | 0.91243 |  | 1898.211 | 0.8899 |
| 1899.553 | 0.91232 |  | 1899.639 | 0.88985 |
| 1900.981 | 0.9122 |  | 1901.067 | 0.88975 |
| 1902.41 | 0.91208 |  | 1902.495 | 0.88961 |
| 1903.838 | 0.91201 |  | 1903.924 | 0.88951 |
| 1905.266 | 0.912 |  | 1905.352 | 0.8895 |
| 1906.694 | 0.91206 |  | 1906.78 | 0.88955 |
| 1908.123 | 0.91218 |  | 1908.209 | 0.88959 |
| 1909.551 | 0.91229 |  | 1909.637 | 0.88955 |
| 1910.979 | 0.91234 |  | 1911.065 | 0.88943 |
| 1912.407 | 0.91231 |  | 1912.494 | 0.88926 |
| 1913.835 | 0.91222 |  | 1913.922 | 0.88908 |
| 1915.264 | 0.91215 |  | 1915.35 | 0.88891 |
| 1916.692 | 0.91215 |  | 1916.778 | 0.88878 |
| 1918.12 | 0.9122 |  | 1918.207 | 0.88872 |
| 1919.548 | 0.91229 |  | 1919.635 | 0.88873 |
| 1920.977 | 0.91238 |  | 1921.063 | 0.88882 |
| 1922.405 | 0.91245 |  | 1922.492 | 0.88899 |
| 1923.833 | 0.9125 |  | 1923.92 | 0.88912 |
| 1925.261 | 0.91249 |  | 1925.348 | 0.88912 |
| 1926.69 | 0.91242 |  | 1926.777 | 0.889 |
| 1928.118 | 0.91232 |  | 1928.205 | 0.88887 |
| 1929.546 | 0.91222 |  | 1929.633 | 0.88878 |
| 1930.974 | 0.91217 |  | 1931.061 | 0.88872 |
| 1932.403 | 0.91218 |  | 1932.49 | 0.88866 |
| 1933.831 | 0.91222 |  | 1933.918 | 0.8886 |
| 1935.259 | 0.91227 |  | 1935.346 | 0.88859 |
| 1936.687 | 0.91232 |  | 1936.775 | 0.88868 |
| 1938.115 | 0.9124 |  | 1938.203 | 0.88879 |
| 1939.544 | 0.91251 |  | 1939.631 | 0.88887 |
| 1940.972 | 0.91265 |  | 1941.06 | 0.88886 |
| 1942.4 | 0.91277 |  | 1942.488 | 0.88876 |
| 1943.828 | 0.91283 |  | 1943.916 | 0.88862 |
| 1945.257 | 0.91285 |  | 1945.344 | 0.8885 |
| 1946.685 | 0.91284 |  | 1946.773 | 0.88845 |
| 1948.113 | 0.9128 |  | 1948.201 | 0.88847 |
| 1949.541 | 0.91273 |  | 1949.629 | 0.88854 |
| 1950.97 | 0.91265 |  | 1951.058 | 0.88863 |
| 1952.398 | 0.91265 |  | 1952.486 | 0.88879 |
| 1953.826 | 0.91276 |  | 1953.914 | 0.889 |
| 1955.254 | 0.91297 |  | 1955.343 | 0.88923 |
| 1956.683 | 0.91322 |  | 1956.771 | 0.88939 |
| 1958.111 | 0.91341 |  | 1958.199 | 0.88941 |
| 1959.539 | 0.91351 |  | 1959.627 | 0.88935 |
| 1960.967 | 0.91353 |  | 1961.056 | 0.88931 |
| 1962.395 | 0.9135 |  | 1962.484 | 0.88934 |
| 1963.824 | 0.91348 |  | 1963.912 | 0.88943 |
| 1965.252 | 0.91348 |  | 1965.341 | 0.88954 |
| 1966.68 | 0.91351 |  | 1966.769 | 0.8896 |
| 1968.108 | 0.91355 |  | 1968.197 | 0.88963 |
| 1969.537 | 0.91357 |  | 1969.626 | 0.88971 |
| 1970.965 | 0.91355 |  | 1971.054 | 0.8899 |
| 1972.393 | 0.9135 |  | 1972.482 | 0.8901 |
| 1973.821 | 0.91336 |  | 1973.91 | 0.89003 |
| 1975.25 | 0.91304 |  | 1975.339 | 0.8894 |
| 1976.678 | 0.91246 |  | 1976.767 | 0.8881 |
| 1978.106 | 0.91166 |  | 1978.195 | 0.88647 |
| 1979.534 | 0.91088 |  | 1979.624 | 0.88519 |
| 1980.963 | 0.91038 |  | 1981.052 | 0.88467 |
| 1982.391 | 0.91022 |  | 1982.48 | 0.88478 |
| 1983.819 | 0.91029 |  | 1983.909 | 0.88511 |
| 1985.247 | 0.91044 |  | 1985.337 | 0.88542 |
| 1986.675 | 0.91061 |  | 1986.765 | 0.88563 |
| 1988.104 | 0.91075 |  | 1988.193 | 0.88571 |
| 1989.532 | 0.91089 |  | 1989.622 | 0.88571 |
| 1990.96 | 0.91102 |  | 1991.05 | 0.88566 |
| 1992.388 | 0.91116 |  | 1992.478 | 0.88562 |
| 1993.817 | 0.91132 |  | 1993.907 | 0.88561 |
| 1995.245 | 0.91144 |  | 1995.335 | 0.88561 |
| 1996.673 | 0.9115 |  | 1996.763 | 0.88563 |
| 1998.101 | 0.9115 |  | 1998.192 | 0.88563 |
| 1999.53 | 0.91147 |  | 1999.62 | 0.88568 |
| 2000.958 | 0.9115 |  | 2001.048 | 0.88589 |
| 2002.386 | 0.91163 |  | 2002.476 | 0.88629 |
| 2003.814 | 0.91181 |  | 2003.905 | 0.88676 |
| 2005.243 | 0.91194 |  | 2005.333 | 0.88704 |
| 2006.671 | 0.9119 |  | 2006.761 | 0.88695 |
| 2008.099 | 0.91169 |  | 2008.19 | 0.8866 |
| 2009.527 | 0.91138 |  | 2009.618 | 0.88626 |
| 2010.955 | 0.91106 |  | 2011.046 | 0.88602 |
| 2012.384 | 0.91078 |  | 2012.475 | 0.8859 |
| 2013.812 | 0.91062 |  | 2013.903 | 0.88594 |
| 2015.24 | 0.91064 |  | 2015.331 | 0.88616 |
| 2016.668 | 0.91081 |  | 2016.759 | 0.88638 |
| 2018.097 | 0.91102 |  | 2018.188 | 0.88644 |
| 2019.525 | 0.91116 |  | 2019.616 | 0.88639 |
| 2020.953 | 0.91118 |  | 2021.044 | 0.88636 |
| 2022.381 | 0.91116 |  | 2022.473 | 0.88641 |
| 2023.81 | 0.91113 |  | 2023.901 | 0.88657 |
| 2025.238 | 0.91108 |  | 2025.329 | 0.88688 |
| 2026.666 | 0.91102 |  | 2026.758 | 0.88713 |
| 2028.094 | 0.91097 |  | 2028.186 | 0.88709 |
| 2029.523 | 0.91091 |  | 2029.614 | 0.88678 |
| 2030.951 | 0.91078 |  | 2031.042 | 0.88647 |
| 2032.379 | 0.9105 |  | 2032.471 | 0.88627 |
| 2033.807 | 0.91008 |  | 2033.899 | 0.88598 |
| 2035.235 | 0.90961 |  | 2035.327 | 0.88539 |
| 2036.664 | 0.90921 |  | 2036.756 | 0.88462 |
| 2038.092 | 0.90892 |  | 2038.184 | 0.884 |
| 2039.52 | 0.90872 |  | 2039.612 | 0.88374 |
| 2040.948 | 0.90855 |  | 2041.041 | 0.88371 |
| 2042.377 | 0.90842 |  | 2042.469 | 0.88366 |
| 2043.805 | 0.9083 |  | 2043.897 | 0.88353 |
| 2045.233 | 0.90816 |  | 2045.325 | 0.88338 |
| 2046.661 | 0.90791 |  | 2046.754 | 0.88312 |
| 2048.09 | 0.90754 |  | 2048.182 | 0.88252 |
| 2049.518 | 0.90717 |  | 2049.61 | 0.88178 |
| 2050.946 | 0.90698 |  | 2051.039 | 0.88141 |
| 2052.374 | 0.90701 |  | 2052.467 | 0.88162 |
| 2053.803 | 0.90715 |  | 2053.895 | 0.88203 |
| 2055.231 | 0.90727 |  | 2055.324 | 0.88226 |
| 2056.659 | 0.90728 |  | 2056.752 | 0.88235 |
| 2058.087 | 0.90722 |  | 2058.18 | 0.88246 |
| 2059.515 | 0.90715 |  | 2059.608 | 0.88257 |
| 2060.944 | 0.90709 |  | 2061.037 | 0.88259 |
| 2062.372 | 0.90704 |  | 2062.465 | 0.88255 |
| 2063.8 | 0.90699 |  | 2063.893 | 0.88257 |
| 2065.228 | 0.90691 |  | 2065.322 | 0.88264 |
| 2066.657 | 0.90677 |  | 2066.75 | 0.88274 |
| 2068.085 | 0.90659 |  | 2068.178 | 0.88284 |
| 2069.513 | 0.90639 |  | 2069.607 | 0.88292 |
| 2070.941 | 0.90621 |  | 2071.035 | 0.88295 |
| 2072.37 | 0.90606 |  | 2072.463 | 0.88285 |
| 2073.798 | 0.90591 |  | 2073.891 | 0.88265 |
| 2075.226 | 0.90573 |  | 2075.32 | 0.88237 |
| 2076.654 | 0.90555 |  | 2076.748 | 0.88207 |
| 2078.083 | 0.90541 |  | 2078.176 | 0.88184 |
| 2079.511 | 0.90535 |  | 2079.605 | 0.88176 |
| 2080.939 | 0.90537 |  | 2081.033 | 0.88182 |
| 2082.367 | 0.90544 |  | 2082.461 | 0.88196 |
| 2083.795 | 0.90551 |  | 2083.89 | 0.88211 |
| 2085.224 | 0.90554 |  | 2085.318 | 0.88224 |
| 2086.652 | 0.90552 |  | 2086.746 | 0.88231 |
| 2088.08 | 0.90544 |  | 2088.174 | 0.88236 |
| 2089.508 | 0.90537 |  | 2089.603 | 0.88244 |
| 2090.937 | 0.90536 |  | 2091.031 | 0.88263 |
| 2092.365 | 0.90543 |  | 2092.459 | 0.88287 |
| 2093.793 | 0.90553 |  | 2093.888 | 0.88303 |
| 2095.221 | 0.90558 |  | 2095.316 | 0.88296 |
| 2096.65 | 0.9055 |  | 2096.744 | 0.88268 |
| 2098.078 | 0.90532 |  | 2098.173 | 0.88237 |
| 2099.506 | 0.90511 |  | 2099.601 | 0.88219 |
| 2100.934 | 0.90491 |  | 2101.029 | 0.88216 |
| 2102.363 | 0.90476 |  | 2102.457 | 0.88221 |
| 2103.791 | 0.90465 |  | 2103.886 | 0.88223 |
| 2105.219 | 0.90457 |  | 2105.314 | 0.88219 |
| 2106.647 | 0.90452 |  | 2106.742 | 0.88208 |
| 2108.075 | 0.90451 |  | 2108.171 | 0.882 |
| 2109.504 | 0.90451 |  | 2109.599 | 0.88198 |
| 2110.932 | 0.90448 |  | 2111.027 | 0.88199 |
| 2112.36 | 0.90441 |  | 2112.456 | 0.88197 |
| 2113.788 | 0.90432 |  | 2113.884 | 0.88194 |
| 2115.217 | 0.90428 |  | 2115.312 | 0.88199 |
| 2116.645 | 0.9043 |  | 2116.74 | 0.88216 |
| 2118.073 | 0.90439 |  | 2118.169 | 0.88243 |
| 2119.501 | 0.90451 |  | 2119.597 | 0.8827 |
| 2120.93 | 0.90466 |  | 2121.025 | 0.8829 |
| 2122.358 | 0.90482 |  | 2122.454 | 0.88305 |
| 2123.786 | 0.90499 |  | 2123.882 | 0.88317 |
| 2125.214 | 0.90519 |  | 2125.31 | 0.88334 |
| 2126.643 | 0.90542 |  | 2126.739 | 0.88365 |
| 2128.071 | 0.9057 |  | 2128.167 | 0.88408 |
| 2129.499 | 0.90604 |  | 2129.595 | 0.88456 |
| 2130.927 | 0.9064 |  | 2131.023 | 0.88505 |
| 2132.355 | 0.90675 |  | 2132.452 | 0.88551 |
| 2133.784 | 0.907 |  | 2133.88 | 0.88584 |
| 2135.212 | 0.9071 |  | 2135.308 | 0.88598 |
| 2136.64 | 0.90707 |  | 2136.737 | 0.88599 |
| 2138.068 | 0.90696 |  | 2138.165 | 0.88596 |
| 2139.497 | 0.90683 |  | 2139.593 | 0.88592 |
| 2140.925 | 0.9067 |  | 2141.022 | 0.88592 |
| 2142.353 | 0.90656 |  | 2142.45 | 0.88618 |
| 2143.781 | 0.90645 |  | 2143.878 | 0.88669 |
| 2145.21 | 0.90643 |  | 2145.306 | 0.88717 |
| 2146.638 | 0.90655 |  | 2146.735 | 0.88735 |
| 2148.066 | 0.90681 |  | 2148.163 | 0.88734 |
| 2149.494 | 0.90717 |  | 2149.591 | 0.88748 |
| 2150.923 | 0.90761 |  | 2151.02 | 0.88798 |
| 2152.351 | 0.90816 |  | 2152.448 | 0.88881 |
| 2153.779 | 0.90878 |  | 2153.876 | 0.8899 |
| 2155.207 | 0.90934 |  | 2155.305 | 0.89114 |
| 2156.635 | 0.90951 |  | 2156.733 | 0.89209 |
| 2158.064 | 0.90894 |  | 2158.161 | 0.8919 |
| 2159.492 | 0.90763 |  | 2159.589 | 0.8901 |
| 2160.92 | 0.90608 |  | 2161.018 | 0.88752 |
| 2162.348 | 0.90489 |  | 2162.446 | 0.88547 |
| 2163.777 | 0.90428 |  | 2163.874 | 0.88433 |
| 2165.205 | 0.90407 |  | 2165.303 | 0.88372 |
| 2166.633 | 0.904 |  | 2166.731 | 0.88347 |
| 2168.061 | 0.90399 |  | 2168.159 | 0.88366 |
| 2169.49 | 0.90407 |  | 2169.588 | 0.88419 |
| 2170.918 | 0.90425 |  | 2171.016 | 0.88469 |
| 2172.346 | 0.9044 |  | 2172.444 | 0.88492 |
| 2173.774 | 0.90437 |  | 2173.872 | 0.88488 |
| 2175.203 | 0.90412 |  | 2175.301 | 0.88471 |
| 2176.631 | 0.9038 |  | 2176.729 | 0.88458 |
| 2178.059 | 0.90356 |  | 2178.157 | 0.88456 |
| 2179.487 | 0.90346 |  | 2179.586 | 0.88456 |
| 2180.915 | 0.90342 |  | 2181.014 | 0.88437 |
| 2182.344 | 0.90332 |  | 2182.442 | 0.88395 |
| 2183.772 | 0.90314 |  | 2183.871 | 0.88343 |
| 2185.2 | 0.90293 |  | 2185.299 | 0.883 |
| 2186.628 | 0.90279 |  | 2186.727 | 0.8827 |
| 2188.057 | 0.9027 |  | 2188.155 | 0.88247 |
| 2189.485 | 0.90263 |  | 2189.584 | 0.88227 |
| 2190.913 | 0.90255 |  | 2191.012 | 0.88209 |
| 2192.341 | 0.90249 |  | 2192.44 | 0.88198 |
| 2193.77 | 0.90248 |  | 2193.869 | 0.88201 |
| 2195.198 | 0.90247 |  | 2195.297 | 0.88211 |
| 2196.626 | 0.90242 |  | 2196.725 | 0.88212 |
| 2198.054 | 0.90233 |  | 2198.154 | 0.88197 |
| 2199.483 | 0.9022 |  | 2199.582 | 0.88172 |
| 2200.911 | 0.90207 |  | 2201.01 | 0.88148 |
| 2202.339 | 0.90197 |  | 2202.438 | 0.88127 |
| 2203.767 | 0.90191 |  | 2203.867 | 0.88107 |
| 2205.195 | 0.90186 |  | 2205.295 | 0.88089 |
| 2206.624 | 0.90183 |  | 2206.723 | 0.88069 |
| 2208.052 | 0.90184 |  | 2208.152 | 0.88046 |
| 2209.48 | 0.90184 |  | 2209.58 | 0.8802 |
| 2210.908 | 0.90181 |  | 2211.008 | 0.88002 |
| 2212.337 | 0.90171 |  | 2212.437 | 0.87997 |
| 2213.765 | 0.90157 |  | 2213.865 | 0.87999 |
| 2215.193 | 0.90146 |  | 2215.293 | 0.87995 |
| 2216.621 | 0.90139 |  | 2216.721 | 0.8798 |
| 2218.05 | 0.90135 |  | 2218.15 | 0.8796 |
| 2219.478 | 0.90129 |  | 2219.578 | 0.87945 |
| 2220.906 | 0.9012 |  | 2221.006 | 0.87937 |
| 2222.334 | 0.90108 |  | 2222.435 | 0.8793 |
| 2223.763 | 0.90098 |  | 2223.863 | 0.87917 |
| 2225.191 | 0.9009 |  | 2225.291 | 0.87896 |
| 2226.619 | 0.90086 |  | 2226.72 | 0.87872 |
| 2228.047 | 0.90083 |  | 2228.148 | 0.8785 |
| 2229.475 | 0.90078 |  | 2229.576 | 0.87831 |
| 2230.904 | 0.90071 |  | 2231.004 | 0.87815 |
| 2232.332 | 0.90059 |  | 2232.433 | 0.87803 |
| 2233.76 | 0.90045 |  | 2233.861 | 0.87793 |
| 2235.188 | 0.9003 |  | 2235.289 | 0.87778 |
| 2236.617 | 0.90019 |  | 2236.718 | 0.87754 |
| 2238.045 | 0.90013 |  | 2238.146 | 0.87729 |
| 2239.473 | 0.90011 |  | 2239.574 | 0.8771 |
| 2240.901 | 0.90007 |  | 2241.003 | 0.87697 |
| 2242.33 | 0.89999 |  | 2242.431 | 0.87683 |
| 2243.758 | 0.89986 |  | 2243.859 | 0.87662 |
| 2245.186 | 0.89972 |  | 2245.287 | 0.87641 |
| 2246.614 | 0.89958 |  | 2246.716 | 0.87626 |
| 2248.043 | 0.89948 |  | 2248.144 | 0.87621 |
| 2249.471 | 0.89942 |  | 2249.572 | 0.87624 |
| 2250.899 | 0.89939 |  | 2251.001 | 0.87624 |
| 2252.327 | 0.89934 |  | 2252.429 | 0.87615 |
| 2253.755 | 0.89921 |  | 2253.857 | 0.87592 |
| 2255.184 | 0.89897 |  | 2255.286 | 0.87556 |
| 2256.612 | 0.8987 |  | 2256.714 | 0.87517 |
| 2258.04 | 0.89848 |  | 2258.142 | 0.87487 |
| 2259.468 | 0.89835 |  | 2259.57 | 0.87473 |
| 2260.897 | 0.89831 |  | 2260.999 | 0.87469 |
| 2262.325 | 0.89828 |  | 2262.427 | 0.87464 |
| 2263.753 | 0.89824 |  | 2263.855 | 0.87453 |
| 2265.181 | 0.89816 |  | 2265.284 | 0.87431 |
| 2266.61 | 0.89806 |  | 2266.712 | 0.87402 |
| 2268.038 | 0.89792 |  | 2268.14 | 0.87374 |
| 2269.466 | 0.89774 |  | 2269.569 | 0.87352 |
| 2270.894 | 0.89752 |  | 2270.997 | 0.87334 |
| 2272.323 | 0.89729 |  | 2272.425 | 0.87311 |
| 2273.751 | 0.89707 |  | 2273.853 | 0.87281 |
| 2275.179 | 0.8969 |  | 2275.282 | 0.87252 |
| 2276.607 | 0.89678 |  | 2276.71 | 0.87231 |
| 2278.035 | 0.89672 |  | 2278.138 | 0.87217 |
| 2279.464 | 0.89665 |  | 2279.567 | 0.87202 |
| 2280.892 | 0.89654 |  | 2280.995 | 0.87183 |
| 2282.32 | 0.89634 |  | 2282.423 | 0.87158 |
| 2283.748 | 0.89607 |  | 2283.852 | 0.87126 |
| 2285.177 | 0.89574 |  | 2285.28 | 0.87087 |
| 2286.605 | 0.8954 |  | 2286.708 | 0.87051 |
| 2288.033 | 0.89509 |  | 2288.136 | 0.87022 |
| 2289.461 | 0.89484 |  | 2289.565 | 0.87001 |
| 2290.89 | 0.89465 |  | 2290.993 | 0.86981 |
| 2292.318 | 0.89451 |  | 2292.421 | 0.86958 |
| 2293.746 | 0.8944 |  | 2293.85 | 0.86934 |
| 2295.174 | 0.8943 |  | 2295.278 | 0.86911 |
| 2296.603 | 0.8942 |  | 2296.706 | 0.86892 |
| 2298.031 | 0.89409 |  | 2298.135 | 0.86873 |
| 2299.459 | 0.89395 |  | 2299.563 | 0.86847 |
| 2300.887 | 0.89378 |  | 2300.991 | 0.86813 |
| 2302.315 | 0.89356 |  | 2302.419 | 0.86779 |
| 2303.744 | 0.89327 |  | 2303.848 | 0.86749 |
| 2305.172 | 0.89289 |  | 2305.276 | 0.86717 |
| 2306.6 | 0.89244 |  | 2306.704 | 0.86672 |
| 2308.028 | 0.89196 |  | 2308.133 | 0.86618 |
| 2309.457 | 0.8915 |  | 2309.561 | 0.86566 |
| 2310.885 | 0.89108 |  | 2310.989 | 0.86523 |
| 2312.313 | 0.89069 |  | 2312.418 | 0.86488 |
| 2313.741 | 0.89027 |  | 2313.846 | 0.8645 |
| 2315.17 | 0.88979 |  | 2315.274 | 0.86402 |
| 2316.598 | 0.88923 |  | 2316.702 | 0.86337 |
| 2318.026 | 0.88859 |  | 2318.131 | 0.86251 |
| 2319.454 | 0.88788 |  | 2319.559 | 0.86149 |
| 2320.883 | 0.88711 |  | 2320.987 | 0.86041 |
| 2322.311 | 0.88634 |  | 2322.416 | 0.85943 |
| 2323.739 | 0.88574 |  | 2323.844 | 0.85875 |
| 2325.167 | 0.88541 |  | 2325.272 | 0.85849 |
| 2326.595 | 0.88536 |  | 2326.701 | 0.85853 |
| 2328.024 | 0.88549 |  | 2328.129 | 0.85863 |
| 2329.452 | 0.88565 |  | 2329.557 | 0.85858 |
| 2330.88 | 0.88576 |  | 2330.985 | 0.85844 |
| 2332.308 | 0.88579 |  | 2332.414 | 0.85833 |
| 2333.737 | 0.88577 |  | 2333.842 | 0.85822 |
| 2335.165 | 0.88568 |  | 2335.27 | 0.85801 |
| 2336.593 | 0.88553 |  | 2336.699 | 0.85769 |
| 2338.021 | 0.88533 |  | 2338.127 | 0.85741 |
| 2339.45 | 0.88512 |  | 2339.555 | 0.85721 |
| 2340.878 | 0.88503 |  | 2340.984 | 0.8571 |
| 2342.306 | 0.88518 |  | 2342.412 | 0.85712 |
| 2343.734 | 0.88564 |  | 2343.84 | 0.8574 |
| 2345.163 | 0.88622 |  | 2345.268 | 0.85802 |
| 2346.591 | 0.88659 |  | 2346.697 | 0.85871 |
| 2348.019 | 0.88654 |  | 2348.125 | 0.85911 |
| 2349.447 | 0.88606 |  | 2349.553 | 0.85898 |
| 2350.875 | 0.88526 |  | 2350.982 | 0.85835 |
| 2352.304 | 0.8843 |  | 2352.41 | 0.85737 |
| 2353.732 | 0.88337 |  | 2353.838 | 0.85627 |
| 2355.16 | 0.88261 |  | 2355.267 | 0.85522 |
| 2356.588 | 0.88208 |  | 2356.695 | 0.85434 |
| 2358.017 | 0.88178 |  | 2358.123 | 0.8537 |
| 2359.445 | 0.88173 |  | 2359.551 | 0.85333 |
| 2360.873 | 0.88194 |  | 2360.98 | 0.85327 |
| 2362.301 | 0.8824 |  | 2362.408 | 0.85359 |
| 2363.73 | 0.88308 |  | 2363.836 | 0.85423 |
| 2365.158 | 0.8839 |  | 2365.265 | 0.85505 |
| 2366.586 | 0.88477 |  | 2366.693 | 0.85591 |
| 2368.014 | 0.88562 |  | 2368.121 | 0.85681 |
| 2369.443 | 0.88643 |  | 2369.55 | 0.85773 |
| 2370.871 | 0.88723 |  | 2370.978 | 0.85862 |
| 2372.299 | 0.88801 |  | 2372.406 | 0.85941 |
| 2373.727 | 0.88877 |  | 2373.834 | 0.86016 |
| 2375.155 | 0.88949 |  | 2375.263 | 0.86093 |
| 2376.584 | 0.89014 |  | 2376.691 | 0.86173 |
| 2378.012 | 0.89073 |  | 2378.119 | 0.86246 |
| 2379.44 | 0.89125 |  | 2379.548 | 0.86306 |
| 2380.868 | 0.89171 |  | 2380.976 | 0.86357 |
| 2382.297 | 0.89209 |  | 2382.404 | 0.86402 |
| 2383.725 | 0.89241 |  | 2383.833 | 0.8644 |
| 2385.153 | 0.89264 |  | 2385.261 | 0.8647 |
| 2386.581 | 0.89279 |  | 2386.689 | 0.86491 |
| 2388.01 | 0.89289 |  | 2388.117 | 0.86505 |
| 2389.438 | 0.89293 |  | 2389.546 | 0.86513 |
| 2390.866 | 0.89295 |  | 2390.974 | 0.86516 |
| 2392.294 | 0.89294 |  | 2392.402 | 0.86516 |
| 2393.723 | 0.89292 |  | 2393.831 | 0.86511 |
| 2395.151 | 0.8929 |  | 2395.259 | 0.86502 |
| 2396.579 | 0.8929 |  | 2396.687 | 0.86493 |
| 2398.007 | 0.89292 |  | 2398.116 | 0.86489 |
| 2399.435 | 0.89294 |  | 2399.544 | 0.86487 |
| 2400.864 | 0.89293 |  | 2400.972 | 0.86485 |
| 2402.292 | 0.8929 |  | 2402.4 | 0.86478 |
| 2403.72 | 0.89284 |  | 2403.829 | 0.86468 |
| 2405.148 | 0.89277 |  | 2405.257 | 0.86459 |
| 2406.577 | 0.8927 |  | 2406.685 | 0.86449 |
| 2408.005 | 0.89261 |  | 2408.114 | 0.86437 |
| 2409.433 | 0.89252 |  | 2409.542 | 0.86422 |
| 2410.861 | 0.89242 |  | 2410.97 | 0.86408 |
| 2412.29 | 0.89234 |  | 2412.399 | 0.86398 |
| 2413.718 | 0.89229 |  | 2413.827 | 0.86391 |
| 2415.146 | 0.89228 |  | 2415.255 | 0.86385 |
| 2416.574 | 0.89231 |  | 2416.683 | 0.8638 |
| 2418.003 | 0.89234 |  | 2418.112 | 0.86378 |
| 2419.431 | 0.89235 |  | 2419.54 | 0.86376 |
| 2420.859 | 0.89232 |  | 2420.968 | 0.8637 |
| 2422.287 | 0.89226 |  | 2422.397 | 0.86359 |
| 2423.715 | 0.89216 |  | 2423.825 | 0.86347 |
| 2425.144 | 0.89207 |  | 2425.253 | 0.86337 |
| 2426.572 | 0.89197 |  | 2426.682 | 0.86327 |
| 2428 | 0.89188 |  | 2428.11 | 0.86316 |
| 2429.428 | 0.89181 |  | 2429.538 | 0.86304 |
| 2430.857 | 0.89175 |  | 2430.966 | 0.86294 |
| 2432.285 | 0.89168 |  | 2432.395 | 0.86285 |
| 2433.713 | 0.8916 |  | 2433.823 | 0.86274 |
| 2435.141 | 0.89152 |  | 2435.251 | 0.86263 |
| 2436.57 | 0.89143 |  | 2436.68 | 0.86251 |
| 2437.998 | 0.89136 |  | 2438.108 | 0.86238 |
| 2439.426 | 0.89129 |  | 2439.536 | 0.86226 |
| 2440.854 | 0.89123 |  | 2440.965 | 0.86213 |
| 2442.283 | 0.89118 |  | 2442.393 | 0.86199 |
| 2443.711 | 0.89112 |  | 2443.821 | 0.86185 |
| 2445.139 | 0.89107 |  | 2445.249 | 0.86174 |
| 2446.567 | 0.89102 |  | 2446.678 | 0.86166 |
| 2447.996 | 0.89095 |  | 2448.106 | 0.86158 |
| 2449.424 | 0.89088 |  | 2449.534 | 0.86149 |
| 2450.852 | 0.89079 |  | 2450.963 | 0.86139 |
| 2452.28 | 0.89071 |  | 2452.391 | 0.86129 |
| 2453.708 | 0.89062 |  | 2453.819 | 0.86117 |
| 2455.137 | 0.89054 |  | 2455.248 | 0.86103 |
| 2456.565 | 0.89045 |  | 2456.676 | 0.8609 |
| 2457.993 | 0.89037 |  | 2458.104 | 0.86081 |
| 2459.421 | 0.89029 |  | 2459.532 | 0.86077 |
| 2460.85 | 0.89022 |  | 2460.961 | 0.86077 |
| 2462.278 | 0.89015 |  | 2462.389 | 0.86076 |
| 2463.706 | 0.8901 |  | 2463.817 | 0.86072 |
| 2465.134 | 0.89007 |  | 2465.246 | 0.86063 |
| 2466.563 | 0.89007 |  | 2466.674 | 0.86054 |
| 2467.991 | 0.89007 |  | 2468.102 | 0.86043 |
| 2469.419 | 0.89005 |  | 2469.531 | 0.86031 |
| 2470.847 | 0.89002 |  | 2470.959 | 0.86018 |
| 2472.276 | 0.88996 |  | 2472.387 | 0.86005 |
| 2473.704 | 0.88991 |  | 2473.815 | 0.85994 |
| 2475.132 | 0.88987 |  | 2475.244 | 0.85984 |
| 2476.56 | 0.88984 |  | 2476.672 | 0.85977 |
| 2477.988 | 0.88981 |  | 2478.1 | 0.8597 |
| 2479.417 | 0.88976 |  | 2479.529 | 0.85962 |
| 2480.845 | 0.8897 |  | 2480.957 | 0.85954 |
| 2482.273 | 0.88963 |  | 2482.385 | 0.85946 |
| 2483.701 | 0.88957 |  | 2483.814 | 0.85935 |
| 2485.13 | 0.88949 |  | 2485.242 | 0.85923 |
| 2486.558 | 0.8894 |  | 2486.67 | 0.85911 |
| 2487.986 | 0.8893 |  | 2488.098 | 0.85896 |
| 2489.414 | 0.88918 |  | 2489.527 | 0.85878 |
| 2490.843 | 0.88907 |  | 2490.955 | 0.85859 |
| 2492.271 | 0.88899 |  | 2492.383 | 0.85844 |
| 2493.699 | 0.88892 |  | 2493.812 | 0.85836 |
| 2495.127 | 0.88884 |  | 2495.24 | 0.85831 |
| 2496.556 | 0.88876 |  | 2496.668 | 0.85827 |
| 2497.984 | 0.88869 |  | 2498.097 | 0.85817 |
| 2499.412 | 0.88863 |  | 2499.525 | 0.85803 |
| 2500.84 | 0.8886 |  | 2500.953 | 0.85787 |
| 2502.268 | 0.88857 |  | 2502.381 | 0.8577 |
| 2503.697 | 0.88852 |  | 2503.81 | 0.85751 |
| 2505.125 | 0.88845 |  | 2505.238 | 0.85733 |
| 2506.553 | 0.88837 |  | 2506.666 | 0.85722 |
| 2507.981 | 0.88831 |  | 2508.095 | 0.85716 |
| 2509.41 | 0.88823 |  | 2509.523 | 0.85711 |
| 2510.838 | 0.88814 |  | 2510.951 | 0.85705 |
| 2512.266 | 0.88805 |  | 2512.38 | 0.85699 |
| 2513.694 | 0.88798 |  | 2513.808 | 0.85692 |
| 2515.123 | 0.88794 |  | 2515.236 | 0.85683 |
| 2516.551 | 0.88791 |  | 2516.664 | 0.85672 |
| 2517.979 | 0.88786 |  | 2518.093 | 0.85658 |
| 2519.407 | 0.88779 |  | 2519.521 | 0.85643 |
| 2520.836 | 0.88771 |  | 2520.949 | 0.85628 |
| 2522.264 | 0.88764 |  | 2522.378 | 0.85614 |
| 2523.692 | 0.88759 |  | 2523.806 | 0.85604 |
| 2525.12 | 0.88754 |  | 2525.234 | 0.85594 |
| 2526.548 | 0.88749 |  | 2526.663 | 0.85582 |
| 2527.977 | 0.88742 |  | 2528.091 | 0.85566 |
| 2529.405 | 0.88733 |  | 2529.519 | 0.8555 |
| 2530.833 | 0.88722 |  | 2530.947 | 0.85536 |
| 2532.261 | 0.88709 |  | 2532.376 | 0.85523 |
| 2533.69 | 0.88697 |  | 2533.804 | 0.85511 |
| 2535.118 | 0.88686 |  | 2535.232 | 0.85501 |
| 2536.546 | 0.88678 |  | 2536.661 | 0.85489 |
| 2537.974 | 0.88674 |  | 2538.089 | 0.85475 |
| 2539.403 | 0.8867 |  | 2539.517 | 0.85459 |
| 2540.831 | 0.88666 |  | 2540.946 | 0.85444 |
| 2542.259 | 0.88658 |  | 2542.374 | 0.8543 |
| 2543.687 | 0.88649 |  | 2543.802 | 0.85416 |
| 2545.116 | 0.8864 |  | 2545.23 | 0.85404 |
| 2546.544 | 0.8863 |  | 2546.659 | 0.85393 |
| 2547.972 | 0.88621 |  | 2548.087 | 0.85378 |
| 2549.4 | 0.88612 |  | 2549.515 | 0.85357 |
| 2550.828 | 0.88604 |  | 2550.944 | 0.85336 |
| 2552.257 | 0.88595 |  | 2552.372 | 0.8532 |
| 2553.685 | 0.88588 |  | 2553.8 | 0.85309 |
| 2555.113 | 0.88581 |  | 2555.229 | 0.853 |
| 2556.541 | 0.88574 |  | 2556.657 | 0.8529 |
| 2557.97 | 0.88566 |  | 2558.085 | 0.85279 |
| 2559.398 | 0.88554 |  | 2559.513 | 0.85266 |
| 2560.826 | 0.8854 |  | 2560.942 | 0.85251 |
| 2562.254 | 0.88525 |  | 2562.37 | 0.85232 |
| 2563.683 | 0.88513 |  | 2563.798 | 0.8521 |
| 2565.111 | 0.88502 |  | 2565.227 | 0.85187 |
| 2566.539 | 0.88494 |  | 2566.655 | 0.85166 |
| 2567.967 | 0.88486 |  | 2568.083 | 0.8515 |
| 2569.396 | 0.88478 |  | 2569.512 | 0.85136 |
| 2570.824 | 0.88469 |  | 2570.94 | 0.85122 |
| 2572.252 | 0.88459 |  | 2572.368 | 0.85104 |
| 2573.68 | 0.88449 |  | 2573.796 | 0.85081 |
| 2575.108 | 0.8844 |  | 2575.225 | 0.85058 |
| 2576.537 | 0.88431 |  | 2576.653 | 0.85037 |
| 2577.965 | 0.88422 |  | 2578.081 | 0.85018 |
| 2579.393 | 0.8841 |  | 2579.51 | 0.84999 |
| 2580.821 | 0.88398 |  | 2580.938 | 0.84977 |
| 2582.25 | 0.88384 |  | 2582.366 | 0.84957 |
| 2583.678 | 0.88373 |  | 2583.795 | 0.84939 |
| 2585.106 | 0.88362 |  | 2585.223 | 0.84921 |
| 2586.534 | 0.88353 |  | 2586.651 | 0.84899 |
| 2587.963 | 0.88342 |  | 2588.079 | 0.84875 |
| 2589.391 | 0.8833 |  | 2589.508 | 0.84854 |
| 2590.819 | 0.88316 |  | 2590.936 | 0.84835 |
| 2592.247 | 0.88302 |  | 2592.364 | 0.84816 |
| 2593.676 | 0.8829 |  | 2593.793 | 0.84797 |
| 2595.104 | 0.8828 |  | 2595.221 | 0.8478 |
| 2596.532 | 0.8827 |  | 2596.649 | 0.84764 |
| 2597.96 | 0.88258 |  | 2598.078 | 0.84743 |
| 2599.388 | 0.88245 |  | 2599.506 | 0.84715 |
| 2600.817 | 0.88231 |  | 2600.934 | 0.84685 |
| 2602.245 | 0.88219 |  | 2602.362 | 0.84658 |
| 2603.673 | 0.88208 |  | 2603.791 | 0.84634 |
| 2605.101 | 0.88197 |  | 2605.219 | 0.8461 |
| 2606.53 | 0.88187 |  | 2606.647 | 0.84585 |
| 2607.958 | 0.88176 |  | 2608.076 | 0.8456 |
| 2609.386 | 0.88162 |  | 2609.504 | 0.84533 |
| 2610.814 | 0.88148 |  | 2610.932 | 0.84506 |
| 2612.243 | 0.88131 |  | 2612.361 | 0.84477 |
| 2613.671 | 0.88114 |  | 2613.789 | 0.84447 |
| 2615.099 | 0.88098 |  | 2615.217 | 0.84418 |
| 2616.527 | 0.88083 |  | 2616.645 | 0.84393 |
| 2617.956 | 0.88067 |  | 2618.074 | 0.84369 |
| 2619.384 | 0.88051 |  | 2619.502 | 0.84345 |
| 2620.812 | 0.88034 |  | 2620.93 | 0.84318 |
| 2622.24 | 0.88015 |  | 2622.359 | 0.84285 |
| 2623.668 | 0.87998 |  | 2623.787 | 0.84247 |
| 2625.097 | 0.87982 |  | 2625.215 | 0.84209 |
| 2626.525 | 0.87966 |  | 2626.644 | 0.84176 |
| 2627.953 | 0.8795 |  | 2628.072 | 0.84149 |
| 2629.381 | 0.87932 |  | 2629.5 | 0.84122 |
| 2630.81 | 0.87911 |  | 2630.928 | 0.84095 |
| 2632.238 | 0.8789 |  | 2632.357 | 0.84066 |
| 2633.666 | 0.87869 |  | 2633.785 | 0.84037 |
| 2635.094 | 0.87848 |  | 2635.213 | 0.84006 |
| 2636.523 | 0.87829 |  | 2636.642 | 0.83971 |
| 2637.951 | 0.87812 |  | 2638.07 | 0.83936 |
| 2639.379 | 0.87795 |  | 2639.498 | 0.83902 |
| 2640.807 | 0.87777 |  | 2640.927 | 0.83872 |
| 2642.236 | 0.87757 |  | 2642.355 | 0.83842 |
| 2643.664 | 0.87735 |  | 2643.783 | 0.8381 |
| 2645.092 | 0.87712 |  | 2645.211 | 0.83777 |
| 2646.52 | 0.8769 |  | 2646.64 | 0.83747 |
| 2647.948 | 0.87668 |  | 2648.068 | 0.83719 |
| 2649.377 | 0.87648 |  | 2649.496 | 0.83691 |
| 2650.805 | 0.87629 |  | 2650.925 | 0.83662 |
| 2652.233 | 0.8761 |  | 2652.353 | 0.83631 |
| 2653.661 | 0.87591 |  | 2653.781 | 0.83598 |
| 2655.09 | 0.87573 |  | 2655.21 | 0.83566 |
| 2656.518 | 0.87557 |  | 2656.638 | 0.8354 |
| 2657.946 | 0.87541 |  | 2658.066 | 0.83516 |
| 2659.374 | 0.87527 |  | 2659.494 | 0.83492 |
| 2660.803 | 0.87515 |  | 2660.923 | 0.83468 |
| 2662.231 | 0.87507 |  | 2662.351 | 0.83447 |
| 2663.659 | 0.87502 |  | 2663.779 | 0.83431 |
| 2665.087 | 0.87498 |  | 2665.208 | 0.83419 |
| 2666.516 | 0.87495 |  | 2666.636 | 0.83406 |
| 2667.944 | 0.87491 |  | 2668.064 | 0.83391 |
| 2669.372 | 0.87486 |  | 2669.493 | 0.8337 |
| 2670.8 | 0.87479 |  | 2670.921 | 0.83345 |
| 2672.228 | 0.87472 |  | 2672.349 | 0.8332 |
| 2673.657 | 0.87464 |  | 2673.777 | 0.83297 |
| 2675.085 | 0.87455 |  | 2675.206 | 0.83277 |
| 2676.513 | 0.87446 |  | 2676.634 | 0.83258 |
| 2677.941 | 0.87436 |  | 2678.062 | 0.83237 |
| 2679.37 | 0.87427 |  | 2679.491 | 0.83217 |
| 2680.798 | 0.87419 |  | 2680.919 | 0.83201 |
| 2682.226 | 0.87412 |  | 2682.347 | 0.83189 |
| 2683.654 | 0.87406 |  | 2683.776 | 0.83176 |
| 2685.083 | 0.87399 |  | 2685.204 | 0.83157 |
| 2686.511 | 0.87392 |  | 2686.632 | 0.83134 |
| 2687.939 | 0.87384 |  | 2688.06 | 0.8311 |
| 2689.367 | 0.87374 |  | 2689.489 | 0.83086 |
| 2690.796 | 0.87364 |  | 2690.917 | 0.83064 |
| 2692.224 | 0.87355 |  | 2692.345 | 0.83042 |
| 2693.652 | 0.87346 |  | 2693.774 | 0.83018 |
| 2695.08 | 0.87337 |  | 2695.202 | 0.82993 |
| 2696.508 | 0.87328 |  | 2696.63 | 0.82968 |
| 2697.937 | 0.8732 |  | 2698.059 | 0.82941 |
| 2699.365 | 0.87313 |  | 2699.487 | 0.82914 |
| 2700.793 | 0.87306 |  | 2700.915 | 0.82888 |
| 2702.221 | 0.87298 |  | 2702.343 | 0.82865 |
| 2703.65 | 0.87288 |  | 2703.772 | 0.82843 |
| 2705.078 | 0.87276 |  | 2705.2 | 0.82818 |
| 2706.506 | 0.87264 |  | 2706.628 | 0.8279 |
| 2707.934 | 0.87253 |  | 2708.057 | 0.82762 |
| 2709.363 | 0.87245 |  | 2709.485 | 0.82735 |
| 2710.791 | 0.87239 |  | 2710.913 | 0.82711 |
| 2712.219 | 0.87233 |  | 2712.342 | 0.82686 |
| 2713.647 | 0.87225 |  | 2713.77 | 0.82658 |
| 2715.076 | 0.87215 |  | 2715.198 | 0.82628 |
| 2716.504 | 0.87204 |  | 2716.626 | 0.82601 |
| 2717.932 | 0.87193 |  | 2718.055 | 0.82575 |
| 2719.36 | 0.87183 |  | 2719.483 | 0.82544 |
| 2720.788 | 0.87173 |  | 2720.911 | 0.82507 |
| 2722.217 | 0.87164 |  | 2722.34 | 0.82464 |
| 2723.645 | 0.87155 |  | 2723.768 | 0.8242 |
| 2725.073 | 0.87148 |  | 2725.196 | 0.82382 |
| 2726.501 | 0.87141 |  | 2726.625 | 0.82355 |
| 2727.93 | 0.87134 |  | 2728.053 | 0.82339 |
| 2729.358 | 0.87126 |  | 2729.481 | 0.82325 |
| 2730.786 | 0.87118 |  | 2730.909 | 0.82301 |
| 2732.214 | 0.87109 |  | 2732.338 | 0.82268 |
| 2733.643 | 0.87099 |  | 2733.766 | 0.82228 |
| 2735.071 | 0.87089 |  | 2735.194 | 0.82188 |
| 2736.499 | 0.8708 |  | 2736.623 | 0.82151 |
| 2737.927 | 0.87072 |  | 2738.051 | 0.82119 |
| 2739.356 | 0.87066 |  | 2739.479 | 0.8209 |
| 2740.784 | 0.8706 |  | 2740.908 | 0.82058 |
| 2742.212 | 0.87053 |  | 2742.336 | 0.82021 |
| 2743.64 | 0.87042 |  | 2743.764 | 0.81976 |
| 2745.068 | 0.87029 |  | 2745.192 | 0.81926 |
| 2746.497 | 0.87015 |  | 2746.621 | 0.81873 |
| 2747.925 | 0.87003 |  | 2748.049 | 0.81827 |
| 2749.353 | 0.86993 |  | 2749.477 | 0.81792 |
| 2750.781 | 0.86986 |  | 2750.906 | 0.81767 |
| 2752.21 | 0.86979 |  | 2752.334 | 0.81741 |
| 2753.638 | 0.8697 |  | 2753.762 | 0.81707 |
| 2755.066 | 0.86961 |  | 2755.191 | 0.81664 |
| 2756.494 | 0.86952 |  | 2756.619 | 0.81619 |
| 2757.923 | 0.86943 |  | 2758.047 | 0.81575 |
| 2759.351 | 0.86935 |  | 2759.475 | 0.81533 |
| 2760.779 | 0.86925 |  | 2760.904 | 0.81492 |
| 2762.207 | 0.86915 |  | 2762.332 | 0.8145 |
| 2763.636 | 0.86905 |  | 2763.76 | 0.81403 |
| 2765.064 | 0.86895 |  | 2765.189 | 0.81355 |
| 2766.492 | 0.86887 |  | 2766.617 | 0.81307 |
| 2767.92 | 0.86879 |  | 2768.045 | 0.81257 |
| 2769.348 | 0.86871 |  | 2769.474 | 0.812 |
| 2770.777 | 0.86862 |  | 2770.902 | 0.81132 |
| 2772.205 | 0.86851 |  | 2772.33 | 0.81055 |
| 2773.633 | 0.86839 |  | 2773.758 | 0.80981 |
| 2775.061 | 0.86828 |  | 2775.187 | 0.80921 |
| 2776.49 | 0.86818 |  | 2776.615 | 0.80873 |
| 2777.918 | 0.8681 |  | 2778.043 | 0.80827 |
| 2779.346 | 0.86803 |  | 2779.472 | 0.80774 |
| 2780.774 | 0.86794 |  | 2780.9 | 0.80713 |
| 2782.203 | 0.86784 |  | 2782.328 | 0.80647 |
| 2783.631 | 0.86774 |  | 2783.757 | 0.80575 |
| 2785.059 | 0.86765 |  | 2785.185 | 0.80491 |
| 2786.487 | 0.86756 |  | 2786.613 | 0.80395 |
| 2787.916 | 0.86747 |  | 2788.041 | 0.80291 |
| 2789.344 | 0.86736 |  | 2789.47 | 0.80191 |
| 2790.772 | 0.86725 |  | 2790.898 | 0.80102 |
| 2792.2 | 0.86713 |  | 2792.326 | 0.80021 |
| 2793.628 | 0.86702 |  | 2793.755 | 0.79938 |
| 2795.057 | 0.86692 |  | 2795.183 | 0.79849 |
| 2796.485 | 0.86682 |  | 2796.611 | 0.79757 |
| 2797.913 | 0.86671 |  | 2798.04 | 0.79675 |
| 2799.341 | 0.8666 |  | 2799.468 | 0.79613 |
| 2800.77 | 0.86649 |  | 2800.896 | 0.79573 |
| 2802.198 | 0.86637 |  | 2802.324 | 0.79549 |
| 2803.626 | 0.86625 |  | 2803.753 | 0.79533 |
| 2805.054 | 0.86612 |  | 2805.181 | 0.79523 |
| 2806.483 | 0.86599 |  | 2806.609 | 0.79516 |
| 2807.911 | 0.86589 |  | 2808.038 | 0.79513 |
| 2809.339 | 0.86579 |  | 2809.466 | 0.79522 |
| 2810.767 | 0.8657 |  | 2810.894 | 0.79548 |
| 2812.196 | 0.8656 |  | 2812.323 | 0.79583 |
| 2813.624 | 0.86551 |  | 2813.751 | 0.79616 |
| 2815.052 | 0.86541 |  | 2815.179 | 0.79641 |
| 2816.48 | 0.86533 |  | 2816.607 | 0.79657 |
| 2817.908 | 0.86523 |  | 2818.036 | 0.79667 |
| 2819.337 | 0.86512 |  | 2819.464 | 0.79674 |
| 2820.765 | 0.865 |  | 2820.892 | 0.7968 |
| 2822.193 | 0.86487 |  | 2822.321 | 0.79685 |
| 2823.621 | 0.86473 |  | 2823.749 | 0.79691 |
| 2825.05 | 0.8646 |  | 2825.177 | 0.79702 |
| 2826.478 | 0.86448 |  | 2826.606 | 0.79713 |
| 2827.906 | 0.86436 |  | 2828.034 | 0.7972 |
| 2829.334 | 0.86425 |  | 2829.462 | 0.7972 |
| 2830.763 | 0.86415 |  | 2830.89 | 0.79717 |
| 2832.191 | 0.86405 |  | 2832.319 | 0.79714 |
| 2833.619 | 0.86394 |  | 2833.747 | 0.79712 |
| 2835.047 | 0.8638 |  | 2835.175 | 0.79708 |
| 2836.476 | 0.86365 |  | 2836.604 | 0.79702 |
| 2837.904 | 0.8635 |  | 2838.032 | 0.79699 |
| 2839.332 | 0.86336 |  | 2839.46 | 0.79703 |
| 2840.76 | 0.86325 |  | 2840.889 | 0.79717 |
| 2842.188 | 0.86317 |  | 2842.317 | 0.79733 |
| 2843.617 | 0.86312 |  | 2843.745 | 0.79741 |
| 2845.045 | 0.86306 |  | 2845.173 | 0.79736 |
| 2846.473 | 0.863 |  | 2846.602 | 0.79724 |
| 2847.901 | 0.86292 |  | 2848.03 | 0.79716 |
| 2849.33 | 0.86286 |  | 2849.458 | 0.79719 |
| 2850.758 | 0.8628 |  | 2850.887 | 0.79726 |
| 2852.186 | 0.86274 |  | 2852.315 | 0.79731 |
| 2853.614 | 0.86265 |  | 2853.743 | 0.79732 |
| 2855.043 | 0.86254 |  | 2855.172 | 0.79733 |
| 2856.471 | 0.86241 |  | 2856.6 | 0.79733 |
| 2857.899 | 0.86228 |  | 2858.028 | 0.79732 |
| 2859.327 | 0.86215 |  | 2859.456 | 0.79729 |
| 2860.756 | 0.86203 |  | 2860.885 | 0.79728 |
| 2862.184 | 0.8619 |  | 2862.313 | 0.79726 |
| 2863.612 | 0.86176 |  | 2863.741 | 0.7972 |
| 2865.04 | 0.86162 |  | 2865.17 | 0.7971 |
| 2866.468 | 0.86147 |  | 2866.598 | 0.79701 |
| 2867.897 | 0.86131 |  | 2868.026 | 0.79693 |
| 2869.325 | 0.86116 |  | 2869.455 | 0.7968 |
| 2870.753 | 0.86103 |  | 2870.883 | 0.79663 |
| 2872.181 | 0.86092 |  | 2872.311 | 0.79645 |
| 2873.61 | 0.86082 |  | 2873.739 | 0.79631 |
| 2875.038 | 0.86072 |  | 2875.168 | 0.79623 |
| 2876.466 | 0.86061 |  | 2876.596 | 0.79615 |
| 2877.894 | 0.86048 |  | 2878.024 | 0.79601 |
| 2879.323 | 0.86035 |  | 2879.453 | 0.79578 |
| 2880.751 | 0.86021 |  | 2880.881 | 0.79547 |
| 2882.179 | 0.86008 |  | 2882.309 | 0.79514 |
| 2883.607 | 0.85997 |  | 2883.738 | 0.79483 |
| 2885.036 | 0.85987 |  | 2885.166 | 0.79457 |
| 2886.464 | 0.85978 |  | 2886.594 | 0.79433 |
| 2887.892 | 0.85969 |  | 2888.022 | 0.79407 |
| 2889.32 | 0.85958 |  | 2889.451 | 0.79373 |
| 2890.748 | 0.85945 |  | 2890.879 | 0.79332 |
| 2892.177 | 0.85933 |  | 2892.307 | 0.79285 |
| 2893.605 | 0.85922 |  | 2893.736 | 0.79237 |
| 2895.033 | 0.85912 |  | 2895.164 | 0.79188 |
| 2896.461 | 0.85903 |  | 2896.592 | 0.79135 |
| 2897.89 | 0.85893 |  | 2898.021 | 0.79074 |
| 2899.318 | 0.85882 |  | 2899.449 | 0.79008 |
| 2900.746 | 0.8587 |  | 2900.877 | 0.78953 |
| 2902.174 | 0.85858 |  | 2902.305 | 0.78917 |
| 2903.603 | 0.85848 |  | 2903.734 | 0.78891 |
| 2905.031 | 0.8584 |  | 2905.162 | 0.78861 |
| 2906.459 | 0.85833 |  | 2906.59 | 0.78822 |
| 2907.887 | 0.85825 |  | 2908.019 | 0.78776 |
| 2909.316 | 0.85817 |  | 2909.447 | 0.78728 |
| 2910.744 | 0.8581 |  | 2910.875 | 0.78682 |
| 2912.172 | 0.85803 |  | 2912.304 | 0.78643 |
| 2913.6 | 0.85796 |  | 2913.732 | 0.78608 |
| 2915.028 | 0.85788 |  | 2915.16 | 0.78571 |
| 2916.457 | 0.8578 |  | 2916.588 | 0.7853 |
| 2917.885 | 0.85773 |  | 2918.017 | 0.78486 |
| 2919.313 | 0.85768 |  | 2919.445 | 0.78444 |
| 2920.741 | 0.85763 |  | 2920.873 | 0.78406 |
| 2922.17 | 0.85757 |  | 2922.302 | 0.7837 |
| 2923.598 | 0.85748 |  | 2923.73 | 0.78336 |
| 2925.026 | 0.85735 |  | 2925.158 | 0.78305 |
| 2926.454 | 0.8572 |  | 2926.587 | 0.78277 |
| 2927.883 | 0.85707 |  | 2928.015 | 0.78254 |
| 2929.311 | 0.85697 |  | 2929.443 | 0.78232 |
| 2930.739 | 0.8569 |  | 2930.871 | 0.78207 |
| 2932.167 | 0.85685 |  | 2932.3 | 0.78176 |
| 2933.596 | 0.8568 |  | 2933.728 | 0.78141 |
| 2935.024 | 0.85674 |  | 2935.156 | 0.78104 |
| 2936.452 | 0.85667 |  | 2936.585 | 0.78068 |
| 2937.88 | 0.85659 |  | 2938.013 | 0.78032 |
| 2939.308 | 0.8565 |  | 2939.441 | 0.77995 |
| 2940.737 | 0.8564 |  | 2940.87 | 0.77956 |
| 2942.165 | 0.8563 |  | 2942.298 | 0.77919 |
| 2943.593 | 0.8562 |  | 2943.726 | 0.77882 |
| 2945.021 | 0.85609 |  | 2945.154 | 0.77847 |
| 2946.45 | 0.85597 |  | 2946.583 | 0.77809 |
| 2947.878 | 0.85583 |  | 2948.011 | 0.77767 |
| 2949.306 | 0.85568 |  | 2949.439 | 0.77718 |
| 2950.734 | 0.85554 |  | 2950.868 | 0.77663 |
| 2952.163 | 0.85539 |  | 2952.296 | 0.77605 |
| 2953.591 | 0.85525 |  | 2953.724 | 0.77543 |
| 2955.019 | 0.8551 |  | 2955.153 | 0.77482 |
| 2956.447 | 0.85496 |  | 2956.581 | 0.77423 |
| 2957.876 | 0.85481 |  | 2958.009 | 0.7737 |
| 2959.304 | 0.85464 |  | 2959.437 | 0.77321 |
| 2960.732 | 0.85446 |  | 2960.866 | 0.77274 |
| 2962.16 | 0.85425 |  | 2962.294 | 0.77224 |
| 2963.588 | 0.85403 |  | 2963.722 | 0.77169 |
| 2965.017 | 0.85379 |  | 2965.151 | 0.7711 |
| 2966.445 | 0.85356 |  | 2966.579 | 0.77048 |
| 2967.873 | 0.85334 |  | 2968.007 | 0.76988 |
| 2969.301 | 0.85312 |  | 2969.436 | 0.76935 |
| 2970.73 | 0.8529 |  | 2970.864 | 0.76894 |
| 2972.158 | 0.85267 |  | 2972.292 | 0.76865 |
| 2973.586 | 0.85244 |  | 2973.72 | 0.7684 |
| 2975.014 | 0.85225 |  | 2975.149 | 0.76812 |
| 2976.443 | 0.85211 |  | 2976.577 | 0.76783 |
| 2977.871 | 0.85201 |  | 2978.005 | 0.76757 |
| 2979.299 | 0.85196 |  | 2979.434 | 0.76734 |
| 2980.727 | 0.85196 |  | 2980.862 | 0.76706 |
| 2982.156 | 0.85199 |  | 2982.29 | 0.7667 |
| 2983.584 | 0.85202 |  | 2983.719 | 0.76625 |
| 2985.012 | 0.85202 |  | 2985.147 | 0.76577 |
| 2986.44 | 0.85199 |  | 2986.575 | 0.76533 |
| 2987.868 | 0.85195 |  | 2988.003 | 0.76505 |
| 2989.297 | 0.85192 |  | 2989.432 | 0.76502 |
| 2990.725 | 0.8519 |  | 2990.86 | 0.7652 |
| 2992.153 | 0.85187 |  | 2992.288 | 0.7654 |
| 2993.581 | 0.85181 |  | 2993.717 | 0.76546 |
| 2995.01 | 0.85171 |  | 2995.145 | 0.7654 |
| 2996.438 | 0.85161 |  | 2996.573 | 0.76531 |
| 2997.866 | 0.85151 |  | 2998.002 | 0.76523 |
| 2999.294 | 0.85142 |  | 2999.43 | 0.76513 |
| 3000.723 | 0.85134 |  | 3000.858 | 0.76505 |
| 3002.151 | 0.85126 |  | 3002.286 | 0.76502 |
| 3003.579 | 0.85116 |  | 3003.715 | 0.76501 |
| 3005.007 | 0.85105 |  | 3005.143 | 0.765 |
| 3006.436 | 0.85091 |  | 3006.571 | 0.76503 |
| 3007.864 | 0.85078 |  | 3008 | 0.76515 |
| 3009.292 | 0.85067 |  | 3009.428 | 0.76533 |
| 3010.72 | 0.85059 |  | 3010.856 | 0.76551 |
| 3012.148 | 0.85054 |  | 3012.285 | 0.76562 |
| 3013.577 | 0.85047 |  | 3013.713 | 0.76568 |
| 3015.005 | 0.85038 |  | 3015.141 | 0.7657 |
| 3016.433 | 0.85026 |  | 3016.569 | 0.76573 |
| 3017.861 | 0.85013 |  | 3017.998 | 0.76578 |
| 3019.29 | 0.85001 |  | 3019.426 | 0.76585 |
| 3020.718 | 0.84989 |  | 3020.854 | 0.76593 |
| 3022.146 | 0.84978 |  | 3022.283 | 0.76603 |
| 3023.574 | 0.84967 |  | 3023.711 | 0.76613 |
| 3025.003 | 0.84954 |  | 3025.139 | 0.76623 |
| 3026.431 | 0.84942 |  | 3026.568 | 0.76634 |
| 3027.859 | 0.84931 |  | 3027.996 | 0.76651 |
| 3029.287 | 0.84924 |  | 3029.424 | 0.76675 |
| 3030.716 | 0.8492 |  | 3030.852 | 0.76704 |
| 3032.144 | 0.84915 |  | 3032.281 | 0.76736 |
| 3033.572 | 0.84905 |  | 3033.709 | 0.76768 |
| 3035 | 0.8489 |  | 3035.137 | 0.76798 |
| 3036.428 | 0.8487 |  | 3036.566 | 0.76829 |
| 3037.857 | 0.8485 |  | 3037.994 | 0.76868 |
| 3039.285 | 0.8483 |  | 3039.422 | 0.76918 |
| 3040.713 | 0.84812 |  | 3040.851 | 0.76981 |
| 3042.141 | 0.84796 |  | 3042.279 | 0.77054 |
| 3043.57 | 0.84782 |  | 3043.707 | 0.77137 |
| 3044.998 | 0.84768 |  | 3045.135 | 0.77231 |
| 3046.426 | 0.84756 |  | 3046.564 | 0.77339 |
| 3047.854 | 0.84746 |  | 3047.992 | 0.77464 |
| 3049.283 | 0.84737 |  | 3049.42 | 0.77606 |
| 3050.711 | 0.84729 |  | 3050.849 | 0.77766 |
| 3052.139 | 0.8472 |  | 3052.277 | 0.77942 |
| 3053.567 | 0.84708 |  | 3053.705 | 0.78134 |
| 3054.996 | 0.84695 |  | 3055.133 | 0.78336 |
| 3056.424 | 0.84681 |  | 3056.562 | 0.78539 |
| 3057.852 | 0.84667 |  | 3057.99 | 0.78734 |
| 3059.28 | 0.84653 |  | 3059.418 | 0.78914 |
| 3060.708 | 0.84639 |  | 3060.847 | 0.79074 |
| 3062.137 | 0.84627 |  | 3062.275 | 0.79209 |
| 3063.565 | 0.84615 |  | 3063.703 | 0.79315 |
| 3064.993 | 0.84606 |  | 3065.132 | 0.79389 |
| 3066.421 | 0.84596 |  | 3066.56 | 0.79433 |
| 3067.85 | 0.84585 |  | 3067.988 | 0.79452 |
| 3069.278 | 0.84572 |  | 3069.416 | 0.7945 |
| 3070.706 | 0.84557 |  | 3070.845 | 0.79433 |
| 3072.134 | 0.84542 |  | 3072.273 | 0.79404 |
| 3073.563 | 0.84527 |  | 3073.701 | 0.7937 |
| 3074.991 | 0.84512 |  | 3075.13 | 0.79337 |
| 3076.419 | 0.84498 |  | 3076.558 | 0.79307 |
| 3077.847 | 0.84485 |  | 3077.986 | 0.79274 |
| 3079.276 | 0.84473 |  | 3079.415 | 0.79237 |
| 3080.704 | 0.84463 |  | 3080.843 | 0.79197 |
| 3082.132 | 0.84453 |  | 3082.271 | 0.79159 |
| 3083.56 | 0.84442 |  | 3083.699 | 0.79122 |
| 3084.988 | 0.84429 |  | 3085.128 | 0.79082 |
| 3086.417 | 0.84414 |  | 3086.556 | 0.79039 |
| 3087.845 | 0.84397 |  | 3087.984 | 0.78994 |
| 3089.273 | 0.84379 |  | 3089.413 | 0.78951 |
| 3090.701 | 0.84362 |  | 3090.841 | 0.78909 |
| 3092.13 | 0.84346 |  | 3092.269 | 0.78865 |
| 3093.558 | 0.84334 |  | 3093.698 | 0.78816 |
| 3094.986 | 0.84323 |  | 3095.126 | 0.78763 |
| 3096.414 | 0.84312 |  | 3096.554 | 0.78707 |
| 3097.843 | 0.843 |  | 3097.982 | 0.78651 |
| 3099.271 | 0.84288 |  | 3099.411 | 0.78596 |
| 3100.699 | 0.84276 |  | 3100.839 | 0.78541 |
| 3102.127 | 0.84265 |  | 3102.267 | 0.78486 |
| 3103.556 | 0.84254 |  | 3103.696 | 0.78433 |
| 3104.984 | 0.84242 |  | 3105.124 | 0.78383 |
| 3106.412 | 0.84229 |  | 3106.552 | 0.78334 |
| 3107.84 | 0.84215 |  | 3107.981 | 0.78285 |
| 3109.268 | 0.84201 |  | 3109.409 | 0.78236 |
| 3110.697 | 0.84187 |  | 3110.837 | 0.78188 |
| 3112.125 | 0.84175 |  | 3112.265 | 0.78146 |
| 3113.553 | 0.84165 |  | 3113.694 | 0.78112 |
| 3114.981 | 0.84155 |  | 3115.122 | 0.78085 |
| 3116.41 | 0.84143 |  | 3116.55 | 0.78067 |
| 3117.838 | 0.8413 |  | 3117.979 | 0.78057 |
| 3119.266 | 0.84115 |  | 3119.407 | 0.78055 |
| 3120.694 | 0.841 |  | 3120.835 | 0.78061 |
| 3122.123 | 0.84085 |  | 3122.264 | 0.78071 |
| 3123.551 | 0.84069 |  | 3123.692 | 0.78085 |
| 3124.979 | 0.84053 |  | 3125.12 | 0.78103 |
| 3126.407 | 0.84036 |  | 3126.548 | 0.78127 |
| 3127.836 | 0.84019 |  | 3127.977 | 0.78157 |
| 3129.264 | 0.84005 |  | 3129.405 | 0.78189 |
| 3130.692 | 0.83992 |  | 3130.833 | 0.7822 |
| 3132.12 | 0.83979 |  | 3132.262 | 0.78253 |
| 3133.548 | 0.83966 |  | 3133.69 | 0.78294 |
| 3134.977 | 0.83951 |  | 3135.118 | 0.78345 |
| 3136.405 | 0.83936 |  | 3136.547 | 0.78403 |
| 3137.833 | 0.83922 |  | 3137.975 | 0.78463 |
| 3139.261 | 0.83908 |  | 3139.403 | 0.78524 |
| 3140.69 | 0.83895 |  | 3140.831 | 0.78587 |
| 3142.118 | 0.83883 |  | 3142.26 | 0.78653 |
| 3143.546 | 0.8387 |  | 3143.688 | 0.78722 |
| 3144.974 | 0.83857 |  | 3145.116 | 0.78792 |
| 3146.403 | 0.83845 |  | 3146.545 | 0.78865 |
| 3147.831 | 0.83834 |  | 3147.973 | 0.78939 |
| 3149.259 | 0.83825 |  | 3149.401 | 0.79015 |
| 3150.687 | 0.83816 |  | 3150.83 | 0.79091 |
| 3152.116 | 0.83805 |  | 3152.258 | 0.7917 |
| 3153.544 | 0.83793 |  | 3153.686 | 0.79254 |
| 3154.972 | 0.83777 |  | 3155.114 | 0.79341 |
| 3156.4 | 0.8376 |  | 3156.543 | 0.79429 |
| 3157.829 | 0.83742 |  | 3157.971 | 0.7952 |
| 3159.257 | 0.83724 |  | 3159.399 | 0.79612 |
| 3160.685 | 0.83709 |  | 3160.828 | 0.79705 |
| 3162.113 | 0.83696 |  | 3162.256 | 0.79797 |
| 3163.541 | 0.83684 |  | 3163.684 | 0.79885 |
| 3164.97 | 0.83674 |  | 3165.113 | 0.79972 |
| 3166.398 | 0.83663 |  | 3166.541 | 0.80062 |
| 3167.826 | 0.83651 |  | 3167.969 | 0.80155 |
| 3169.254 | 0.8364 |  | 3169.397 | 0.80246 |
| 3170.683 | 0.83626 |  | 3170.826 | 0.80332 |
| 3172.111 | 0.83611 |  | 3172.254 | 0.80418 |
| 3173.539 | 0.83595 |  | 3173.682 | 0.80504 |
| 3174.967 | 0.8358 |  | 3175.111 | 0.80589 |
| 3176.396 | 0.83569 |  | 3176.539 | 0.8067 |
| 3177.824 | 0.8356 |  | 3177.967 | 0.80745 |
| 3179.252 | 0.83551 |  | 3179.396 | 0.80818 |
| 3180.68 | 0.8354 |  | 3180.824 | 0.80892 |
| 3182.109 | 0.83527 |  | 3182.252 | 0.80967 |
| 3183.537 | 0.83515 |  | 3183.68 | 0.81041 |
| 3184.965 | 0.83507 |  | 3185.109 | 0.81112 |
| 3186.393 | 0.83499 |  | 3186.537 | 0.81178 |
| 3187.821 | 0.8349 |  | 3187.965 | 0.8124 |
| 3189.25 | 0.83479 |  | 3189.394 | 0.81298 |
| 3190.678 | 0.83467 |  | 3190.822 | 0.81355 |
| 3192.106 | 0.83455 |  | 3192.25 | 0.81413 |
| 3193.534 | 0.83442 |  | 3193.679 | 0.81472 |
| 3194.963 | 0.83431 |  | 3195.107 | 0.8153 |
| 3196.391 | 0.83423 |  | 3196.535 | 0.81585 |
| 3197.819 | 0.83416 |  | 3197.963 | 0.81639 |
| 3199.247 | 0.83409 |  | 3199.392 | 0.81691 |
| 3200.676 | 0.834 |  | 3200.82 | 0.81742 |
| 3202.104 | 0.83389 |  | 3202.248 | 0.81791 |
| 3203.532 | 0.83377 |  | 3203.677 | 0.81838 |
| 3204.96 | 0.83365 |  | 3205.105 | 0.81886 |
| 3206.389 | 0.83354 |  | 3206.533 | 0.81936 |
| 3207.817 | 0.83343 |  | 3207.962 | 0.8199 |
| 3209.245 | 0.83334 |  | 3209.39 | 0.82045 |
| 3210.673 | 0.83326 |  | 3210.818 | 0.82098 |
| 3212.101 | 0.83319 |  | 3212.246 | 0.82149 |
| 3213.53 | 0.83312 |  | 3213.675 | 0.82199 |
| 3214.958 | 0.83306 |  | 3215.103 | 0.8225 |
| 3216.386 | 0.83298 |  | 3216.531 | 0.82302 |
| 3217.814 | 0.8329 |  | 3217.96 | 0.82359 |
| 3219.243 | 0.83282 |  | 3219.388 | 0.82421 |
| 3220.671 | 0.83275 |  | 3220.816 | 0.82483 |
| 3222.099 | 0.83266 |  | 3222.245 | 0.82539 |
| 3223.527 | 0.83258 |  | 3223.673 | 0.82591 |
| 3224.956 | 0.83251 |  | 3225.101 | 0.82645 |
| 3226.384 | 0.83247 |  | 3226.529 | 0.82704 |
| 3227.812 | 0.83246 |  | 3227.958 | 0.82765 |
| 3229.24 | 0.83245 |  | 3229.386 | 0.82827 |
| 3230.669 | 0.83241 |  | 3230.814 | 0.82887 |
| 3232.097 | 0.83233 |  | 3232.243 | 0.82945 |
| 3233.525 | 0.83222 |  | 3233.671 | 0.83004 |
| 3234.953 | 0.83211 |  | 3235.099 | 0.83065 |
| 3236.381 | 0.83201 |  | 3236.528 | 0.8313 |
| 3237.81 | 0.83193 |  | 3237.956 | 0.83197 |
| 3239.238 | 0.83186 |  | 3239.384 | 0.83265 |
| 3240.666 | 0.83177 |  | 3240.812 | 0.83329 |
| 3242.094 | 0.8317 |  | 3242.241 | 0.83388 |
| 3243.523 | 0.83167 |  | 3243.669 | 0.83443 |
| 3244.951 | 0.83167 |  | 3245.097 | 0.83498 |
| 3246.379 | 0.83165 |  | 3246.526 | 0.83554 |
| 3247.807 | 0.83158 |  | 3247.954 | 0.83616 |
| 3249.236 | 0.83144 |  | 3249.382 | 0.83681 |
| 3250.664 | 0.83128 |  | 3250.811 | 0.83748 |
| 3252.092 | 0.83117 |  | 3252.239 | 0.83813 |
| 3253.52 | 0.8311 |  | 3253.667 | 0.83874 |
| 3254.949 | 0.83108 |  | 3255.095 | 0.83933 |
| 3256.377 | 0.83107 |  | 3256.524 | 0.83995 |
| 3257.805 | 0.83104 |  | 3257.952 | 0.8406 |
| 3259.233 | 0.83098 |  | 3259.38 | 0.84127 |
| 3260.661 | 0.83091 |  | 3260.809 | 0.84192 |
| 3262.09 | 0.83081 |  | 3262.237 | 0.84252 |
| 3263.518 | 0.83072 |  | 3263.665 | 0.84306 |
| 3264.946 | 0.83065 |  | 3265.094 | 0.84354 |
| 3266.374 | 0.83059 |  | 3266.522 | 0.844 |
| 3267.803 | 0.83054 |  | 3267.95 | 0.84445 |
| 3269.231 | 0.83047 |  | 3269.378 | 0.84487 |
| 3270.659 | 0.83039 |  | 3270.807 | 0.84527 |
| 3272.087 | 0.83031 |  | 3272.235 | 0.84567 |
| 3273.516 | 0.83026 |  | 3273.663 | 0.84611 |
| 3274.944 | 0.83024 |  | 3275.092 | 0.84663 |
| 3276.372 | 0.83022 |  | 3276.52 | 0.84721 |
| 3277.8 | 0.8302 |  | 3277.948 | 0.84779 |
| 3279.229 | 0.83015 |  | 3279.377 | 0.8483 |
| 3280.657 | 0.83007 |  | 3280.805 | 0.84875 |
| 3282.085 | 0.82998 |  | 3282.233 | 0.84918 |
| 3283.513 | 0.82987 |  | 3283.661 | 0.8496 |
| 3284.941 | 0.82976 |  | 3285.09 | 0.84999 |
| 3286.37 | 0.82966 |  | 3286.518 | 0.85034 |
| 3287.798 | 0.8296 |  | 3287.946 | 0.85064 |
| 3289.226 | 0.82956 |  | 3289.375 | 0.85092 |
| 3290.654 | 0.82953 |  | 3290.803 | 0.85118 |
| 3292.083 | 0.82948 |  | 3292.231 | 0.85148 |
| 3293.511 | 0.82941 |  | 3293.66 | 0.85183 |
| 3294.939 | 0.82933 |  | 3295.088 | 0.85222 |
| 3296.367 | 0.82926 |  | 3296.516 | 0.85265 |
| 3297.796 | 0.82919 |  | 3297.944 | 0.8531 |
| 3299.224 | 0.8291 |  | 3299.373 | 0.85355 |
| 3300.652 | 0.829 |  | 3300.801 | 0.85395 |
| 3302.08 | 0.8289 |  | 3302.229 | 0.85429 |
| 3303.509 | 0.82881 |  | 3303.658 | 0.8546 |
| 3304.937 | 0.82877 |  | 3305.086 | 0.85489 |
| 3306.365 | 0.82876 |  | 3306.514 | 0.85521 |
| 3307.793 | 0.82876 |  | 3307.943 | 0.85558 |
| 3309.221 | 0.82876 |  | 3309.371 | 0.85599 |
| 3310.65 | 0.82872 |  | 3310.799 | 0.85643 |
| 3312.078 | 0.82863 |  | 3312.227 | 0.85688 |
| 3313.506 | 0.82851 |  | 3313.656 | 0.85733 |
| 3314.934 | 0.82838 |  | 3315.084 | 0.85777 |
| 3316.363 | 0.82825 |  | 3316.512 | 0.85817 |
| 3317.791 | 0.82814 |  | 3317.941 | 0.85855 |
| 3319.219 | 0.82805 |  | 3319.369 | 0.85891 |
| 3320.647 | 0.82796 |  | 3320.797 | 0.85926 |
| 3322.076 | 0.82787 |  | 3322.226 | 0.85958 |
| 3323.504 | 0.8278 |  | 3323.654 | 0.85988 |
| 3324.932 | 0.82773 |  | 3325.082 | 0.86018 |
| 3326.36 | 0.82767 |  | 3326.51 | 0.86051 |
| 3327.789 | 0.82759 |  | 3327.939 | 0.86083 |
| 3329.217 | 0.8275 |  | 3329.367 | 0.86116 |
| 3330.645 | 0.82739 |  | 3330.795 | 0.86149 |
| 3332.073 | 0.8273 |  | 3332.224 | 0.86183 |
| 3333.501 | 0.82723 |  | 3333.652 | 0.86213 |
| 3334.93 | 0.82719 |  | 3335.08 | 0.86235 |
| 3336.358 | 0.82718 |  | 3336.509 | 0.8625 |
| 3337.786 | 0.82714 |  | 3337.937 | 0.86263 |
| 3339.214 | 0.82707 |  | 3339.365 | 0.8628 |
| 3340.643 | 0.82696 |  | 3340.793 | 0.86301 |
| 3342.071 | 0.82686 |  | 3342.222 | 0.86324 |
| 3343.499 | 0.82677 |  | 3343.65 | 0.86349 |
| 3344.927 | 0.82672 |  | 3345.078 | 0.86372 |
| 3346.356 | 0.82668 |  | 3346.507 | 0.86389 |
| 3347.784 | 0.82665 |  | 3347.935 | 0.86398 |
| 3349.212 | 0.8266 |  | 3349.363 | 0.86401 |
| 3350.64 | 0.82651 |  | 3350.792 | 0.86407 |
| 3352.069 | 0.82641 |  | 3352.22 | 0.86421 |
| 3353.497 | 0.82632 |  | 3353.648 | 0.86441 |
| 3354.925 | 0.82626 |  | 3355.076 | 0.86459 |
| 3356.353 | 0.82623 |  | 3356.505 | 0.86472 |
| 3357.781 | 0.82617 |  | 3357.933 | 0.86483 |
| 3359.21 | 0.82608 |  | 3359.361 | 0.86496 |
| 3360.638 | 0.82598 |  | 3360.79 | 0.8651 |
| 3362.066 | 0.82592 |  | 3362.218 | 0.86522 |
| 3363.494 | 0.82591 |  | 3363.646 | 0.86529 |
| 3364.923 | 0.82595 |  | 3365.075 | 0.86528 |
| 3366.351 | 0.82598 |  | 3366.503 | 0.86524 |
| 3367.779 | 0.82597 |  | 3367.931 | 0.86522 |
| 3369.207 | 0.82588 |  | 3369.359 | 0.86529 |
| 3370.636 | 0.82574 |  | 3370.788 | 0.86544 |
| 3372.064 | 0.8256 |  | 3372.216 | 0.8656 |
| 3373.492 | 0.82548 |  | 3373.644 | 0.8657 |
| 3374.92 | 0.82542 |  | 3375.073 | 0.86576 |
| 3376.349 | 0.82539 |  | 3376.501 | 0.86581 |
| 3377.777 | 0.82535 |  | 3377.929 | 0.86586 |
| 3379.205 | 0.82529 |  | 3379.358 | 0.8659 |
| 3380.633 | 0.8252 |  | 3380.786 | 0.86595 |
| 3382.061 | 0.82511 |  | 3382.214 | 0.86604 |
| 3383.49 | 0.82505 |  | 3383.642 | 0.86613 |
| 3384.918 | 0.82501 |  | 3385.071 | 0.86619 |
| 3386.346 | 0.82498 |  | 3386.499 | 0.86619 |
| 3387.774 | 0.82495 |  | 3387.927 | 0.86617 |
| 3389.203 | 0.82494 |  | 3389.356 | 0.86614 |
| 3390.631 | 0.82497 |  | 3390.784 | 0.86612 |
| 3392.059 | 0.82502 |  | 3392.212 | 0.86616 |
| 3393.487 | 0.82507 |  | 3393.641 | 0.86627 |
| 3394.916 | 0.82508 |  | 3395.069 | 0.86636 |
| 3396.344 | 0.82506 |  | 3396.497 | 0.86638 |
| 3397.772 | 0.82505 |  | 3397.925 | 0.8664 |
| 3399.2 | 0.82506 |  | 3399.354 | 0.8665 |
| 3400.629 | 0.82506 |  | 3400.782 | 0.86669 |
| 3402.057 | 0.82504 |  | 3402.21 | 0.86686 |
| 3403.485 | 0.82499 |  | 3403.639 | 0.86694 |
| 3404.913 | 0.82492 |  | 3405.067 | 0.86695 |
| 3406.341 | 0.82487 |  | 3406.495 | 0.86691 |
| 3407.77 | 0.82485 |  | 3407.924 | 0.86688 |
| 3409.198 | 0.82485 |  | 3409.352 | 0.86688 |
| 3410.626 | 0.82484 |  | 3410.78 | 0.86693 |
| 3412.054 | 0.8248 |  | 3412.208 | 0.867 |
| 3413.483 | 0.8247 |  | 3413.637 | 0.86706 |
| 3414.911 | 0.82455 |  | 3415.065 | 0.8671 |
| 3416.339 | 0.82442 |  | 3416.493 | 0.86712 |
| 3417.767 | 0.82436 |  | 3417.922 | 0.86715 |
| 3419.196 | 0.8244 |  | 3419.35 | 0.86719 |
| 3420.624 | 0.82449 |  | 3420.778 | 0.86723 |
| 3422.052 | 0.82456 |  | 3422.207 | 0.86724 |
| 3423.48 | 0.82455 |  | 3423.635 | 0.86725 |
| 3424.909 | 0.8245 |  | 3425.063 | 0.86727 |
| 3426.337 | 0.82446 |  | 3426.491 | 0.86731 |
| 3427.765 | 0.82445 |  | 3427.92 | 0.86734 |
| 3429.193 | 0.82447 |  | 3429.348 | 0.86735 |
| 3430.621 | 0.82449 |  | 3430.776 | 0.86739 |
| 3432.05 | 0.82451 |  | 3432.205 | 0.86748 |
| 3433.478 | 0.82454 |  | 3433.633 | 0.86758 |
| 3434.906 | 0.82457 |  | 3435.061 | 0.86766 |
| 3436.334 | 0.82458 |  | 3436.49 | 0.8677 |
| 3437.763 | 0.82458 |  | 3437.918 | 0.86774 |
| 3439.191 | 0.82459 |  | 3439.346 | 0.8678 |
| 3440.619 | 0.82462 |  | 3440.774 | 0.86789 |
| 3442.047 | 0.8247 |  | 3442.203 | 0.86802 |
| 3443.476 | 0.82483 |  | 3443.631 | 0.86817 |
| 3444.904 | 0.82506 |  | 3445.059 | 0.86829 |
| 3446.332 | 0.82536 |  | 3446.488 | 0.86833 |
| 3447.76 | 0.82567 |  | 3447.916 | 0.86835 |
| 3449.189 | 0.8259 |  | 3449.344 | 0.86842 |
| 3450.617 | 0.82601 |  | 3450.773 | 0.86856 |
| 3452.045 | 0.82606 |  | 3452.201 | 0.86871 |
| 3453.473 | 0.82614 |  | 3453.629 | 0.86886 |
| 3454.901 | 0.82629 |  | 3455.057 | 0.86901 |
| 3456.33 | 0.8265 |  | 3456.486 | 0.86915 |
| 3457.758 | 0.82675 |  | 3457.914 | 0.86928 |
| 3459.186 | 0.82701 |  | 3459.342 | 0.86939 |
| 3460.614 | 0.82729 |  | 3460.771 | 0.8695 |
| 3462.043 | 0.82758 |  | 3462.199 | 0.86963 |
| 3463.471 | 0.82786 |  | 3463.627 | 0.86976 |
| 3464.899 | 0.82813 |  | 3465.056 | 0.86986 |
| 3466.327 | 0.82837 |  | 3466.484 | 0.86993 |
| 3467.756 | 0.82857 |  | 3467.912 | 0.87 |
| 3469.184 | 0.82871 |  | 3469.34 | 0.87012 |
| 3470.612 | 0.82881 |  | 3470.769 | 0.87028 |
| 3472.04 | 0.82894 |  | 3472.197 | 0.87045 |
| 3473.469 | 0.82912 |  | 3473.625 | 0.87057 |
| 3474.897 | 0.82936 |  | 3475.054 | 0.87066 |
| 3476.325 | 0.8296 |  | 3476.482 | 0.87075 |
| 3477.753 | 0.82984 |  | 3477.91 | 0.87086 |
| 3479.181 | 0.83009 |  | 3479.339 | 0.87101 |
| 3480.61 | 0.8304 |  | 3480.767 | 0.87119 |
| 3482.038 | 0.83074 |  | 3482.195 | 0.87139 |
| 3483.466 | 0.83105 |  | 3483.623 | 0.87156 |
| 3484.894 | 0.83132 |  | 3485.052 | 0.87169 |
| 3486.323 | 0.83159 |  | 3486.48 | 0.87178 |
| 3487.751 | 0.83188 |  | 3487.908 | 0.87188 |
| 3489.179 | 0.83221 |  | 3489.337 | 0.87202 |
| 3490.607 | 0.83252 |  | 3490.765 | 0.87217 |
| 3492.036 | 0.83281 |  | 3492.193 | 0.87232 |
| 3493.464 | 0.83315 |  | 3493.622 | 0.87244 |
| 3494.892 | 0.83354 |  | 3495.05 | 0.87254 |
| 3496.32 | 0.83392 |  | 3496.478 | 0.87268 |
| 3497.749 | 0.83421 |  | 3497.906 | 0.87288 |
| 3499.177 | 0.83445 |  | 3499.335 | 0.87312 |
| 3500.605 | 0.83477 |  | 3500.763 | 0.87329 |
| 3502.033 | 0.83522 |  | 3502.191 | 0.87333 |
| 3503.461 | 0.83572 |  | 3503.62 | 0.87335 |
| 3504.89 | 0.8361 |  | 3505.048 | 0.87344 |
| 3506.318 | 0.83634 |  | 3506.476 | 0.87361 |
| 3507.746 | 0.83656 |  | 3507.905 | 0.87377 |
| 3509.174 | 0.83688 |  | 3509.333 | 0.87392 |
| 3510.603 | 0.83727 |  | 3510.761 | 0.8741 |
| 3512.031 | 0.8376 |  | 3512.189 | 0.8743 |
| 3513.459 | 0.83782 |  | 3513.618 | 0.87451 |
| 3514.887 | 0.83797 |  | 3515.046 | 0.87467 |
| 3516.316 | 0.83813 |  | 3516.474 | 0.87478 |
| 3517.744 | 0.83833 |  | 3517.903 | 0.87484 |
| 3519.172 | 0.83856 |  | 3519.331 | 0.87488 |
| 3520.6 | 0.83886 |  | 3520.759 | 0.87491 |
| 3522.029 | 0.83926 |  | 3522.188 | 0.87495 |
| 3523.457 | 0.83972 |  | 3523.616 | 0.87502 |
| 3524.885 | 0.84021 |  | 3525.044 | 0.87512 |
| 3526.313 | 0.84068 |  | 3526.472 | 0.87524 |
| 3527.741 | 0.84111 |  | 3527.901 | 0.87533 |
| 3529.17 | 0.84144 |  | 3529.329 | 0.87545 |
| 3530.598 | 0.84165 |  | 3530.757 | 0.87563 |
| 3532.026 | 0.84174 |  | 3532.186 | 0.87584 |
| 3533.454 | 0.84184 |  | 3533.614 | 0.87602 |
| 3534.883 | 0.84205 |  | 3535.042 | 0.8761 |
| 3536.311 | 0.84237 |  | 3536.471 | 0.87612 |
| 3537.739 | 0.84269 |  | 3537.899 | 0.87617 |
| 3539.167 | 0.8429 |  | 3539.327 | 0.87629 |
| 3540.596 | 0.84303 |  | 3540.755 | 0.87642 |
| 3542.024 | 0.84321 |  | 3542.184 | 0.87649 |
| 3543.452 | 0.8436 |  | 3543.612 | 0.8765 |
| 3544.88 | 0.84422 |  | 3545.04 | 0.87649 |
| 3546.309 | 0.84486 |  | 3546.469 | 0.87653 |
| 3547.737 | 0.84529 |  | 3547.897 | 0.87661 |
| 3549.165 | 0.84549 |  | 3549.325 | 0.8767 |
| 3550.593 | 0.84563 |  | 3550.754 | 0.87675 |
| 3552.021 | 0.84584 |  | 3552.182 | 0.87681 |
| 3553.45 | 0.8461 |  | 3553.61 | 0.87693 |
| 3554.878 | 0.84629 |  | 3555.038 | 0.87709 |
| 3556.306 | 0.84637 |  | 3556.467 | 0.87724 |
| 3557.734 | 0.84643 |  | 3557.895 | 0.87733 |
| 3559.163 | 0.84654 |  | 3559.323 | 0.87738 |
| 3560.591 | 0.84667 |  | 3560.752 | 0.87742 |
| 3562.019 | 0.84678 |  | 3562.18 | 0.87748 |
| 3563.447 | 0.84696 |  | 3563.608 | 0.8775 |
| 3564.876 | 0.84747 |  | 3565.037 | 0.8774 |
| 3566.304 | 0.84844 |  | 3566.465 | 0.8772 |
| 3567.732 | 0.8495 |  | 3567.893 | 0.87714 |
| 3569.16 | 0.85004 |  | 3569.321 | 0.87734 |
| 3570.589 | 0.84998 |  | 3570.75 | 0.8776 |
| 3572.017 | 0.84966 |  | 3572.178 | 0.87778 |
| 3573.445 | 0.84943 |  | 3573.606 | 0.87787 |
| 3574.873 | 0.84941 |  | 3575.035 | 0.87792 |
| 3576.301 | 0.8496 |  | 3576.463 | 0.878 |
| 3577.73 | 0.84989 |  | 3577.891 | 0.87809 |
| 3579.158 | 0.85018 |  | 3579.32 | 0.87816 |
| 3580.586 | 0.85035 |  | 3580.748 | 0.87819 |
| 3582.014 | 0.85036 |  | 3582.176 | 0.87819 |
| 3583.443 | 0.85032 |  | 3583.604 | 0.87818 |
| 3584.871 | 0.85049 |  | 3585.033 | 0.87808 |
| 3586.299 | 0.85108 |  | 3586.461 | 0.87787 |
| 3587.727 | 0.85192 |  | 3587.889 | 0.8777 |
| 3589.156 | 0.8525 |  | 3589.318 | 0.87779 |
| 3590.584 | 0.85258 |  | 3590.746 | 0.87798 |
| 3592.012 | 0.85244 |  | 3592.174 | 0.87805 |
| 3593.44 | 0.85245 |  | 3593.603 | 0.87794 |
| 3594.869 | 0.85271 |  | 3595.031 | 0.87777 |
| 3596.297 | 0.85308 |  | 3596.459 | 0.87768 |
| 3597.725 | 0.85339 |  | 3597.887 | 0.87768 |
| 3599.153 | 0.85361 |  | 3599.316 | 0.87771 |
| 3600.581 | 0.85377 |  | 3600.744 | 0.87774 |
| 3602.01 | 0.85379 |  | 3602.172 | 0.87782 |
| 3603.438 | 0.85366 |  | 3603.601 | 0.87794 |
| 3604.866 | 0.85352 |  | 3605.029 | 0.87802 |
| 3606.294 | 0.8536 |  | 3606.457 | 0.87797 |
| 3607.723 | 0.854 |  | 3607.886 | 0.87785 |
| 3609.151 | 0.8546 |  | 3609.314 | 0.8778 |
| 3610.579 | 0.85516 |  | 3610.742 | 0.87783 |
| 3612.007 | 0.85554 |  | 3612.17 | 0.87782 |
| 3613.436 | 0.85575 |  | 3613.599 | 0.87775 |
| 3614.864 | 0.85586 |  | 3615.027 | 0.87772 |
| 3616.292 | 0.85603 |  | 3616.455 | 0.87774 |
| 3617.72 | 0.85642 |  | 3617.884 | 0.87766 |
| 3619.149 | 0.85697 |  | 3619.312 | 0.87747 |
| 3620.577 | 0.8573 |  | 3620.74 | 0.87735 |
| 3622.005 | 0.85707 |  | 3622.169 | 0.87735 |
| 3623.433 | 0.8564 |  | 3623.597 | 0.87738 |
| 3624.861 | 0.85567 |  | 3625.025 | 0.87736 |
| 3626.29 | 0.85542 |  | 3626.453 | 0.87721 |
| 3627.718 | 0.8561 |  | 3627.882 | 0.87689 |
| 3629.146 | 0.85748 |  | 3629.31 | 0.87657 |
| 3630.574 | 0.85855 |  | 3630.738 | 0.87657 |
| 3632.003 | 0.85876 |  | 3632.167 | 0.87672 |
| 3633.431 | 0.85843 |  | 3633.595 | 0.87682 |
| 3634.859 | 0.85801 |  | 3635.023 | 0.87682 |
| 3636.287 | 0.85771 |  | 3636.452 | 0.87676 |
| 3637.716 | 0.85754 |  | 3637.88 | 0.8767 |
| 3639.144 | 0.85743 |  | 3639.308 | 0.87667 |
| 3640.572 | 0.8573 |  | 3640.736 | 0.87666 |
| 3642 | 0.85708 |  | 3642.165 | 0.87664 |
| 3643.429 | 0.85676 |  | 3643.593 | 0.87663 |
| 3644.857 | 0.85649 |  | 3645.021 | 0.87661 |
| 3646.285 | 0.85667 |  | 3646.45 | 0.87647 |
| 3647.713 | 0.85775 |  | 3647.878 | 0.87601 |
| 3649.141 | 0.8595 |  | 3649.306 | 0.87535 |
| 3650.57 | 0.86064 |  | 3650.735 | 0.8751 |
| 3651.998 | 0.86057 |  | 3652.163 | 0.87527 |
| 3653.426 | 0.85994 |  | 3653.591 | 0.87543 |
| 3654.854 | 0.85941 |  | 3655.019 | 0.87537 |
| 3656.283 | 0.85922 |  | 3656.448 | 0.87511 |
| 3657.711 | 0.85919 |  | 3657.876 | 0.87492 |
| 3659.139 | 0.85904 |  | 3659.304 | 0.87488 |
| 3660.567 | 0.8587 |  | 3660.733 | 0.87489 |
| 3661.996 | 0.85824 |  | 3662.161 | 0.8749 |
| 3663.424 | 0.85774 |  | 3663.589 | 0.87493 |
| 3664.852 | 0.85725 |  | 3665.018 | 0.87498 |
| 3666.28 | 0.85691 |  | 3666.446 | 0.87495 |
| 3667.709 | 0.85696 |  | 3667.874 | 0.87471 |
| 3669.137 | 0.85747 |  | 3669.302 | 0.87423 |
| 3670.565 | 0.85808 |  | 3670.731 | 0.87384 |
| 3671.993 | 0.85839 |  | 3672.159 | 0.87375 |
| 3673.421 | 0.85854 |  | 3673.587 | 0.87362 |
| 3674.85 | 0.85899 |  | 3675.016 | 0.87323 |
| 3676.278 | 0.85969 |  | 3676.444 | 0.873 |
| 3677.706 | 0.86 |  | 3677.872 | 0.87321 |
| 3679.134 | 0.85961 |  | 3679.301 | 0.87353 |
| 3680.563 | 0.85879 |  | 3680.729 | 0.87375 |
| 3681.991 | 0.85785 |  | 3682.157 | 0.87386 |
| 3683.419 | 0.85694 |  | 3683.585 | 0.87392 |
| 3684.847 | 0.85618 |  | 3685.014 | 0.87393 |
| 3686.276 | 0.8558 |  | 3686.442 | 0.87382 |
| 3687.704 | 0.85609 |  | 3687.87 | 0.8735 |
| 3689.132 | 0.85705 |  | 3689.299 | 0.87303 |
| 3690.56 | 0.85797 |  | 3690.727 | 0.87276 |
| 3691.989 | 0.85817 |  | 3692.155 | 0.87283 |
| 3693.417 | 0.85771 |  | 3693.584 | 0.87302 |
| 3694.845 | 0.85702 |  | 3695.012 | 0.87319 |
| 3696.273 | 0.85639 |  | 3696.44 | 0.87333 |
| 3697.701 | 0.85596 |  | 3697.868 | 0.87343 |
| 3699.13 | 0.85582 |  | 3699.297 | 0.87344 |
| 3700.558 | 0.85602 |  | 3700.725 | 0.87328 |
| 3701.986 | 0.85642 |  | 3702.153 | 0.87307 |
| 3703.414 | 0.85671 |  | 3703.582 | 0.873 |
| 3704.843 | 0.85665 |  | 3705.01 | 0.87309 |
| 3706.271 | 0.85632 |  | 3706.438 | 0.87319 |
| 3707.699 | 0.85608 |  | 3707.867 | 0.8732 |
| 3709.127 | 0.85639 |  | 3709.295 | 0.87306 |
| 3710.556 | 0.85744 |  | 3710.723 | 0.87286 |
| 3711.984 | 0.85865 |  | 3712.151 | 0.87286 |
| 3713.412 | 0.85914 |  | 3713.58 | 0.87311 |
| 3714.84 | 0.85879 |  | 3715.008 | 0.87336 |
| 3716.269 | 0.85804 |  | 3716.436 | 0.87343 |
| 3717.697 | 0.85733 |  | 3717.865 | 0.87334 |
| 3719.125 | 0.85691 |  | 3719.293 | 0.87315 |
| 3720.553 | 0.85683 |  | 3720.721 | 0.87292 |
| 3721.981 | 0.85701 |  | 3722.15 | 0.87268 |
| 3723.41 | 0.8573 |  | 3723.578 | 0.87243 |
| 3724.838 | 0.85752 |  | 3725.006 | 0.87215 |
| 3726.266 | 0.8575 |  | 3726.434 | 0.87195 |
| 3727.694 | 0.85716 |  | 3727.863 | 0.87189 |
| 3729.123 | 0.85659 |  | 3729.291 | 0.87194 |
| 3730.551 | 0.85606 |  | 3730.719 | 0.87199 |
| 3731.979 | 0.85594 |  | 3732.148 | 0.87194 |
| 3733.407 | 0.85654 |  | 3733.576 | 0.87171 |
| 3734.836 | 0.85775 |  | 3735.004 | 0.87133 |
| 3736.264 | 0.85871 |  | 3736.433 | 0.87107 |
| 3737.692 | 0.85876 |  | 3737.861 | 0.87115 |
| 3739.12 | 0.85802 |  | 3739.289 | 0.87145 |
| 3740.549 | 0.85703 |  | 3740.717 | 0.87174 |
| 3741.977 | 0.8563 |  | 3742.146 | 0.87182 |
| 3743.405 | 0.85618 |  | 3743.574 | 0.87154 |
| 3744.833 | 0.85654 |  | 3745.002 | 0.87109 |
| 3746.261 | 0.85683 |  | 3746.431 | 0.87092 |
| 3747.69 | 0.85687 |  | 3747.859 | 0.87095 |
| 3749.118 | 0.85704 |  | 3749.287 | 0.87085 |
| 3750.546 | 0.85763 |  | 3750.716 | 0.87058 |
| 3751.974 | 0.85816 |  | 3752.144 | 0.87049 |
| 3753.403 | 0.85802 |  | 3753.572 | 0.87069 |
| 3754.831 | 0.85732 |  | 3755 | 0.8709 |
| 3756.259 | 0.85659 |  | 3756.429 | 0.87099 |
| 3757.687 | 0.85619 |  | 3757.857 | 0.8709 |
| 3759.116 | 0.85608 |  | 3759.285 | 0.87075 |
| 3760.544 | 0.85596 |  | 3760.714 | 0.87075 |
| 3761.972 | 0.85563 |  | 3762.142 | 0.87087 |
| 3763.4 | 0.85515 |  | 3763.57 | 0.87095 |
| 3764.829 | 0.85475 |  | 3764.999 | 0.87089 |
| 3766.257 | 0.85459 |  | 3766.427 | 0.87076 |
| 3767.685 | 0.85471 |  | 3767.855 | 0.87061 |
| 3769.113 | 0.855 |  | 3769.283 | 0.87045 |
| 3770.541 | 0.85522 |  | 3770.712 | 0.87035 |
| 3771.97 | 0.85514 |  | 3772.14 | 0.87037 |
| 3773.398 | 0.85473 |  | 3773.568 | 0.87044 |
| 3774.826 | 0.8542 |  | 3774.997 | 0.87045 |
| 3776.254 | 0.85383 |  | 3776.425 | 0.87035 |
| 3777.683 | 0.85379 |  | 3777.853 | 0.87017 |
| 3779.111 | 0.85402 |  | 3779.282 | 0.86998 |
| 3780.539 | 0.85421 |  | 3780.71 | 0.86993 |
| 3781.967 | 0.85419 |  | 3782.138 | 0.86994 |
| 3783.396 | 0.85404 |  | 3783.566 | 0.86986 |
| 3784.824 | 0.8539 |  | 3784.995 | 0.86968 |
| 3786.252 | 0.8538 |  | 3786.423 | 0.86954 |
| 3787.68 | 0.85364 |  | 3787.851 | 0.86952 |
| 3789.109 | 0.85337 |  | 3789.28 | 0.86954 |
| 3790.537 | 0.85301 |  | 3790.708 | 0.86957 |
| 3791.965 | 0.85265 |  | 3792.136 | 0.8696 |
| 3793.393 | 0.85238 |  | 3793.565 | 0.86957 |
| 3794.821 | 0.85225 |  | 3794.993 | 0.86939 |
| 3796.25 | 0.85224 |  | 3796.421 | 0.86913 |
| 3797.678 | 0.85233 |  | 3797.849 | 0.86893 |
| 3799.106 | 0.85259 |  | 3799.278 | 0.86874 |
| 3800.534 | 0.85307 |  | 3800.706 | 0.86836 |
| 3801.963 | 0.85356 |  | 3802.134 | 0.86805 |
| 3803.391 | 0.85374 |  | 3803.563 | 0.86811 |
| 3804.819 | 0.85363 |  | 3804.991 | 0.86826 |
| 3806.247 | 0.85347 |  | 3806.419 | 0.86821 |
| 3807.676 | 0.8533 |  | 3807.848 | 0.86809 |
| 3809.104 | 0.85294 |  | 3809.276 | 0.86819 |
| 3810.532 | 0.85229 |  | 3810.704 | 0.86842 |
| 3811.96 | 0.85151 |  | 3812.132 | 0.86857 |
| 3813.389 | 0.85096 |  | 3813.561 | 0.8685 |
| 3814.817 | 0.85084 |  | 3814.989 | 0.86816 |
| 3816.245 | 0.8511 |  | 3816.417 | 0.86776 |
| 3817.673 | 0.8515 |  | 3817.846 | 0.86754 |
| 3819.101 | 0.85197 |  | 3819.274 | 0.86737 |
| 3820.53 | 0.85256 |  | 3820.702 | 0.8671 |
| 3821.958 | 0.85305 |  | 3822.131 | 0.86704 |
| 3823.386 | 0.85312 |  | 3823.559 | 0.86725 |
| 3824.814 | 0.85275 |  | 3824.987 | 0.86743 |
| 3826.243 | 0.85217 |  | 3826.415 | 0.86747 |
| 3827.671 | 0.85162 |  | 3827.844 | 0.86748 |
| 3829.099 | 0.85118 |  | 3829.272 | 0.86751 |
| 3830.527 | 0.85084 |  | 3830.7 | 0.8675 |
| 3831.956 | 0.85058 |  | 3832.129 | 0.86741 |
| 3833.384 | 0.85041 |  | 3833.557 | 0.8673 |
| 3834.812 | 0.85041 |  | 3834.985 | 0.86716 |
| 3836.24 | 0.85068 |  | 3836.414 | 0.86689 |
| 3837.669 | 0.85126 |  | 3837.842 | 0.86647 |
| 3839.097 | 0.85195 |  | 3839.27 | 0.86619 |
| 3840.525 | 0.85236 |  | 3840.698 | 0.86621 |
| 3841.953 | 0.85232 |  | 3842.127 | 0.86635 |
| 3843.381 | 0.85194 |  | 3843.555 | 0.86644 |
| 3844.81 | 0.85132 |  | 3844.983 | 0.86653 |
| 3846.238 | 0.8505 |  | 3846.412 | 0.86667 |
| 3847.666 | 0.84951 |  | 3847.84 | 0.86684 |
| 3849.094 | 0.84857 |  | 3849.268 | 0.86699 |
| 3850.523 | 0.8481 |  | 3850.697 | 0.86697 |
| 3851.951 | 0.84874 |  | 3852.125 | 0.86655 |
| 3853.379 | 0.85053 |  | 3853.553 | 0.86579 |
| 3854.807 | 0.85205 |  | 3854.981 | 0.86546 |
| 3856.236 | 0.85215 |  | 3856.41 | 0.86567 |
| 3857.664 | 0.85126 |  | 3857.838 | 0.866 |
| 3859.092 | 0.85013 |  | 3859.266 | 0.86626 |
| 3860.52 | 0.8493 |  | 3860.695 | 0.86639 |
| 3861.949 | 0.84901 |  | 3862.123 | 0.8663 |
| 3863.377 | 0.84915 |  | 3863.551 | 0.86604 |
| 3864.805 | 0.84934 |  | 3864.98 | 0.86587 |
| 3866.233 | 0.84931 |  | 3866.408 | 0.8659 |
| 3867.661 | 0.84917 |  | 3867.836 | 0.86589 |
| 3869.09 | 0.84921 |  | 3869.264 | 0.86567 |
| 3870.518 | 0.84953 |  | 3870.693 | 0.86541 |
| 3871.946 | 0.84989 |  | 3872.121 | 0.86534 |
| 3873.374 | 0.84994 |  | 3873.549 | 0.86538 |
| 3874.803 | 0.84956 |  | 3874.978 | 0.86544 |
| 3876.231 | 0.84894 |  | 3876.406 | 0.86556 |
| 3877.659 | 0.84838 |  | 3877.834 | 0.86564 |
| 3879.087 | 0.84811 |  | 3879.263 | 0.86555 |
| 3880.516 | 0.84819 |  | 3880.691 | 0.86535 |
| 3881.944 | 0.84844 |  | 3882.119 | 0.86526 |
| 3883.372 | 0.84868 |  | 3883.547 | 0.86521 |
| 3884.8 | 0.84878 |  | 3884.976 | 0.86505 |
| 3886.229 | 0.84867 |  | 3886.404 | 0.86501 |
| 3887.657 | 0.84841 |  | 3887.832 | 0.86519 |
| 3889.085 | 0.84816 |  | 3889.261 | 0.86536 |
| 3890.513 | 0.84813 |  | 3890.689 | 0.86524 |
| 3891.942 | 0.84829 |  | 3892.117 | 0.86498 |
| 3893.37 | 0.84828 |  | 3893.546 | 0.86491 |
| 3894.798 | 0.84788 |  | 3894.974 | 0.86502 |
| 3896.226 | 0.84724 |  | 3896.402 | 0.86507 |
| 3897.654 | 0.84672 |  | 3897.83 | 0.86495 |
| 3899.083 | 0.84664 |  | 3899.259 | 0.86474 |
| 3900.511 | 0.84707 |  | 3900.687 | 0.86458 |
| 3901.939 | 0.84781 |  | 3902.115 | 0.86446 |
| 3903.367 | 0.84853 |  | 3903.544 | 0.86428 |
| 3904.796 | 0.84888 |  | 3904.972 | 0.86413 |
| 3906.224 | 0.84871 |  | 3906.4 | 0.86418 |
| 3907.652 | 0.84815 |  | 3907.829 | 0.86439 |
| 3909.08 | 0.84747 |  | 3909.257 | 0.86459 |
| 3910.509 | 0.84685 |  | 3910.685 | 0.86469 |
| 3911.937 | 0.84637 |  | 3912.113 | 0.86474 |
| 3913.365 | 0.84607 |  | 3913.542 | 0.86477 |
| 3914.793 | 0.84598 |  | 3914.97 | 0.86472 |
| 3916.222 | 0.84612 |  | 3916.398 | 0.86457 |
| 3917.65 | 0.84637 |  | 3917.827 | 0.86437 |
| 3919.078 | 0.84651 |  | 3919.255 | 0.86423 |
| 3920.506 | 0.84646 |  | 3920.683 | 0.86418 |
| 3921.934 | 0.84633 |  | 3922.112 | 0.86415 |
| 3923.363 | 0.84628 |  | 3923.54 | 0.86405 |
| 3924.791 | 0.84629 |  | 3924.968 | 0.86396 |
| 3926.219 | 0.84623 |  | 3926.396 | 0.864 |
| 3927.647 | 0.84606 |  | 3927.825 | 0.86413 |
| 3929.076 | 0.84593 |  | 3929.253 | 0.86419 |
| 3930.504 | 0.84598 |  | 3930.681 | 0.86408 |
| 3931.932 | 0.8462 |  | 3932.11 | 0.86388 |
| 3933.36 | 0.84635 |  | 3933.538 | 0.86379 |
| 3934.789 | 0.84624 |  | 3934.966 | 0.86383 |
| 3936.217 | 0.84589 |  | 3936.395 | 0.86393 |
| 3937.645 | 0.84547 |  | 3937.823 | 0.86398 |
| 3939.073 | 0.84515 |  | 3939.251 | 0.86394 |
| 3940.502 | 0.84503 |  | 3940.679 | 0.86377 |
| 3941.93 | 0.8451 |  | 3942.108 | 0.86355 |
| 3943.358 | 0.84524 |  | 3943.536 | 0.86345 |
| 3944.786 | 0.84528 |  | 3944.964 | 0.8635 |
| 3946.214 | 0.84523 |  | 3946.393 | 0.86353 |
| 3947.643 | 0.84518 |  | 3947.821 | 0.86345 |
| 3949.071 | 0.84521 |  | 3949.249 | 0.86331 |
| 3950.499 | 0.84525 |  | 3950.678 | 0.86326 |
| 3951.927 | 0.84519 |  | 3952.106 | 0.86329 |
| 3953.356 | 0.84501 |  | 3953.534 | 0.86333 |
| 3954.784 | 0.84479 |  | 3954.962 | 0.86331 |
| 3956.212 | 0.84459 |  | 3956.391 | 0.86324 |
| 3957.64 | 0.84444 |  | 3957.819 | 0.86318 |
| 3959.069 | 0.84433 |  | 3959.247 | 0.86316 |
| 3960.497 | 0.84428 |  | 3960.676 | 0.86314 |
| 3961.925 | 0.84427 |  | 3962.104 | 0.86308 |
| 3963.353 | 0.84427 |  | 3963.532 | 0.86301 |
| 3964.782 | 0.84423 |  | 3964.961 | 0.86296 |
| 3966.21 | 0.84415 |  | 3966.389 | 0.86294 |
| 3967.638 | 0.84406 |  | 3967.817 | 0.8629 |
| 3969.066 | 0.84397 |  | 3969.245 | 0.86286 |
| 3970.494 | 0.84391 |  | 3970.674 | 0.86282 |
| 3971.923 | 0.84388 |  | 3972.102 | 0.86281 |
| 3973.351 | 0.84387 |  | 3973.53 | 0.86281 |
| 3974.779 | 0.84387 |  | 3974.959 | 0.86279 |
| 3976.207 | 0.84384 |  | 3976.387 | 0.86273 |
| 3977.636 | 0.84377 |  | 3977.815 | 0.86263 |
| 3979.064 | 0.8437 |  | 3979.244 | 0.86254 |
| 3980.492 | 0.84362 |  | 3980.672 | 0.86247 |
| 3981.92 | 0.84356 |  | 3982.1 | 0.86246 |
| 3983.349 | 0.8435 |  | 3983.528 | 0.86248 |
| 3984.777 | 0.84341 |  | 3984.957 | 0.86252 |
| 3986.205 | 0.84331 |  | 3986.385 | 0.86255 |
| 3987.633 | 0.84323 |  | 3987.813 | 0.86252 |
| 3989.062 | 0.8432 |  | 3989.242 | 0.86245 |
| 3990.49 | 0.8432 |  | 3990.67 | 0.86236 |
| 3991.918 | 0.84319 |  | 3992.098 | 0.8623 |
| 3993.346 | 0.84316 |  | 3993.527 | 0.86225 |
| 3994.774 | 0.84309 |  | 3994.955 | 0.8622 |
| 3996.203 | 0.843 |  | 3996.383 | 0.86214 |
| 3997.631 | 0.84288 |  | 3997.811 | 0.8621 |

**Table S2**. Source data for Fig. 1b.

| Fe_3_O_4_ nanoparticles | | Fe_3_O_4_/*Malva sylvestris* nanoparticles | |
| --- | --- | --- | --- |
| Angle | Det1Disc1 | Angle | Det1Disc1 |
| 5 | 95 |  |  |
| 5.05 | 78 | 10 | 57 |
| 5.1 | 94 | 10.05 | 38 |
| 5.15 | 90 | 10.1 | 57 |
| 5.2 | 92 | 10.15 | 52 |
| 5.25 | 78 | 10.2 | 42 |
| 5.3 | 76 | 10.25 | 52 |
| 5.35 | 87 | 10.3 | 47 |
| 5.4 | 81 | 10.35 | 48 |
| 5.45 | 80 | 10.4 | 47 |
| 5.5 | 80 | 10.45 | 49 |
| 5.55 | 75 | 10.5 | 44 |
| 5.6 | 78 | 10.55 | 49 |
| 5.65 | 57 | 10.6 | 47 |
| 5.7 | 71 | 10.65 | 52 |
| 5.75 | 70 | 10.7 | 55 |
| 5.8 | 94 | 10.75 | 44 |
| 5.85 | 76 | 10.8 | 47 |
| 5.9 | 86 | 10.85 | 35 |
| 5.95 | 93 | 10.9 | 49 |
| 6 | 76 | 10.95 | 41 |
| 6.05 | 83 | 11 | 43 |
| 6.1 | 66 | 11.05 | 36 |
| 6.15 | 92 | 11.1 | 40 |
| 6.2 | 75 | 11.15 | 50 |
| 6.25 | 88 | 11.2 | 44 |
| 6.3 | 82 | 11.25 | 36 |
| 6.35 | 80 | 11.3 | 39 |
| 6.4 | 78 | 11.35 | 37 |
| 6.45 | 82 | 11.4 | 41 |
| 6.5 | 75 | 11.45 | 38 |
| 6.55 | 79 | 11.5 | 44 |
| 6.6 | 91 | 11.55 | 47 |
| 6.65 | 64 | 11.6 | 43 |
| 6.7 | 87 | 11.65 | 36 |
| 6.75 | 83 | 11.7 | 33 |
| 6.8 | 81 | 11.75 | 37 |
| 6.85 | 66 | 11.8 | 42 |
| 6.9 | 76 | 11.85 | 38 |
| 6.95 | 77 | 11.9 | 32 |
| 7 | 87 | 11.95 | 41 |
| 7.05 | 82 | 12 | 46 |
| 7.1 | 72 | 12.05 | 53 |
| 7.15 | 70 | 12.1 | 35 |
| 7.2 | 69 | 12.15 | 41 |
| 7.25 | 85 | 12.2 | 27 |
| 7.3 | 78 | 12.25 | 32 |
| 7.35 | 93 | 12.3 | 34 |
| 7.4 | 73 | 12.35 | 36 |
| 7.45 | 82 | 12.4 | 39 |
| 7.5 | 84 | 12.45 | 32 |
| 7.55 | 89 | 12.5 | 35 |
| 7.6 | 74 | 12.55 | 39 |
| 7.65 | 92 | 12.6 | 42 |
| 7.7 | 90 | 12.65 | 30 |
| 7.75 | 78 | 12.7 | 29 |
| 7.8 | 91 | 12.75 | 31 |
| 7.85 | 82 | 12.8 | 31 |
| 7.9 | 79 | 12.85 | 38 |
| 7.95 | 78 | 12.9 | 31 |
| 8 | 75 | 12.95 | 32 |
| 8.05 | 94 | 13 | 20 |
| 8.1 | 69 | 13.05 | 29 |
| 8.15 | 93 | 13.1 | 25 |
| 8.2 | 88 | 13.15 | 33 |
| 8.25 | 83 | 13.2 | 18 |
| 8.3 | 88 | 13.25 | 26 |
| 8.35 | 82 | 13.3 | 31 |
| 8.4 | 92 | 13.35 | 25 |
| 8.45 | 93 | 13.4 | 20 |
| 8.5 | 105 | 13.45 | 16 |
| 8.55 | 106 | 13.5 | 18 |
| 8.6 | 85 | 13.55 | 39 |
| 8.65 | 67 | 13.6 | 25 |
| 8.7 | 88 | 13.65 | 17 |
| 8.75 | 93 | 13.7 | 33 |
| 8.8 | 87 | 13.75 | 13 |
| 8.85 | 106 | 13.8 | 15 |
| 8.9 | 103 | 13.85 | 15 |
| 8.95 | 107 | 13.9 | 15 |
| 9 | 107 | 13.95 | 17 |
| 9.05 | 87 | 14 | 23 |
| 9.1 | 98 | 14.05 | 23 |
| 9.15 | 79 | 14.1 | 20 |
| 9.2 | 102 | 14.15 | 16 |
| 9.25 | 82 | 14.2 | 21 |
| 9.3 | 100 | 14.25 | 18 |
| 9.35 | 87 | 14.3 | 16 |
| 9.4 | 105 | 14.35 | 13 |
| 9.45 | 89 | 14.4 | 20 |
| 9.5 | 92 | 14.45 | 18 |
| 9.55 | 84 | 14.5 | 16 |
| 9.6 | 97 | 14.55 | 13 |
| 9.65 | 85 | 14.6 | 23 |
| 9.7 | 86 | 14.65 | 18 |
| 9.75 | 81 | 14.7 | 14 |
| 9.8 | 76 | 14.75 | 6 |
| 9.85 | 104 | 14.8 | 21 |
| 9.9 | 105 | 14.85 | 20 |
| 9.95 | 101 | 14.9 | 10 |
| 10 | 85 | 14.95 | 17 |
| 10.05 | 88 | 15 | 13 |
| 10.1 | 86 | 15.05 | 11 |
| 10.15 | 98 | 15.1 | 17 |
| 10.2 | 98 | 15.15 | 12 |
| 10.25 | 99 | 15.2 | 18 |
| 10.3 | 99 | 15.25 | 15 |
| 10.35 | 101 | 15.3 | 16 |
| 10.4 | 104 | 15.35 | 14 |
| 10.45 | 107 | 15.4 | 15 |
| 10.5 | 82 | 15.45 | 16 |
| 10.55 | 87 | 15.5 | 8 |
| 10.6 | 102 | 15.55 | 14 |
| 10.65 | 83 | 15.6 | 13 |
| 10.7 | 119 | 15.65 | 17 |
| 10.75 | 95 | 15.7 | 12 |
| 10.8 | 102 | 15.75 | 10 |
| 10.85 | 98 | 15.8 | 12 |
| 10.9 | 97 | 15.85 | 11 |
| 10.95 | 97 | 15.9 | 8 |
| 11 | 102 | 15.95 | 19 |
| 11.05 | 99 | 16 | 15 |
| 11.1 | 99 | 16.05 | 14 |
| 11.15 | 101 | 16.1 | 11 |
| 11.2 | 109 | 16.15 | 12 |
| 11.25 | 106 | 16.2 | 9 |
| 11.3 | 100 | 16.25 | 11 |
| 11.35 | 108 | 16.3 | 14 |
| 11.4 | 99 | 16.35 | 11 |
| 11.45 | 109 | 16.4 | 11 |
| 11.5 | 95 | 16.45 | 14 |
| 11.55 | 108 | 16.5 | 10 |
| 11.6 | 120 | 16.55 | 9 |
| 11.65 | 84 | 16.6 | 12 |
| 11.7 | 94 | 16.65 | 12 |
| 11.75 | 106 | 16.7 | 10 |
| 11.8 | 111 | 16.75 | 6 |
| 11.85 | 89 | 16.8 | 14 |
| 11.9 | 112 | 16.85 | 9 |
| 11.95 | 109 | 16.9 | 9 |
| 12 | 108 | 16.95 | 15 |
| 12.05 | 116 | 17 | 11 |
| 12.1 | 104 | 17.05 | 5 |
| 12.15 | 121 | 17.1 | 8 |
| 12.2 | 113 | 17.15 | 14 |
| 12.25 | 114 | 17.2 | 9 |
| 12.3 | 108 | 17.25 | 8 |
| 12.35 | 99 | 17.3 | 12 |
| 12.4 | 106 | 17.35 | 6 |
| 12.45 | 106 | 17.4 | 4 |
| 12.5 | 102 | 17.45 | 11 |
| 12.55 | 98 | 17.5 | 13 |
| 12.6 | 123 | 17.55 | 7 |
| 12.65 | 82 | 17.6 | 12 |
| 12.7 | 77 | 17.65 | 15 |
| 12.75 | 109 | 17.7 | 11 |
| 12.8 | 103 | 17.75 | 9 |
| 12.85 | 114 | 17.8 | 11 |
| 12.9 | 110 | 17.85 | 5 |
| 12.95 | 88 | 17.9 | 12 |
| 13 | 100 | 17.95 | 15 |
| 13.05 | 85 | 18 | 15 |
| 13.1 | 102 | 18.05 | 9 |
| 13.15 | 129 | 18.1 | 15 |
| 13.2 | 115 | 18.15 | 13 |
| 13.25 | 99 | 18.2 | 11 |
| 13.3 | 113 | 18.25 | 11 |
| 13.35 | 94 | 18.3 | 16 |
| 13.4 | 99 | 18.35 | 16 |
| 13.45 | 79 | 18.4 | 18 |
| 13.5 | 95 | 18.45 | 13 |
| 13.55 | 105 | 18.5 | 15 |
| 13.6 | 93 | 18.55 | 12 |
| 13.65 | 79 | 18.6 | 10 |
| 13.7 | 86 | 18.65 | 12 |
| 13.75 | 89 | 18.7 | 14 |
| 13.8 | 84 | 18.75 | 7 |
| 13.85 | 96 | 18.8 | 7 |
| 13.9 | 79 | 18.85 | 5 |
| 13.95 | 72 | 18.9 | 9 |
| 14 | 96 | 18.95 | 7 |
| 14.05 | 89 | 19 | 16 |
| 14.1 | 78 | 19.05 | 8 |
| 14.15 | 99 | 19.1 | 9 |
| 14.2 | 85 | 19.15 | 11 |
| 14.25 | 81 | 19.2 | 10 |
| 14.3 | 58 | 19.25 | 15 |
| 14.35 | 72 | 19.3 | 8 |
| 14.4 | 67 | 19.35 | 8 |
| 14.45 | 67 | 19.4 | 8 |
| 14.5 | 76 | 19.45 | 5 |
| 14.55 | 69 | 19.5 | 4 |
| 14.6 | 57 | 19.55 | 6 |
| 14.65 | 62 | 19.6 | 10 |
| 14.7 | 68 | 19.65 | 10 |
| 14.75 | 73 | 19.7 | 6 |
| 14.8 | 64 | 19.75 | 5 |
| 14.85 | 60 | 19.8 | 3 |
| 14.9 | 55 | 19.85 | 8 |
| 14.95 | 57 | 19.9 | 11 |
| 15 | 41 | 19.95 | 7 |
| 15.05 | 73 | 20 | 10 |
| 15.1 | 68 | 20.05 | 9 |
| 15.15 | 49 | 20.1 | 7 |
| 15.2 | 66 | 20.15 | 7 |
| 15.25 | 47 | 20.2 | 11 |
| 15.3 | 68 | 20.25 | 15 |
| 15.35 | 53 | 20.3 | 8 |
| 15.4 | 51 | 20.35 | 6 |
| 15.45 | 56 | 20.4 | 7 |
| 15.5 | 63 | 20.45 | 7 |
| 15.55 | 60 | 20.5 | 9 |
| 15.6 | 44 | 20.55 | 16 |
| 15.65 | 44 | 20.6 | 6 |
| 15.7 | 43 | 20.65 | 11 |
| 15.75 | 47 | 20.7 | 12 |
| 15.8 | 41 | 20.75 | 7 |
| 15.85 | 50 | 20.8 | 5 |
| 15.9 | 45 | 20.85 | 7 |
| 15.95 | 44 | 20.9 | 4 |
| 16 | 41 | 20.95 | 5 |
| 16.05 | 50 | 21 | 6 |
| 16.1 | 52 | 21.05 | 6 |
| 16.15 | 39 | 21.1 | 12 |
| 16.2 | 40 | 21.15 | 6 |
| 16.25 | 40 | 21.2 | 6 |
| 16.3 | 35 | 21.25 | 10 |
| 16.35 | 37 | 21.3 | 5 |
| 16.4 | 47 | 21.35 | 4 |
| 16.45 | 45 | 21.4 | 7 |
| 16.5 | 38 | 21.45 | 8 |
| 16.55 | 45 | 21.5 | 9 |
| 16.6 | 43 | 21.55 | 4 |
| 16.65 | 46 | 21.6 | 3 |
| 16.7 | 52 | 21.65 | 10 |
| 16.75 | 35 | 21.7 | 10 |
| 16.8 | 36 | 21.75 | 7 |
| 16.85 | 41 | 21.8 | 7 |
| 16.9 | 39 | 21.85 | 5 |
| 16.95 | 38 | 21.9 | 10 |
| 17 | 32 | 21.95 | 6 |
| 17.05 | 42 | 22 | 8 |
| 17.1 | 42 | 22.05 | 9 |
| 17.15 | 41 | 22.1 | 10 |
| 17.2 | 37 | 22.15 | 9 |
| 17.25 | 31 | 22.2 | 9 |
| 17.3 | 30 | 22.25 | 6 |
| 17.35 | 36 | 22.3 | 9 |
| 17.4 | 30 | 22.35 | 5 |
| 17.45 | 33 | 22.4 | 5 |
| 17.5 | 24 | 22.45 | 4 |
| 17.55 | 25 | 22.5 | 11 |
| 17.6 | 23 | 22.55 | 5 |
| 17.65 | 25 | 22.6 | 11 |
| 17.7 | 28 | 22.65 | 9 |
| 17.75 | 27 | 22.7 | 4 |
| 17.8 | 20 | 22.75 | 7 |
| 17.85 | 36 | 22.8 | 7 |
| 17.9 | 31 | 22.85 | 10 |
| 17.95 | 27 | 22.9 | 6 |
| 18 | 32 | 22.95 | 3 |
| 18.05 | 20 | 23 | 10 |
| 18.1 | 27 | 23.05 | 11 |
| 18.15 | 27 | 23.1 | 10 |
| 18.2 | 34 | 23.15 | 16 |
| 18.25 | 22 | 23.2 | 3 |
| 18.3 | 22 | 23.25 | 6 |
| 18.35 | 25 | 23.3 | 5 |
| 18.4 | 26 | 23.35 | 6 |
| 18.45 | 26 | 23.4 | 8 |
| 18.5 | 32 | 23.45 | 10 |
| 18.55 | 29 | 23.5 | 11 |
| 18.6 | 18 | 23.55 | 8 |
| 18.65 | 19 | 23.6 | 5 |
| 18.7 | 33 | 23.65 | 10 |
| 18.75 | 24 | 23.7 | 9 |
| 18.8 | 19 | 23.75 | 11 |
| 18.85 | 26 | 23.8 | 9 |
| 18.9 | 28 | 23.85 | 9 |
| 18.95 | 30 | 23.9 | 12 |
| 19 | 16 | 23.95 | 4 |
| 19.05 | 16 | 24 | 7 |
| 19.1 | 26 | 24.05 | 6 |
| 19.15 | 24 | 24.1 | 6 |
| 19.2 | 22 | 24.15 | 6 |
| 19.25 | 16 | 24.2 | 7 |
| 19.3 | 21 | 24.25 | 7 |
| 19.35 | 29 | 24.3 | 4 |
| 19.4 | 23 | 24.35 | 6 |
| 19.45 | 16 | 24.4 | 7 |
| 19.5 | 22 | 24.45 | 6 |
| 19.55 | 18 | 24.5 | 3 |
| 19.6 | 24 | 24.55 | 7 |
| 19.65 | 16 | 24.6 | 7 |
| 19.7 | 21 | 24.65 | 6 |
| 19.75 | 32 | 24.7 | 9 |
| 19.8 | 16 | 24.75 | 11 |
| 19.85 | 17 | 24.8 | 5 |
| 19.9 | 19 | 24.85 | 7 |
| 19.95 | 10 | 24.9 | 7 |
| 20 | 12 | 24.95 | 7 |
| 20.05 | 14 | 25 | 9 |
| 20.1 | 21 | 25.05 | 4 |
| 20.15 | 29 | 25.1 | 10 |
| 20.2 | 31 | 25.15 | 11 |
| 20.25 | 51 | 25.2 | 11 |
| 20.3 | 52 | 25.25 | 10 |
| 20.35 | 30 | 25.3 | 4 |
| 20.4 | 56 | 25.35 | 6 |
| 20.45 | 76 | 25.4 | 6 |
| 20.5 | 99 | 25.45 | 6 |
| 20.55 | 79 | 25.5 | 7 |
| 20.6 | 54 | 25.55 | 7 |
| 20.65 | 40 | 25.6 | 7 |
| 20.7 | 15 | 25.65 | 7 |
| 20.75 | 28 | 25.7 | 9 |
| 20.8 | 13 | 25.75 | 8 |
| 20.85 | 21 | 25.8 | 4 |
| 20.9 | 16 | 25.85 | 6 |
| 20.95 | 13 | 25.9 | 9 |
| 21 | 11 | 25.95 | 12 |
| 21.05 | 11 | 26 | 11 |
| 21.1 | 9 | 26.05 | 11 |
| 21.15 | 11 | 26.1 | 7 |
| 21.2 | 17 | 26.15 | 6 |
| 21.25 | 13 | 26.2 | 10 |
| 21.3 | 20 | 26.25 | 8 |
| 21.35 | 18 | 26.3 | 6 |
| 21.4 | 16 | 26.35 | 8 |
| 21.45 | 14 | 26.4 | 9 |
| 21.5 | 6 | 26.45 | 12 |
| 21.55 | 12 | 26.5 | 3 |
| 21.6 | 16 | 26.55 | 7 |
| 21.65 | 18 | 26.6 | 8 |
| 21.7 | 13 | 26.65 | 10 |
| 21.75 | 15 | 26.7 | 7 |
| 21.8 | 12 | 26.75 | 7 |
| 21.85 | 14 | 26.8 | 6 |
| 21.9 | 22 | 26.85 | 7 |
| 21.95 | 21 | 26.9 | 6 |
| 22 | 18 | 26.95 | 8 |
| 22.05 | 18 | 27 | 8 |
| 22.1 | 21 | 27.05 | 6 |
| 22.15 | 14 | 27.1 | 8 |
| 22.2 | 20 | 27.15 | 10 |
| 22.25 | 19 | 27.2 | 8 |
| 22.3 | 12 | 27.25 | 8 |
| 22.35 | 15 | 27.3 | 10 |
| 22.4 | 18 | 27.35 | 9 |
| 22.45 | 19 | 27.4 | 7 |
| 22.5 | 25 | 27.45 | 10 |
| 22.55 | 13 | 27.5 | 7 |
| 22.6 | 13 | 27.55 | 6 |
| 22.65 | 28 | 27.6 | 8 |
| 22.7 | 22 | 27.65 | 6 |
| 22.75 | 31 | 27.7 | 9 |
| 22.8 | 45 | 27.75 | 8 |
| 22.85 | 76 | 27.8 | 5 |
| 22.9 | 88 | 27.85 | 6 |
| 22.95 | 107 | 27.9 | 7 |
| 23 | 82 | 27.95 | 4 |
| 23.05 | 45 | 28 | 9 |
| 23.1 | 29 | 28.05 | 7 |
| 23.15 | 22 | 28.1 | 6 |
| 23.2 | 17 | 28.15 | 8 |
| 23.25 | 16 | 28.2 | 6 |
| 23.3 | 15 | 28.25 | 10 |
| 23.35 | 9 | 28.3 | 10 |
| 23.4 | 14 | 28.35 | 7 |
| 23.45 | 20 | 28.4 | 7 |
| 23.5 | 19 | 28.45 | 9 |
| 23.55 | 9 | 28.5 | 13 |
| 23.6 | 19 | 28.55 | 6 |
| 23.65 | 13 | 28.6 | 8 |
| 23.7 | 10 | 28.65 | 9 |
| 23.75 | 12 | 28.7 | 9 |
| 23.8 | 12 | 28.75 | 10 |
| 23.85 | 14 | 28.8 | 8 |
| 23.9 | 12 | 28.85 | 10 |
| 23.95 | 14 | 28.9 | 7 |
| 24 | 13 | 28.95 | 8 |
| 24.05 | 7 | 29 | 6 |
| 24.1 | 10 | 29.05 | 7 |
| 24.15 | 9 | 29.1 | 8 |
| 24.2 | 17 | 29.15 | 9 |
| 24.25 | 14 | 29.2 | 10 |
| 24.3 | 15 | 29.25 | 16 |
| 24.35 | 8 | 29.3 | 15 |
| 24.4 | 10 | 29.35 | 7 |
| 24.45 | 8 | 29.4 | 12 |
| 24.5 | 7 | 29.45 | 9 |
| 24.55 | 15 | 29.5 | 12 |
| 24.6 | 11 | 29.55 | 15 |
| 24.65 | 8 | 29.6 | 9 |
| 24.7 | 12 | 29.65 | 25 |
| 24.75 | 13 | 29.7 | 12 |
| 24.8 | 12 | 29.75 | 12 |
| 24.85 | 15 | 29.8 | 13 |
| 24.9 | 20 | 29.85 | 25 |
| 24.95 | 13 | 29.9 | 18 |
| 25 | 17 | 29.95 | 22 |
| 25.05 | 7 | 30 | 28 |
| 25.1 | 14 | 30.05 | 35 |
| 25.15 | 13 | 30.1 | 32 |
| 25.2 | 7 | 30.15 | 44 |
| 25.25 | 23 | 30.2 | 43 |
| 25.3 | 16 | 30.25 | 39 |
| 25.35 | 19 | 30.3 | 49 |
| 25.4 | 17 | 30.35 | 50 |
| 25.45 | 17 | 30.4 | 39 |
| 25.5 | 16 | 30.45 | 42 |
| 25.55 | 14 | 30.5 | 29 |
| 25.6 | 16 | 30.55 | 32 |
| 25.65 | 10 | 30.6 | 30 |
| 25.7 | 12 | 30.65 | 15 |
| 25.75 | 11 | 30.7 | 21 |
| 25.8 | 13 | 30.75 | 20 |
| 25.85 | 16 | 30.8 | 13 |
| 25.9 | 19 | 30.85 | 13 |
| 25.95 | 10 | 30.9 | 18 |
| 26 | 11 | 30.95 | 12 |
| 26.05 | 13 | 31 | 19 |
| 26.1 | 16 | 31.05 | 10 |
| 26.15 | 5 | 31.1 | 13 |
| 26.2 | 14 | 31.15 | 10 |
| 26.25 | 12 | 31.2 | 15 |
| 26.3 | 12 | 31.25 | 8 |
| 26.35 | 10 | 31.3 | 11 |
| 26.4 | 8 | 31.35 | 7 |
| 26.45 | 12 | 31.4 | 10 |
| 26.5 | 7 | 31.45 | 8 |
| 26.55 | 14 | 31.5 | 12 |
| 26.6 | 15 | 31.55 | 8 |
| 26.65 | 10 | 31.6 | 11 |
| 26.7 | 10 | 31.65 | 2 |
| 26.75 | 10 | 31.7 | 10 |
| 26.8 | 13 | 31.75 | 4 |
| 26.85 | 14 | 31.8 | 8 |
| 26.9 | 13 | 31.85 | 5 |
| 26.95 | 11 | 31.9 | 6 |
| 27 | 17 | 31.95 | 8 |
| 27.05 | 20 | 32 | 11 |
| 27.1 | 18 | 32.05 | 7 |
| 27.15 | 14 | 32.1 | 10 |
| 27.2 | 13 | 32.15 | 16 |
| 27.25 | 10 | 32.2 | 10 |
| 27.3 | 18 | 32.25 | 8 |
| 27.35 | 14 | 32.3 | 2 |
| 27.4 | 13 | 32.35 | 13 |
| 27.45 | 20 | 32.4 | 11 |
| 27.5 | 14 | 32.45 | 7 |
| 27.55 | 11 | 32.5 | 10 |
| 27.6 | 15 | 32.55 | 6 |
| 27.65 | 9 | 32.6 | 14 |
| 27.7 | 15 | 32.65 | 17 |
| 27.75 | 9 | 32.7 | 19 |
| 27.8 | 10 | 32.75 | 19 |
| 27.85 | 8 | 32.8 | 14 |
| 27.9 | 11 | 32.85 | 18 |
| 27.95 | 9 | 32.9 | 11 |
| 28 | 9 | 32.95 | 6 |
| 28.05 | 14 | 33 | 12 |
| 28.1 | 19 | 33.05 | 9 |
| 28.15 | 15 | 33.1 | 10 |
| 28.2 | 14 | 33.15 | 9 |
| 28.25 | 16 | 33.2 | 7 |
| 28.3 | 24 | 33.25 | 10 |
| 28.35 | 38 | 33.3 | 7 |
| 28.4 | 40 | 33.35 | 9 |
| 28.45 | 57 | 33.4 | 2 |
| 28.5 | 47 | 33.45 | 7 |
| 28.55 | 29 | 33.5 | 8 |
| 28.6 | 33 | 33.55 | 4 |
| 28.65 | 33 | 33.6 | 6 |
| 28.7 | 20 | 33.65 | 6 |
| 28.75 | 22 | 33.7 | 8 |
| 28.8 | 15 | 33.75 | 8 |
| 28.85 | 24 | 33.8 | 13 |
| 28.9 | 15 | 33.85 | 9 |
| 28.95 | 14 | 33.9 | 11 |
| 29 | 9 | 33.95 | 14 |
| 29.05 | 13 | 34 | 6 |
| 29.1 | 15 | 34.05 | 8 |
| 29.15 | 13 | 34.1 | 6 |
| 29.2 | 12 | 34.15 | 10 |
| 29.25 | 22 | 34.2 | 7 |
| 29.3 | 35 | 34.25 | 13 |
| 29.35 | 38 | 34.3 | 12 |
| 29.4 | 40 | 34.35 | 9 |
| 29.45 | 26 | 34.4 | 10 |
| 29.5 | 14 | 34.45 | 12 |
| 29.55 | 18 | 34.5 | 19 |
| 29.6 | 13 | 34.55 | 14 |
| 29.65 | 17 | 34.6 | 7 |
| 29.7 | 24 | 34.65 | 14 |
| 29.75 | 24 | 34.7 | 10 |
| 29.8 | 22 | 34.75 | 16 |
| 29.85 | 23 | 34.8 | 21 |
| 29.9 | 19 | 34.85 | 19 |
| 29.95 | 28 | 34.9 | 12 |
| 30 | 31 | 34.95 | 24 |
| 30.05 | 25 | 35 | 23 |
| 30.1 | 31 | 35.05 | 22 |
| 30.15 | 32 | 35.1 | 31 |
| 30.2 | 33 | 35.15 | 23 |
| 30.25 | 41 | 35.2 | 50 |
| 30.3 | 40 | 35.25 | 46 |
| 30.35 | 39 | 35.3 | 51 |
| 30.4 | 30 | 35.35 | 67 |
| 30.45 | 28 | 35.4 | 62 |
| 30.5 | 25 | 35.45 | 93 |
| 30.55 | 25 | 35.5 | 89 |
| 30.6 | 17 | 35.55 | 117 |
| 30.65 | 26 | 35.6 | 125 |
| 30.7 | 13 | 35.65 | 127 |
| 30.75 | 16 | 35.7 | 133 |
| 30.8 | 19 | 35.75 | 124 |
| 30.85 | 16 | 35.8 | 113 |
| 30.9 | 19 | 35.85 | 95 |
| 30.95 | 12 | 35.9 | 76 |
| 31 | 11 | 35.95 | 62 |
| 31.05 | 15 | 36 | 62 |
| 31.1 | 14 | 36.05 | 33 |
| 31.15 | 22 | 36.1 | 35 |
| 31.2 | 21 | 36.15 | 37 |
| 31.25 | 21 | 36.2 | 23 |
| 31.3 | 34 | 36.25 | 26 |
| 31.35 | 30 | 36.3 | 19 |
| 31.4 | 21 | 36.35 | 25 |
| 31.45 | 28 | 36.4 | 12 |
| 31.5 | 18 | 36.45 | 15 |
| 31.55 | 15 | 36.5 | 10 |
| 31.6 | 17 | 36.55 | 19 |
| 31.65 | 17 | 36.6 | 10 |
| 31.7 | 19 | 36.65 | 9 |
| 31.75 | 10 | 36.7 | 15 |
| 31.8 | 16 | 36.75 | 12 |
| 31.85 | 20 | 36.8 | 11 |
| 31.9 | 15 | 36.85 | 9 |
| 31.95 | 17 | 36.9 | 7 |
| 32 | 14 | 36.95 | 3 |
| 32.05 | 12 | 37 | 11 |
| 32.1 | 13 | 37.05 | 11 |
| 32.15 | 18 | 37.1 | 14 |
| 32.2 | 21 | 37.15 | 6 |
| 32.25 | 26 | 37.2 | 10 |
| 32.3 | 23 | 37.25 | 9 |
| 32.35 | 24 | 37.3 | 14 |
| 32.4 | 32 | 37.35 | 9 |
| 32.45 | 40 | 37.4 | 16 |
| 32.5 | 66 | 37.45 | 6 |
| 32.55 | 136 | 37.5 | 10 |
| 32.6 | 258 | 37.55 | 8 |
| 32.65 | 369 | 37.6 | 7 |
| 32.7 | 359 | 37.65 | 7 |
| 32.75 | 212 | 37.7 | 9 |
| 32.8 | 99 | 37.75 | 13 |
| 32.85 | 52 | 37.8 | 9 |
| 32.9 | 39 | 37.85 | 6 |
| 32.95 | 22 | 37.9 | 7 |
| 33 | 20 | 37.95 | 3 |
| 33.05 | 23 | 38 | 7 |
| 33.1 | 36 | 38.05 | 7 |
| 33.15 | 33 | 38.1 | 4 |
| 33.2 | 41 | 38.15 | 4 |
| 33.25 | 30 | 38.2 | 8 |
| 33.3 | 21 | 38.25 | 10 |
| 33.35 | 14 | 38.3 | 3 |
| 33.4 | 16 | 38.35 | 2 |
| 33.45 | 18 | 38.4 | 4 |
| 33.5 | 10 | 38.45 | 11 |
| 33.55 | 13 | 38.5 | 8 |
| 33.6 | 12 | 38.55 | 6 |
| 33.65 | 23 | 38.6 | 7 |
| 33.7 | 18 | 38.65 | 9 |
| 33.75 | 21 | 38.7 | 6 |
| 33.8 | 18 | 38.75 | 4 |
| 33.85 | 23 | 38.8 | 3 |
| 33.9 | 20 | 38.85 | 1 |
| 33.95 | 10 | 38.9 | 6 |
| 34 | 20 | 38.95 | 6 |
| 34.05 | 12 | 39 | 5 |
| 34.1 | 19 | 39.05 | 8 |
| 34.15 | 13 | 39.1 | 4 |
| 34.2 | 21 | 39.15 | 4 |
| 34.25 | 25 | 39.2 | 9 |
| 34.3 | 21 | 39.25 | 7 |
| 34.35 | 15 | 39.3 | 4 |
| 34.4 | 29 | 39.35 | 4 |
| 34.45 | 22 | 39.4 | 2 |
| 34.5 | 24 | 39.45 | 6 |
| 34.55 | 26 | 39.5 | 5 |
| 34.6 | 30 | 39.55 | 4 |
| 34.65 | 15 | 39.6 | 6 |
| 34.7 | 13 | 39.65 | 2 |
| 34.75 | 16 | 39.7 | 5 |
| 34.8 | 25 | 39.75 | 3 |
| 34.85 | 20 | 39.8 | 3 |
| 34.9 | 21 | 39.85 | 2 |
| 34.95 | 24 | 39.9 | 2 |
| 35 | 29 | 39.95 | 5 |
| 35.05 | 27 | 40 | 5 |
| 35.1 | 40 | 40.05 | 4 |
| 35.15 | 21 | 40.1 | 9 |
| 35.2 | 34 | 40.15 | 5 |
| 35.25 | 35 | 40.2 | 4 |
| 35.3 | 46 | 40.25 | 3 |
| 35.35 | 58 | 40.3 | 7 |
| 35.4 | 79 | 40.35 | 5 |
| 35.45 | 79 | 40.4 | 6 |
| 35.5 | 71 | 40.45 | 4 |
| 35.55 | 122 | 40.5 | 3 |
| 35.6 | 155 | 40.55 | 5 |
| 35.65 | 98 | 40.6 | 4 |
| 35.7 | 138 | 40.65 | 7 |
| 35.75 | 94 | 40.7 | 0 |
| 35.8 | 77 | 40.75 | 3 |
| 35.85 | 68 | 40.8 | 2 |
| 35.9 | 57 | 40.85 | 1 |
| 35.95 | 40 | 40.9 | 3 |
| 36 | 40 | 40.95 | 6 |
| 36.05 | 26 | 41 | 6 |
| 36.1 | 28 | 41.05 | 6 |
| 36.15 | 31 | 41.1 | 7 |
| 36.2 | 20 | 41.15 | 2 |
| 36.25 | 24 | 41.2 | 4 |
| 36.3 | 21 | 41.25 | 6 |
| 36.35 | 16 | 41.3 | 5 |
| 36.4 | 17 | 41.35 | 2 |
| 36.45 | 15 | 41.4 | 1 |
| 36.5 | 11 | 41.45 | 2 |
| 36.55 | 14 | 41.5 | 7 |
| 36.6 | 9 | 41.55 | 2 |
| 36.65 | 8 | 41.6 | 5 |
| 36.7 | 10 | 41.65 | 3 |
| 36.75 | 19 | 41.7 | 4 |
| 36.8 | 12 | 41.75 | 2 |
| 36.85 | 10 | 41.8 | 4 |
| 36.9 | 13 | 41.85 | 4 |
| 36.95 | 14 | 41.9 | 4 |
| 37 | 15 | 41.95 | 3 |
| 37.05 | 14 | 42 | 8 |
| 37.1 | 14 | 42.05 | 2 |
| 37.15 | 8 | 42.1 | 4 |
| 37.2 | 14 | 42.15 | 2 |
| 37.25 | 8 | 42.2 | 7 |
| 37.3 | 21 | 42.25 | 5 |
| 37.35 | 11 | 42.3 | 7 |
| 37.4 | 15 | 42.35 | 2 |
| 37.45 | 18 | 42.4 | 7 |
| 37.5 | 20 | 42.45 | 6 |
| 37.55 | 16 | 42.5 | 7 |
| 37.6 | 16 | 42.55 | 4 |
| 37.65 | 11 | 42.6 | 8 |
| 37.7 | 15 | 42.65 | 6 |
| 37.75 | 11 | 42.7 | 5 |
| 37.8 | 12 | 42.75 | 14 |
| 37.85 | 8 | 42.8 | 7 |
| 37.9 | 14 | 42.85 | 17 |
| 37.95 | 14 | 42.9 | 12 |
| 38 | 13 | 42.95 | 13 |
| 38.05 | 17 | 43 | 19 |
| 38.1 | 6 | 43.05 | 13 |
| 38.15 | 9 | 43.1 | 15 |
| 38.2 | 11 | 43.15 | 26 |
| 38.25 | 8 | 43.2 | 35 |
| 38.3 | 12 | 43.25 | 27 |
| 38.35 | 9 | 43.3 | 20 |
| 38.4 | 10 | 43.35 | 28 |
| 38.45 | 11 | 43.4 | 23 |
| 38.5 | 10 | 43.45 | 23 |
| 38.55 | 12 | 43.5 | 17 |
| 38.6 | 9 | 43.55 | 19 |
| 38.65 | 13 | 43.6 | 18 |
| 38.7 | 11 | 43.65 | 11 |
| 38.75 | 15 | 43.7 | 10 |
| 38.8 | 23 | 43.75 | 8 |
| 38.85 | 19 | 43.8 | 14 |
| 38.9 | 13 | 43.85 | 3 |
| 38.95 | 21 | 43.9 | 8 |
| 39 | 12 | 43.95 | 6 |
| 39.05 | 4 | 44 | 7 |
| 39.1 | 11 | 44.05 | 8 |
| 39.15 | 9 | 44.1 | 8 |
| 39.2 | 12 | 44.15 | 7 |
| 39.25 | 12 | 44.2 | 6 |
| 39.3 | 7 | 44.25 | 6 |
| 39.35 | 10 | 44.3 | 4 |
| 39.4 | 10 | 44.35 | 4 |
| 39.45 | 11 | 44.4 | 7 |
| 39.5 | 8 | 44.45 | 4 |
| 39.55 | 9 | 44.5 | 4 |
| 39.6 | 14 | 44.55 | 4 |
| 39.65 | 5 | 44.6 | 8 |
| 39.7 | 7 | 44.65 | 5 |
| 39.75 | 4 | 44.7 | 7 |
| 39.8 | 5 | 44.75 | 1 |
| 39.85 | 11 | 44.8 | 1 |
| 39.9 | 9 | 44.85 | 3 |
| 39.95 | 6 | 44.9 | 1 |
| 40 | 12 | 44.95 | 2 |
| 40.05 | 14 | 45 | 4 |
| 40.1 | 5 | 45.05 | 4 |
| 40.15 | 11 | 45.1 | 5 |
| 40.2 | 17 | 45.15 | 1 |
| 40.25 | 23 | 45.2 | 6 |
| 40.3 | 19 | 45.25 | 8 |
| 40.35 | 22 | 45.3 | 7 |
| 40.4 | 14 | 45.35 | 4 |
| 40.45 | 14 | 45.4 | 6 |
| 40.5 | 19 | 45.45 | 0 |
| 40.55 | 10 | 45.5 | 3 |
| 40.6 | 7 | 45.55 | 2 |
| 40.65 | 16 | 45.6 | 2 |
| 40.7 | 10 | 45.65 | 6 |
| 40.75 | 8 | 45.7 | 4 |
| 40.8 | 14 | 45.75 | 3 |
| 40.85 | 12 | 45.8 | 4 |
| 40.9 | 7 | 45.85 | 2 |
| 40.95 | 11 | 45.9 | 3 |
| 41 | 14 | 45.95 | 6 |
| 41.05 | 11 | 46 | 2 |
| 41.1 | 36 | 46.05 | 8 |
| 41.15 | 40 | 46.1 | 2 |
| 41.2 | 31 | 46.15 | 2 |
| 41.25 | 27 | 46.2 | 4 |
| 41.3 | 24 | 46.25 | 2 |
| 41.35 | 14 | 46.3 | 6 |
| 41.4 | 8 | 46.35 | 3 |
| 41.45 | 11 | 46.4 | 3 |
| 41.5 | 9 | 46.45 | 6 |
| 41.55 | 17 | 46.5 | 3 |
| 41.6 | 11 | 46.55 | 4 |
| 41.65 | 14 | 46.6 | 4 |
| 41.7 | 13 | 46.65 | 1 |
| 41.75 | 15 | 46.7 | 4 |
| 41.8 | 13 | 46.75 | 3 |
| 41.85 | 23 | 46.8 | 5 |
| 41.9 | 7 | 46.85 | 6 |
| 41.95 | 6 | 46.9 | 7 |
| 42 | 10 | 46.95 | 4 |
| 42.05 | 9 | 47 | 2 |
| 42.1 | 12 | 47.05 | 4 |
| 42.15 | 7 | 47.1 | 7 |
| 42.2 | 6 | 47.15 | 5 |
| 42.25 | 13 | 47.2 | 2 |
| 42.3 | 8 | 47.25 | 2 |
| 42.35 | 9 | 47.3 | 7 |
| 42.4 | 10 | 47.35 | 7 |
| 42.45 | 8 | 47.4 | 1 |
| 42.5 | 15 | 47.45 | 7 |
| 42.55 | 12 | 47.5 | 2 |
| 42.6 | 8 | 47.55 | 2 |
| 42.65 | 10 | 47.6 | 5 |
| 42.7 | 4 | 47.65 | 3 |
| 42.75 | 9 | 47.7 | 4 |
| 42.8 | 17 | 47.75 | 1 |
| 42.85 | 15 | 47.8 | 2 |
| 42.9 | 13 | 47.85 | 4 |
| 42.95 | 19 | 47.9 | 3 |
| 43 | 12 | 47.95 | 3 |
| 43.05 | 10 | 48 | 0 |
| 43.1 | 21 | 48.05 | 9 |
| 43.15 | 22 | 48.1 | 4 |
| 43.2 | 18 | 48.15 | 7 |
| 43.25 | 28 | 48.2 | 4 |
| 43.3 | 29 | 48.25 | 3 |
| 43.35 | 19 | 48.3 | 7 |
| 43.4 | 16 | 48.35 | 2 |
| 43.45 | 25 | 48.4 | 3 |
| 43.5 | 24 | 48.45 | 2 |
| 43.55 | 19 | 48.5 | 5 |
| 43.6 | 20 | 48.55 | 4 |
| 43.65 | 19 | 48.6 | 5 |
| 43.7 | 12 | 48.65 | 5 |
| 43.75 | 14 | 48.7 | 1 |
| 43.8 | 16 | 48.75 | 1 |
| 43.85 | 12 | 48.8 | 4 |
| 43.9 | 10 | 48.85 | 3 |
| 43.95 | 11 | 48.9 | 3 |
| 44 | 9 | 48.95 | 2 |
| 44.05 | 11 | 49 | 1 |
| 44.1 | 7 | 49.05 | 5 |
| 44.15 | 12 | 49.1 | 9 |
| 44.2 | 5 | 49.15 | 3 |
| 44.25 | 9 | 49.2 | 7 |
| 44.3 | 11 | 49.25 | 4 |
| 44.35 | 7 | 49.3 | 1 |
| 44.4 | 8 | 49.35 | 3 |
| 44.45 | 7 | 49.4 | 3 |
| 44.5 | 5 | 49.45 | 3 |
| 44.55 | 10 | 49.5 | 5 |
| 44.6 | 7 | 49.55 | 2 |
| 44.65 | 13 | 49.6 | 5 |
| 44.7 | 5 | 49.65 | 3 |
| 44.75 | 6 | 49.7 | 9 |
| 44.8 | 6 | 49.75 | 2 |
| 44.85 | 10 | 49.8 | 3 |
| 44.9 | 9 | 49.85 | 4 |
| 44.95 | 13 | 49.9 | 0 |
| 45 | 8 | 49.95 | 3 |
| 45.05 | 9 | 50 | 7 |
| 45.1 | 12 | 50.05 | 3 |
| 45.15 | 8 | 50.1 | 7 |
| 45.2 | 8 | 50.15 | 4 |
| 45.25 | 9 | 50.2 | 5 |
| 45.3 | 5 | 50.25 | 1 |
| 45.35 | 10 | 50.3 | 1 |
| 45.4 | 9 | 50.35 | 2 |
| 45.45 | 7 | 50.4 | 2 |
| 45.5 | 3 | 50.45 | 6 |
| 45.55 | 9 | 50.5 | 7 |
| 45.6 | 6 | 50.55 | 0 |
| 45.65 | 9 | 50.6 | 4 |
| 45.7 | 8 | 50.65 | 3 |
| 45.75 | 5 | 50.7 | 3 |
| 45.8 | 8 | 50.75 | 2 |
| 45.85 | 10 | 50.8 | 8 |
| 45.9 | 8 | 50.85 | 6 |
| 45.95 | 10 | 50.9 | 5 |
| 46 | 8 | 50.95 | 3 |
| 46.05 | 7 | 51 | 0 |
| 46.1 | 6 | 51.05 | 3 |
| 46.15 | 4 | 51.1 | 7 |
| 46.2 | 8 | 51.15 | 2 |
| 46.25 | 4 | 51.2 | 4 |
| 46.3 | 6 | 51.25 | 0 |
| 46.35 | 11 | 51.3 | 1 |
| 46.4 | 7 | 51.35 | 3 |
| 46.45 | 7 | 51.4 | 3 |
| 46.5 | 6 | 51.45 | 4 |
| 46.55 | 10 | 51.5 | 7 |
| 46.6 | 17 | 51.55 | 1 |
| 46.65 | 20 | 51.6 | 4 |
| 46.7 | 13 | 51.65 | 4 |
| 46.75 | 29 | 51.7 | 2 |
| 46.8 | 31 | 51.75 | 4 |
| 46.85 | 58 | 51.8 | 1 |
| 46.9 | 39 | 51.85 | 5 |
| 46.95 | 51 | 51.9 | 3 |
| 47 | 27 | 51.95 | 2 |
| 47.05 | 16 | 52 | 6 |
| 47.1 | 21 | 52.05 | 4 |
| 47.15 | 8 | 52.1 | 2 |
| 47.2 | 6 | 52.15 | 6 |
| 47.25 | 4 | 52.2 | 4 |
| 47.3 | 6 | 52.25 | 1 |
| 47.35 | 7 | 52.3 | 6 |
| 47.4 | 10 | 52.35 | 3 |
| 47.45 | 13 | 52.4 | 3 |
| 47.5 | 3 | 52.45 | 6 |
| 47.55 | 8 | 52.5 | 1 |
| 47.6 | 10 | 52.55 | 5 |
| 47.65 | 6 | 52.6 | 6 |
| 47.7 | 11 | 52.65 | 4 |
| 47.75 | 9 | 52.7 | 5 |
| 47.8 | 9 | 52.75 | 4 |
| 47.85 | 9 | 52.8 | 5 |
| 47.9 | 7 | 52.85 | 4 |
| 47.95 | 4 | 52.9 | 2 |
| 48 | 8 | 52.95 | 4 |
| 48.05 | 10 | 53 | 5 |
| 48.1 | 12 | 53.05 | 7 |
| 48.15 | 8 | 53.1 | 8 |
| 48.2 | 7 | 53.15 | 2 |
| 48.25 | 4 | 53.2 | 10 |
| 48.3 | 5 | 53.25 | 6 |
| 48.35 | 6 | 53.3 | 5 |
| 48.4 | 13 | 53.35 | 5 |
| 48.45 | 6 | 53.4 | 13 |
| 48.5 | 12 | 53.45 | 13 |
| 48.55 | 5 | 53.5 | 8 |
| 48.6 | 6 | 53.55 | 13 |
| 48.65 | 7 | 53.6 | 10 |
| 48.7 | 7 | 53.65 | 15 |
| 48.75 | 3 | 53.7 | 12 |
| 48.8 | 6 | 53.75 | 17 |
| 48.85 | 8 | 53.8 | 11 |
| 48.9 | 7 | 53.85 | 12 |
| 48.95 | 9 | 53.9 | 9 |
| 49 | 8 | 53.95 | 15 |
| 49.05 | 11 | 54 | 7 |
| 49.1 | 11 | 54.05 | 10 |
| 49.15 | 3 | 54.1 | 10 |
| 49.2 | 14 | 54.15 | 9 |
| 49.25 | 5 | 54.2 | 7 |
| 49.3 | 9 | 54.25 | 12 |
| 49.35 | 8 | 54.3 | 6 |
| 49.4 | 5 | 54.35 | 7 |
| 49.45 | 7 | 54.4 | 5 |
| 49.5 | 11 | 54.45 | 4 |
| 49.55 | 8 | 54.5 | 7 |
| 49.6 | 7 | 54.55 | 13 |
| 49.65 | 11 | 54.6 | 7 |
| 49.7 | 8 | 54.65 | 2 |
| 49.75 | 6 | 54.7 | 4 |
| 49.8 | 7 | 54.75 | 5 |
| 49.85 | 3 | 54.8 | 4 |
| 49.9 | 14 | 54.85 | 12 |
| 49.95 | 7 | 54.9 | 6 |
| 50 | 7 | 54.95 | 7 |
| 50.05 | 7 | 55 | 4 |
| 50.1 | 10 | 55.05 | 2 |
| 50.15 | 8 | 55.1 | 6 |
| 50.2 | 10 | 55.15 | 4 |
| 50.25 | 13 | 55.2 | 1 |
| 50.3 | 10 | 55.25 | 2 |
| 50.35 | 5 | 55.3 | 8 |
| 50.4 | 8 | 55.35 | 3 |
| 50.45 | 12 | 55.4 | 6 |
| 50.5 | 10 | 55.45 | 4 |
| 50.55 | 9 | 55.5 | 6 |
| 50.6 | 10 | 55.55 | 8 |
| 50.65 | 7 | 55.6 | 5 |
| 50.7 | 5 | 55.65 | 6 |
| 50.75 | 3 | 55.7 | 6 |
| 50.8 | 7 | 55.75 | 2 |
| 50.85 | 5 | 55.8 | 7 |
| 50.9 | 8 | 55.85 | 5 |
| 50.95 | 8 | 55.9 | 9 |
| 51 | 7 | 55.95 | 8 |
| 51.05 | 9 | 56 | 6 |
| 51.1 | 3 | 56.05 | 7 |
| 51.15 | 6 | 56.1 | 10 |
| 51.2 | 8 | 56.15 | 6 |
| 51.25 | 5 | 56.2 | 8 |
| 51.3 | 12 | 56.25 | 7 |
| 51.35 | 8 | 56.3 | 3 |
| 51.4 | 11 | 56.35 | 16 |
| 51.45 | 10 | 56.4 | 8 |
| 51.5 | 6 | 56.45 | 4 |
| 51.55 | 11 | 56.5 | 7 |
| 51.6 | 5 | 56.55 | 5 |
| 51.65 | 16 | 56.6 | 8 |
| 51.7 | 8 | 56.65 | 10 |
| 51.75 | 6 | 56.7 | 12 |
| 51.8 | 12 | 56.75 | 10 |
| 51.85 | 7 | 56.8 | 13 |
| 51.9 | 3 | 56.85 | 13 |
| 51.95 | 6 | 56.9 | 18 |
| 52 | 10 | 56.95 | 18 |
| 52.05 | 8 | 57 | 23 |
| 52.1 | 5 | 57.05 | 34 |
| 52.15 | 5 | 57.1 | 22 |
| 52.2 | 5 | 57.15 | 29 |
| 52.25 | 5 | 57.2 | 43 |
| 52.3 | 6 | 57.25 | 46 |
| 52.35 | 8 | 57.3 | 33 |
| 52.4 | 10 | 57.35 | 37 |
| 52.45 | 8 | 57.4 | 51 |
| 52.5 | 10 | 57.45 | 28 |
| 52.55 | 10 | 57.5 | 24 |
| 52.6 | 10 | 57.55 | 23 |
| 52.65 | 8 | 57.6 | 15 |
| 52.7 | 19 | 57.65 | 33 |
| 52.75 | 28 | 57.7 | 15 |
| 52.8 | 26 | 57.75 | 14 |
| 52.85 | 18 | 57.8 | 11 |
| 52.9 | 21 | 57.85 | 14 |
| 52.95 | 21 | 57.9 | 14 |
| 53 | 17 | 57.95 | 19 |
| 53.05 | 8 | 58 | 10 |
| 53.1 | 10 | 58.05 | 8 |
| 53.15 | 14 | 58.1 | 5 |
| 53.2 | 12 | 58.15 | 10 |
| 53.25 | 9 | 58.2 | 8 |
| 53.3 | 11 | 58.25 | 6 |
| 53.35 | 9 | 58.3 | 9 |
| 53.4 | 9 | 58.35 | 5 |
| 53.45 | 15 | 58.4 | 6 |
| 53.5 | 11 | 58.45 | 6 |
| 53.55 | 21 | 58.5 | 6 |
| 53.6 | 14 | 58.55 | 4 |
| 53.65 | 15 | 58.6 | 8 |
| 53.7 | 19 | 58.65 | 7 |
| 53.75 | 21 | 58.7 | 10 |
| 53.8 | 9 | 58.75 | 7 |
| 53.85 | 18 | 58.8 | 7 |
| 53.9 | 14 | 58.85 | 8 |
| 53.95 | 10 | 58.9 | 8 |
| 54 | 22 | 58.95 | 8 |
| 54.05 | 10 | 59 | 2 |
| 54.1 | 17 | 59.05 | 3 |
| 54.15 | 11 | 59.1 | 1 |
| 54.2 | 9 | 59.15 | 6 |
| 54.25 | 12 | 59.2 | 5 |
| 54.3 | 7 | 59.25 | 5 |
| 54.35 | 13 | 59.3 | 5 |
| 54.4 | 14 | 59.35 | 3 |
| 54.45 | 7 | 59.4 | 5 |
| 54.5 | 6 | 59.45 | 7 |
| 54.55 | 4 | 59.5 | 3 |
| 54.6 | 10 | 59.55 | 5 |
| 54.65 | 8 | 59.6 | 9 |
| 54.7 | 12 | 59.65 | 5 |
| 54.75 | 6 | 59.7 | 6 |
| 54.8 | 12 | 59.75 | 1 |
| 54.85 | 6 | 59.8 | 6 |
| 54.9 | 10 | 59.85 | 1 |
| 54.95 | 5 | 59.9 | 2 |
| 55 | 11 | 59.95 | 3 |
| 55.05 | 7 | 60 | 5 |
| 55.1 | 10 | 60.05 | 5 |
| 55.15 | 10 | 60.1 | 3 |
| 55.2 | 9 | 60.15 | 2 |
| 55.25 | 10 | 60.2 | 9 |
| 55.3 | 7 | 60.25 | 5 |
| 55.35 | 11 | 60.3 | 4 |
| 55.4 | 10 | 60.35 | 7 |
| 55.45 | 8 | 60.4 | 6 |
| 55.5 | 11 | 60.45 | 7 |
| 55.55 | 5 | 60.5 | 4 |
| 55.6 | 10 | 60.55 | 4 |
| 55.65 | 9 | 60.6 | 6 |
| 55.7 | 13 | 60.65 | 5 |
| 55.75 | 9 | 60.7 | 5 |
| 55.8 | 10 | 60.75 | 5 |
| 55.85 | 14 | 60.8 | 7 |
| 55.9 | 11 | 60.85 | 4 |
| 55.95 | 15 | 60.9 | 5 |
| 56 | 4 | 60.95 | 3 |
| 56.05 | 8 | 61 | 0 |
| 56.1 | 13 | 61.05 | 5 |
| 56.15 | 8 | 61.1 | 4 |
| 56.2 | 11 | 61.15 | 2 |
| 56.25 | 8 | 61.2 | 5 |
| 56.3 | 9 | 61.25 | 3 |
| 56.35 | 15 | 61.3 | 6 |
| 56.4 | 7 | 61.35 | 7 |
| 56.45 | 14 | 61.4 | 3 |
| 56.5 | 17 | 61.45 | 6 |
| 56.55 | 12 | 61.5 | 3 |
| 56.6 | 14 | 61.55 | 11 |
| 56.65 | 9 | 61.6 | 3 |
| 56.7 | 16 | 61.65 | 4 |
| 56.75 | 14 | 61.7 | 7 |
| 56.8 | 17 | 61.75 | 8 |
| 56.85 | 13 | 61.8 | 9 |
| 56.9 | 13 | 61.85 | 7 |
| 56.95 | 20 | 61.9 | 7 |
| 57 | 20 | 61.95 | 12 |
| 57.05 | 23 | 62 | 10 |
| 57.1 | 30 | 62.05 | 8 |
| 57.15 | 23 | 62.1 | 7 |
| 57.2 | 32 | 62.15 | 12 |
| 57.25 | 34 | 62.2 | 7 |
| 57.3 | 29 | 62.25 | 11 |
| 57.35 | 25 | 62.3 | 13 |
| 57.4 | 28 | 62.35 | 20 |
| 57.45 | 25 | 62.4 | 13 |
| 57.5 | 25 | 62.45 | 23 |
| 57.55 | 27 | 62.5 | 13 |
| 57.6 | 23 | 62.55 | 25 |
| 57.65 | 18 | 62.6 | 23 |
| 57.7 | 13 | 62.65 | 39 |
| 57.75 | 21 | 62.7 | 27 |
| 57.8 | 18 | 62.75 | 41 |
| 57.85 | 14 | 62.8 | 52 |
| 57.9 | 12 | 62.85 | 52 |
| 57.95 | 13 | 62.9 | 56 |
| 58 | 12 | 62.95 | 48 |
| 58.05 | 19 | 63 | 52 |
| 58.1 | 23 | 63.05 | 37 |
| 58.15 | 34 | 63.1 | 43 |
| 58.2 | 52 | 63.15 | 46 |
| 58.25 | 102 | 63.2 | 50 |
| 58.3 | 65 | 63.25 | 27 |
| 58.35 | 48 | 63.3 | 30 |
| 58.4 | 60 | 63.35 | 20 |
| 58.45 | 49 | 63.4 | 21 |
| 58.5 | 23 | 63.45 | 12 |
| 58.55 | 10 | 63.5 | 18 |
| 58.6 | 15 | 63.55 | 9 |
| 58.65 | 11 | 63.6 | 9 |
| 58.7 | 8 | 63.65 | 16 |
| 58.75 | 6 | 63.7 | 12 |
| 58.8 | 6 | 63.75 | 6 |
| 58.85 | 12 | 63.8 | 7 |
| 58.9 | 13 | 63.85 | 9 |
| 58.95 | 11 | 63.9 | 7 |
| 59 | 10 | 63.95 | 8 |
| 59.05 | 10 | 64 | 9 |
| 59.1 | 12 | 64.05 | 8 |
| 59.15 | 5 | 64.1 | 3 |
| 59.2 | 9 | 64.15 | 6 |
| 59.25 | 8 | 64.2 | 5 |
| 59.3 | 11 | 64.25 | 6 |
| 59.35 | 7 | 64.3 | 6 |
| 59.4 | 10 | 64.35 | 4 |
| 59.45 | 14 | 64.4 | 2 |
| 59.5 | 9 | 64.45 | 7 |
| 59.55 | 17 | 64.5 | 6 |
| 59.6 | 12 | 64.55 | 3 |
| 59.65 | 6 | 64.6 | 5 |
| 59.7 | 11 | 64.65 | 7 |
| 59.75 | 6 | 64.7 | 3 |
| 59.8 | 9 | 64.75 | 2 |
| 59.85 | 7 | 64.8 | 9 |
| 59.9 | 10 | 64.85 | 7 |
| 59.95 | 7 | 64.9 | 2 |
| 60 | 10 | 64.95 | 3 |
| 60.05 | 10 | 65 | 5 |
| 60.1 | 4 | 65.05 | 6 |
| 60.15 | 11 | 65.1 | 4 |
| 60.2 | 8 | 65.15 | 5 |
| 60.25 | 7 | 65.2 | 6 |
| 60.3 | 6 | 65.25 | 4 |
| 60.35 | 13 | 65.3 | 4 |
| 60.4 | 12 | 65.35 | 6 |
| 60.45 | 6 | 65.4 | 3 |
| 60.5 | 11 | 65.45 | 3 |
| 60.55 | 5 | 65.5 | 5 |
| 60.6 | 6 | 65.55 | 1 |
| 60.65 | 5 | 65.6 | 3 |
| 60.7 | 5 | 65.65 | 3 |
| 60.75 | 11 | 65.7 | 2 |
| 60.8 | 7 | 65.75 | 3 |
| 60.85 | 11 | 65.8 | 3 |
| 60.9 | 8 | 65.85 | 4 |
| 60.95 | 8 | 65.9 | 7 |
| 61 | 14 | 65.95 | 5 |
| 61.05 | 11 | 66 | 4 |
| 61.1 | 8 | 66.05 | 3 |
| 61.15 | 4 | 66.1 | 2 |
| 61.2 | 10 | 66.15 | 6 |
| 61.25 | 7 | 66.2 | 5 |
| 61.3 | 8 | 66.25 | 3 |
| 61.35 | 7 | 66.3 | 1 |
| 61.4 | 10 | 66.35 | 5 |
| 61.45 | 7 | 66.4 | 4 |
| 61.5 | 7 | 66.45 | 2 |
| 61.55 | 18 | 66.5 | 5 |
| 61.6 | 10 | 66.55 | 3 |
| 61.65 | 8 | 66.6 | 4 |
| 61.7 | 11 | 66.65 | 5 |
| 61.75 | 7 | 66.7 | 2 |
| 61.8 | 9 | 66.75 | 5 |
| 61.85 | 6 | 66.8 | 3 |
| 61.9 | 13 | 66.85 | 6 |
| 61.95 | 10 | 66.9 | 4 |
| 62 | 12 | 66.95 | 7 |
| 62.05 | 12 | 67 | 2 |
| 62.1 | 9 | 67.05 | 2 |
| 62.15 | 15 | 67.1 | 5 |
| 62.2 | 11 | 67.15 | 2 |
| 62.25 | 10 | 67.2 | 5 |
| 62.3 | 17 | 67.25 | 2 |
| 62.35 | 19 | 67.3 | 2 |
| 62.4 | 19 | 67.35 | 3 |
| 62.45 | 16 | 67.4 | 3 |
| 62.5 | 18 | 67.45 | 1 |
| 62.55 | 22 | 67.5 | 1 |
| 62.6 | 26 | 67.55 | 2 |
| 62.65 | 27 | 67.6 | 1 |
| 62.7 | 29 | 67.65 | 2 |
| 62.75 | 37 | 67.7 | 5 |
| 62.8 | 34 | 67.75 | 6 |
| 62.85 | 46 | 67.8 | 4 |
| 62.9 | 35 | 67.85 | 7 |
| 62.95 | 41 | 67.9 | 4 |
| 63 | 44 | 67.95 | 2 |
| 63.05 | 26 | 68 | 6 |
| 63.1 | 26 | 68.05 | 2 |
| 63.15 | 33 | 68.1 | 6 |
| 63.2 | 23 | 68.15 | 5 |
| 63.25 | 26 | 68.2 | 7 |
| 63.3 | 21 | 68.25 | 5 |
| 63.35 | 21 | 68.3 | 3 |
| 63.4 | 13 | 68.35 | 3 |
| 63.45 | 12 | 68.4 | 3 |
| 63.5 | 14 | 68.45 | 5 |
| 63.55 | 7 | 68.5 | 7 |
| 63.6 | 14 | 68.55 | 2 |
| 63.65 | 7 | 68.6 | 4 |
| 63.7 | 7 | 68.65 | 8 |
| 63.75 | 13 | 68.7 | 4 |
| 63.8 | 15 | 68.75 | 7 |
| 63.85 | 8 | 68.8 | 2 |
| 63.9 | 9 | 68.85 | 4 |
| 63.95 | 5 | 68.9 | 4 |
| 64 | 8 | 68.95 | 1 |
| 64.05 | 8 | 69 | 1 |
| 64.1 | 6 | 69.05 | 4 |
| 64.15 | 6 | 69.1 | 5 |
| 64.2 | 11 | 69.15 | 3 |
| 64.25 | 9 | 69.2 | 3 |
| 64.3 | 10 | 69.25 | 0 |
| 64.35 | 9 | 69.3 | 3 |
| 64.4 | 9 | 69.35 | 3 |
| 64.45 | 10 | 69.4 | 3 |
| 64.5 | 7 | 69.45 | 5 |
| 64.55 | 9 | 69.5 | 4 |
| 64.6 | 12 | 69.55 | 2 |
| 64.65 | 10 | 69.6 | 3 |
| 64.7 | 7 | 69.65 | 2 |
| 64.75 | 11 | 69.7 | 5 |
| 64.8 | 9 | 69.75 | 1 |
| 64.85 | 5 | 69.8 | 5 |
| 64.9 | 5 | 69.85 | 2 |
| 64.95 | 5 | 69.9 | 6 |
| 65 | 2 | 69.95 | 4 |
| 65.05 | 9 | 70 | 3 |
| 65.1 | 17 | 70.05 | 2 |
| 65.15 | 4 | 70.1 | 3 |
| 65.2 | 8 | 70.15 | 4 |
| 65.25 | 8 | 70.2 | 2 |
| 65.3 | 8 | 70.25 | 5 |
| 65.35 | 9 | 70.3 | 3 |
| 65.4 | 12 | 70.35 | 5 |
| 65.45 | 5 | 70.4 | 1 |
| 65.5 | 7 | 70.45 | 5 |
| 65.55 | 7 | 70.5 | 2 |
| 65.6 | 14 | 70.55 | 4 |
| 65.65 | 8 | 70.6 | 5 |
| 65.7 | 8 | 70.65 | 3 |
| 65.75 | 6 | 70.7 | 4 |
| 65.8 | 3 | 70.75 | 3 |
| 65.85 | 4 | 70.8 | 3 |
| 65.9 | 4 | 70.85 | 7 |
| 65.95 | 9 | 70.9 | 4 |
| 66 | 9 | 70.95 | 8 |
| 66.05 | 12 | 71 | 5 |
| 66.1 | 8 | 71.05 | 9 |
| 66.15 | 6 | 71.1 | 9 |
| 66.2 | 11 | 71.15 | 5 |
| 66.25 | 11 | 71.2 | 1 |
| 66.3 | 13 | 71.25 | 1 |
| 66.35 | 11 | 71.3 | 5 |
| 66.4 | 6 | 71.35 | 7 |
| 66.45 | 4 | 71.4 | 8 |
| 66.5 | 5 | 71.45 | 9 |
| 66.55 | 6 | 71.5 | 6 |
| 66.6 | 10 | 71.55 | 4 |
| 66.65 | 6 | 71.6 | 2 |
| 66.7 | 9 | 71.65 | 4 |
| 66.75 | 5 | 71.7 | 6 |
| 66.8 | 6 | 71.75 | 8 |
| 66.85 | 7 | 71.8 | 8 |
| 66.9 | 9 | 71.85 | 7 |
| 66.95 | 1 | 71.9 | 3 |
| 67 | 9 | 71.95 | 4 |
| 67.05 | 10 | 72 | 7 |
| 67.1 | 5 | 72.05 | 8 |
| 67.15 | 5 | 72.1 | 1 |
| 67.2 | 11 | 72.15 | 1 |
| 67.25 | 6 | 72.2 | 4 |
| 67.3 | 6 | 72.25 | 1 |
| 67.35 | 7 | 72.3 | 1 |
| 67.4 | 6 | 72.35 | 5 |
| 67.45 | 2 | 72.4 | 6 |
| 67.5 | 12 | 72.45 | 4 |
| 67.55 | 4 | 72.5 | 6 |
| 67.6 | 5 | 72.55 | 3 |
| 67.65 | 11 | 72.6 | 1 |
| 67.7 | 5 | 72.65 | 3 |
| 67.75 | 6 | 72.7 | 5 |
| 67.8 | 9 | 72.75 | 1 |
| 67.85 | 11 | 72.8 | 2 |
| 67.9 | 4 | 72.85 | 4 |
| 67.95 | 9 | 72.9 | 2 |
| 68 | 6 | 72.95 | 4 |
| 68.05 | 4 | 73 | 6 |
| 68.1 | 11 | 73.05 | 6 |
| 68.15 | 5 | 73.1 | 2 |
| 68.2 | 4 | 73.15 | 3 |
| 68.25 | 10 | 73.2 | 3 |
| 68.3 | 12 | 73.25 | 1 |
| 68.35 | 15 | 73.3 | 4 |
| 68.4 | 21 | 73.35 | 2 |
| 68.45 | 14 | 73.4 | 8 |
| 68.5 | 12 | 73.45 | 4 |
| 68.55 | 14 | 73.5 | 4 |
| 68.6 | 13 | 73.55 | 1 |
| 68.65 | 14 | 73.6 | 3 |
| 68.7 | 16 | 73.65 | 2 |
| 68.75 | 5 | 73.7 | 6 |
| 68.8 | 9 | 73.75 | 2 |
| 68.85 | 7 | 73.8 | 2 |
| 68.9 | 11 | 73.85 | 5 |
| 68.95 | 3 | 73.9 | 4 |
| 69 | 5 | 73.95 | 9 |
| 69.05 | 7 | 74 | 7 |
| 69.1 | 4 | 74.05 | 5 |
| 69.15 | 5 | 74.1 | 8 |
| 69.2 | 4 | 74.15 | 7 |
| 69.25 | 5 | 74.2 | 6 |
| 69.3 | 5 | 74.25 | 7 |
| 69.35 | 5 | 74.3 | 16 |
| 69.4 | 10 | 74.35 | 9 |
| 69.45 | 10 | 74.4 | 6 |
| 69.5 | 16 | 74.45 | 13 |
| 69.55 | 9 | 74.5 | 9 |
| 69.6 | 8 | 74.55 | 8 |
| 69.65 | 8 | 74.6 | 8 |
| 69.7 | 8 | 74.65 | 7 |
| 69.75 | 6 | 74.7 | 9 |
| 69.8 | 9 | 74.75 | 7 |
| 69.85 | 9 | 74.8 | 4 |
| 69.9 | 6 | 74.85 | 8 |
| 69.95 | 8 | 74.9 | 7 |
| 70 | 5 | 74.95 | 4 |
| 70.05 | 7 | 75 | 6 |
| 70.1 | 5 | 75.05 | 5 |
| 70.15 | 4 | 75.1 | 4 |
| 70.2 | 6 | 75.15 | 2 |
| 70.25 | 6 | 75.2 | 4 |
| 70.3 | 4 | 75.25 | 4 |
| 70.35 | 9 | 75.3 | 5 |
| 70.4 | 11 | 75.35 | 10 |
| 70.45 | 11 | 75.4 | 8 |
| 70.5 | 9 | 75.45 | 2 |
| 70.55 | 9 | 75.5 | 8 |
| 70.6 | 8 | 75.55 | 8 |
| 70.65 | 6 | 75.6 | 7 |
| 70.7 | 4 | 75.65 | 4 |
| 70.75 | 8 | 75.7 | 11 |
| 70.8 | 3 | 75.75 | 4 |
| 70.85 | 10 | 75.8 | 0 |
| 70.9 | 3 | 75.85 | 6 |
| 70.95 | 8 | 75.9 | 4 |
| 71 | 8 | 75.95 | 4 |
| 71.05 | 4 | 76 | 4 |
| 71.1 | 5 | 76.05 | 6 |
| 71.15 | 5 | 76.1 | 5 |
| 71.2 | 8 | 76.15 | 2 |
| 71.25 | 6 | 76.2 | 6 |
| 71.3 | 9 | 76.25 | 4 |
| 71.35 | 7 | 76.3 | 2 |
| 71.4 | 7 | 76.35 | 4 |
| 71.45 | 7 | 76.4 | 2 |
| 71.5 | 3 | 76.45 | 6 |
| 71.55 | 4 | 76.5 | 0 |
| 71.6 | 5 | 76.55 | 2 |
| 71.65 | 5 | 76.6 | 4 |
| 71.7 | 8 | 76.65 | 4 |
| 71.75 | 5 | 76.7 | 0 |
| 71.8 | 5 | 76.75 | 2 |
| 71.85 | 9 | 76.8 | 3 |
| 71.9 | 11 | 76.85 | 4 |
| 71.95 | 5 | 76.9 | 2 |
| 72 | 3 | 76.95 | 1 |
| 72.05 | 10 | 77 | 2 |
| 72.1 | 6 | 77.05 | 2 |
| 72.15 | 11 | 77.1 | 1 |
| 72.2 | 9 | 77.15 | 3 |
| 72.25 | 9 | 77.2 | 1 |
| 72.3 | 8 | 77.25 | 1 |
| 72.35 | 3 | 77.3 | 1 |
| 72.4 | 7 | 77.35 | 4 |
| 72.45 | 8 | 77.4 | 2 |
| 72.5 | 6 | 77.45 | 2 |
| 72.55 | 9 | 77.5 | 4 |
| 72.6 | 7 | 77.55 | 0 |
| 72.65 | 5 | 77.6 | 2 |
| 72.7 | 4 | 77.65 | 1 |
| 72.75 | 7 | 77.7 | 2 |
| 72.8 | 6 | 77.75 | 2 |
| 72.85 | 6 | 77.8 | 5 |
| 72.9 | 8 | 77.85 | 2 |
| 72.95 | 6 | 77.9 | 5 |
| 73 | 4 | 77.95 | 3 |
| 73.05 | 9 | 78 | 0 |
| 73.1 | 9 | 78.05 | 3 |
| 73.15 | 17 | 78.1 | 4 |
| 73.2 | 13 | 78.15 | 2 |
| 73.25 | 11 | 78.2 | 0 |
| 73.3 | 10 | 78.25 | 3 |
| 73.35 | 7 | 78.3 | 2 |
| 73.4 | 8 | 78.35 | 3 |
| 73.45 | 8 | 78.4 | 1 |
| 73.5 | 4 | 78.45 | 1 |
| 73.55 | 7 | 78.5 | 5 |
| 73.6 | 5 | 78.55 | 1 |
| 73.65 | 5 | 78.6 | 2 |
| 73.7 | 11 | 78.65 | 2 |
| 73.75 | 5 | 78.7 | 3 |
| 73.8 | 7 | 78.75 | 1 |
| 73.85 | 7 | 78.8 | 1 |
| 73.9 | 8 | 78.85 | 3 |
| 73.95 | 13 | 78.9 | 2 |
| 74 | 11 | 78.95 | 3 |
| 74.05 | 13 | 79 | 0 |
| 74.1 | 17 | 79.05 | 2 |
| 74.15 | 11 | 79.1 | 2 |
| 74.2 | 15 | 79.15 | 3 |
| 74.25 | 7 | 79.2 | 7 |
| 74.3 | 16 | 79.25 | 6 |
| 74.35 | 14 | 79.3 | 5 |
| 74.4 | 10 | 79.35 | 4 |
| 74.45 | 8 | 79.4 | 2 |
| 74.5 | 9 | 79.45 | 3 |
| 74.55 | 9 | 79.5 | 2 |
| 74.6 | 6 | 79.55 | 4 |
| 74.65 | 8 | 79.6 | 4 |
| 74.7 | 9 | 79.65 | 1 |
| 74.75 | 6 | 79.7 | 3 |
| 74.8 | 9 | 79.75 | 0 |
| 74.85 | 3 | 79.8 | 5 |
| 74.9 | 2 | 79.85 | 3 |
| 74.95 | 7 | 79.9 | 1 |
| 75 | 11 | 79.95 | 3 |
| 75.05 | 11 | 80 | 2 |
| 75.1 | 8 |  |  |
| 75.15 | 14 |  |  |
| 75.2 | 8 |  |  |
| 75.25 | 8 |  |  |
| 75.3 | 2 |  |  |
| 75.35 | 15 |  |  |
| 75.4 | 8 |  |  |
| 75.45 | 10 |  |  |
| 75.5 | 8 |  |  |
| 75.55 | 11 |  |  |
| 75.6 | 6 |  |  |
| 75.65 | 7 |  |  |
| 75.7 | 13 |  |  |
| 75.75 | 8 |  |  |
| 75.8 | 11 |  |  |
| 75.85 | 9 |  |  |
| 75.9 | 9 |  |  |
| 75.95 | 4 |  |  |
| 76 | 3 |  |  |
| 76.05 | 5 |  |  |
| 76.1 | 7 |  |  |
| 76.15 | 7 |  |  |
| 76.2 | 5 |  |  |
| 76.25 | 8 |  |  |
| 76.3 | 8 |  |  |
| 76.35 | 4 |  |  |
| 76.4 | 4 |  |  |
| 76.45 | 5 |  |  |
| 76.5 | 5 |  |  |
| 76.55 | 5 |  |  |
| 76.6 | 4 |  |  |
| 76.65 | 1 |  |  |
| 76.7 | 5 |  |  |
| 76.75 | 5 |  |  |
| 76.8 | 4 |  |  |
| 76.85 | 4 |  |  |
| 76.9 | 6 |  |  |
| 76.95 | 8 |  |  |
| 77 | 7 |  |  |
| 77.05 | 5 |  |  |
| 77.1 | 7 |  |  |
| 77.15 | 7 |  |  |
| 77.2 | 13 |  |  |
| 77.25 | 7 |  |  |
| 77.3 | 9 |  |  |
| 77.35 | 5 |  |  |
| 77.4 | 4 |  |  |
| 77.45 | 10 |  |  |
| 77.5 | 5 |  |  |
| 77.55 | 9 |  |  |
| 77.6 | 7 |  |  |
| 77.65 | 2 |  |  |
| 77.7 | 13 |  |  |
| 77.75 | 12 |  |  |
| 77.8 | 8 |  |  |
| 77.85 | 17 |  |  |
| 77.9 | 21 |  |  |
| 77.95 | 20 |  |  |
| 78 | 12 |  |  |
| 78.05 | 13 |  |  |
| 78.1 | 12 |  |  |
| 78.15 | 8 |  |  |
| 78.2 | 11 |  |  |
| 78.25 | 11 |  |  |
| 78.3 | 7 |  |  |
| 78.35 | 8 |  |  |
| 78.4 | 6 |  |  |
| 78.45 | 8 |  |  |
| 78.5 | 6 |  |  |
| 78.55 | 5 |  |  |
| 78.6 | 1 |  |  |
| 78.65 | 6 |  |  |
| 78.7 | 5 |  |  |
| 78.75 | 8 |  |  |
| 78.8 | 3 |  |  |
| 78.85 | 7 |  |  |
| 78.9 | 4 |  |  |
| 78.95 | 2 |  |  |
| 79 | 7 |  |  |
| 79.05 | 8 |  |  |
| 79.1 | 9 |  |  |
| 79.15 | 10 |  |  |
| 79.2 | 8 |  |  |
| 79.25 | 5 |  |  |
| 79.3 | 4 |  |  |
| 79.35 | 12 |  |  |
| 79.4 | 10 |  |  |
| 79.45 | 11 |  |  |
| 79.5 | 6 |  |  |
| 79.55 | 5 |  |  |
| 79.6 | 14 |  |  |
| 79.65 | 6 |  |  |
| 79.7 | 5 |  |  |
| 79.75 | 9 |  |  |
| 79.8 | 12 |  |  |
| 79.85 | 9 |  |  |
| 79.9 | 4 |  |  |
| 79.95 | 8 |  |  |
| 80 | 8 |  |  |

**Table S3**. Source data for Fig. 3.

| **Data for Hep-G2** | | | | | | | | | | | | | | | | | | | | | | | | | | |
| --- | --- | --- | --- | --- | --- | --- | --- | --- | --- | --- | --- | --- | --- | --- | --- | --- | --- | --- | --- | --- | --- | --- | --- | --- | --- | --- |
| Fe_3_O_4_ nanoparticle | | | | | | | | | | | | | |  | | Fe_3_O_4_/ *Malva sylvestris* nanoparticles | | | | | | | | | | |
| Conce. | 500 | | 200 | | 100 | | 50 | | 10 | | 1 | | |  | | 500 | | 200 | | 100 | | 50 | | 10 | | 1 |
| Try 1 | 88.9821 | | 90.8812 | | 99.1711 | | 111.982 | | 118.908 | | 130.8111 | | |  | | 27.87879 | | 51.66667 | | 79.69697 | | 80.45455 | | 96.51515 | | 95.75758 |
| Try 2 | 86.0908 | | 92.8732 | | 100.6582 | | 103.4512 | | 119.4421 | | 132.3495 | | |  | | 25 | | 46.21212 | | 74.24242 | | 78.93939 | | 93.33333 | | 97.42424 |
| Try 3 | 90.3299 | | 91.5523 | | 110 | | 110.8621 | | 120.8504 | | 134.5129 | | |  | | 28.78788 | | 48.63636 | | 75 | | 85 | | 91.51515 | | 99.39394 |
| Mean | 88.4676 | | 91.7689 | | 103.2764 | | 108.7651 | | 119.7335 | | 132.5578 | | |  | | 27.22222 | | 48.83838 | | 76.31313 | | 81.46465 | | 93.78788 | | 97.52525 |
| SD | 2.165877 | | 1.01351 | | 5.870062 | | 4.635913 | | 1.003451 | | 1.859673 | | |  | | 1.977452 | | 2.732879 | | 2.954869 | | 3.154039 | | 2.530802 | | 1.820285 |
| **Data for MCF-7** | | | | | | | | | | | | | | | | | | | | | | | | | | |
| Fe_3_O_4_ nanoparticle | | | | | | | | | | | | |  | | Fe_3_O_4_/ *Malva sylvestris* nanoparticles | | | | | | | | | | | |
| Conce. | | 500 | | 200 | | 100 | | 50 | | 10 | | 1 |  | | 500 | | 200 | | 100 | | 50 | | 10 | | 1 | |
| Try 1 | | 89 | | 91 | | 100 | | 112 | | 119 | | 132 |  | | 35.90909 | | 41.51515 | | 49.75758 | | 63.48485 | | 67.87879 | | 72.87879 | |
| Try 2 | | 87 | | 93 | | 101 | | 105 | | 120 | | 130 |  | | 37.42424 | | 40.75758 | | 47.09091 | | 67.72727 | | 65.30303 | | 85.75758 | |
| Try 3 | | 90 | | 90 | | 103 | | 111 | | 123 | | 134 |  | | 34.09091 | | 42.12121 | | 52.42424 | | 63.48485 | | 71.66667 | | 80.75758 | |
| Mean | | 88.66667 | | 91.33333 | | 101.3333 | | 109.3333 | | 120.6667 | | 132 |  | | 35.80808 | | 41.46465 | | 49.75758 | | 64.89899 | | 68.28283 | | 79.79798 | |
| SD | | 1.527525 | | 1.527525 | | 1.527525 | | 3.785939 | | 2.081666 | | 2 |  | | 1.668961 | | 0.68322 | | 2.666667 | | 2.449365 | | 3.201 | | 6.492797 | |

**Table S4**. Data source for Fig. 4.

| ***Corynebacterium*** | | | | | | | | | | |
| --- | --- | --- | --- | --- | --- | --- | --- | --- | --- | --- |
|  | Fe_3_O_4_ nanoparticle | | | | | Fe_3_O_4_/ *Malva sylvestris* nanoparticles | | | | |
| Concentration | Try 1 | Try 2 | Try 3 | Mean | SD | Try 1 | Try 2 | Try 3 | Mean | SD |
| 1000 | -27.027 | -20.8494 | -26.6409 | 0.2 | 3.460574 | -39.7683 | -40.1544 | -43.2432 | 0.1 | 1.904588 |
| 500 | 3.861004 | -1.5444 | 5.405405 | 2.574003 | 3.649278 | -25.4826 | -15.0579 | -23.166 | 0.3 | 5.473918 |
| 250 | 28.18533 | 6.563707 | 22.00772 | 18.91892 | 11.13684 | 10.03861 | -2.7027 | -6.17761 | 0.3861 | 8.537971 |
| 125 | 68.72587 | 64.86486 | 65.63707 | 66.40927 | 2.043051 | 2.702703 | 7.335907 | 4.247104 | 4.761905 | 2.359112 |
| 62.5 | 76.44788 | 67.95367 | 74.51737 | 72.97297 | 4.452727 | -0.3861 | 13.89961 | 13.51351 | 9.009009 | 8.138693 |
| 31.25 | 77.22008 | 74.90347 | 80.69498 | 77.60618 | 2.914994 | 30.11583 | 27.79923 | 42.08494 | 33.33333 | 7.667115 |
| 15.62 | 96.5251 | 96.139 | 101.9305 | 98.1982 | 3.23803 | 67.18147 | 67.56757 | 61.77606 | 65.50837 | 3.23803 |
| 7.8 | 107.722 | 103.0888 | 91.89189 | 100.9009 | 8.138693 | 80.30888 | 84.55598 | 103.861 | 89.57529 | 12.55272 |
| ***Klebsiella* *pneumoniae*** | | | | | | | | | | |
|  | Fe_3_O_4_ nanoparticle | | | | | Fe_3_O_4_/*Malva sylvestris* nanoparticles | | | | |
| Concentration | Try 1 | Try 2 | Try 3 | Mean | SD | Try 1 | Try 2 | Try 3 | Mean | SD |
| 1000 | -1.92308 | -8.07692 | -12.6923 | 0.9 | 5.402899 | -31.1538 | -45.3846 | -31.1538 | 0.1 | 8.216138 |
| 500 | 6.153846 | 0.384615 | 2.307692 | 2.948718 | 2.937549 | -25.7692 | -25.3846 | -36.5385 | 0.2 | 6.331568 |
| 250 | 16.15385 | 20 | 14.61538 | 16.92308 | 2.773501 | 5.384615 | 1.538462 | -0.38462 | 2.179487 | 2.937549 |
| 125 | 46.53846 | 29.61538 | 23.46154 | 33.20513 | 11.94993 | 13.07692 | 5.384615 | 5.384615 | 7.948718 | 4.441156 |
| 62.5 | 56.92308 | 69.23077 | 58.46154 | 61.53846 | 6.705998 | 46.53846 | 40 | 41.15385 | 42.5641 | 3.489912 |
| 31.25 | 102.3077 | 93.84615 | 98.46154 | 98.20513 | 4.236593 | 68.46154 | 51.53846 | 66.15385 | 62.05128 | 9.177195 |
| 15.62 | 95.38462 | 94.61538 | 115 | 101.6667 | 11.55341 | 71.53846 | 80.76923 | 92.69231 | 81.66667 | 10.60544 |
| 7.8 | 101.1538 | 100.7692 | 110 | 103.9744 | 5.2219 | 93.46154 | 98.07692 | 112.6923 | 101.4103 | 10.03937 |
| ***Staphyloccus aureus*** | | | | | | | | | | |
|  | Fe_3_O_4_ nanoparticle | | | | | Fe_3_O_4_/*Malva sylvestris* nanoparticles | | | | |
| Concentration | Try 1 | Try 2 | Try 3 | Mean | SD | Try 1 | Try 2 | Try 3 | Mean | SD |
| 1000 | -31.0421 | -37.9157 | -25.8315 | 0.02 | 6.061169 | -30.4878 | -46.2306 | -33.592 | 0.01 | 8.338719 |
| 500 | -2.43902 | 3.658537 | 1.219512 | 0.813008 | 3.069038 | -8.86918 | -11.3082 | -10.3104 | 0.3 | 1.226213 |
| 250 | -4.43459 | 1.552106 | 12.86031 | 3.325942 | 8.782839 | 6.873614 | 6.54102 | 5.764967 | 6.3932 | 0.568914 |
| 125 | 11.3082 | 9.756098 | 11.52993 | 10.86475 | 0.966496 | 7.982262 | 9.090909 | 8.314856 | 8.462676 | 0.568914 |
| 62.5 | 24.39024 | 22.06208 | 22.61641 | 23.02291 | 1.216148 | 11.3082 | 8.093126 | 28.71397 | 16.03843 | 11.09442 |
| 31.25 | 38.3592 | 37.25055 | 38.80266 | 38.13747 | 0.799457 | 19.84479 | 19.29047 | 23.83592 | 20.99039 | 2.479837 |
| 15.62 | 56.31929 | 53.43681 | 59.7561 | 56.50407 | 3.163695 | 46.56319 | 40.35477 | 40.13304 | 42.35033 | 3.650128 |
| 7.8 | 77.49446 | 67.2949 | 86.25277 | 77.01404 | 9.488062 | 66.07539 | 79.60089 | 61.6408 | 69.10569 | 9.355653 |
| ***Pseudomonas aeroginosa*** | | | | | | | | | | |
|  | Fe_3_O_4_ nanoparticle | | | | | Fe_3_O_4_/*Malva sylvestris* nanoparticles | | | | |
| Concentration | Try 1 | Try 2 | Try 3 | Mean | SD | Try 1 | Try 2 | Try 3 | Mean | SD |
| 1000 | -8.00846 | -5.50918 | -3.8711 | 0.2 | 2.083563 | 3.076923 | 1.666667 | 1.410256 | 0.1 | 0.897436 |
| 500 | -5.12821 | -1.17949 | -1.28205 | 3.9 | 2.25077 | 4.641026 | 3.241769 | 3.776872 | 3.886556 | 0.706047 |
| 250 | 6.282051 | 2.641026 | 3.205128 | 4.042735 | 1.959708 | 7.890179 | 6.981215 | 7.412821 | 7.428072 | 0.454674 |
| 125 | 27.69231 | 41.66667 | 36.53846 | 35.29915 | 7.06913 | 11.71256 | 7.780023 | 9.001544 | 9.498042 | 2.012733 |
| 62.5 | 54.77128 | 61.71205 | 71.79981 | 62.76105 | 8.562596 | 31.87105 | 27.4933 | 31.67096 | 30.3451 | 2.471759 |
| 31.25 | 67.12821 | 76.53846 | 74.61538 | 72.76068 | 4.971737 | 59.98007 | 57.98121 | 49.90598 | 55.95575 | 5.333727 |
| 15.62 | 82.30769 | 79.09234 | 84.10982 | 81.83662 | 2.541692 | 85.76923 | 81.66667 | 75.25641 | 80.89744 | 5.298456 |
| 7.8 | 95.98092 | 92.98118 | 85.90113 | 91.62108 | 5.175707 | 92.30769 | 83.58974 | 93.20513 | 89.70085 | 5.311366 |

**Table S5**. Data source for Table 1.

| **Microorganisms** | **Fe_3_O_4_ nanoparticles (µg/mL)** | | **Fe_3_O_4_/** ***Malva sylvestris* nanoparticles (µg/mL)** | |
| --- | --- | --- | --- | --- |
|  | MIC | MBC | MIC | MBC |
| ***Staphylococcus aureus*** | 250 | 250 | 125 | > 125 |
| ***Corynebacterium*** | 500 | 1000 | 62.5 | 125 |
| ***Pseudomonas aeruginosa*** | 250 | 500 | 125 | > 125 |
| ***Klebsiella pneumoniae*** | 500 | 500 | 125 | > 125 |
